# Water Networks as Hydrophobic Recognition Motifs in Proteins

**DOI:** 10.1002/anie.202521138

**Published:** 2025-11-20

**Authors:** Serena G. Piticchio, Miriam Martínez‐Cartró, Salvatore Scaffidi, Sergio Rodríguez‐Arévalo, Andrea Bagán, Ainoa Sánchez‐Arfelis, Sarah Picaud, Tobias Krojer, Panagis Filippakopoulos, Frank von Delft, Carmen Escolano, Carles Galdeano, Xavier Barril

**Affiliations:** ^1^ Departament de Farmacia i Tecnología Farmacèutica, i Fisicoquímica Institut de Biomedicina (IBUB), Universitat de Barcelona Av. Joan XXIII, 27‐31, E‐08028 Barcelona Spain; ^2^ Laboratory of Medicinal Chemistry Department of Pharmacology, Toxicology and Medicinal Chemistry Faculty of Pharmacy and Food Sciences and Institute of Biomedicine (IBUB) University of Barcelona Av. Joan XXIII, 27‐31, E‐08028 Barcelona Spain; ^3^ Structural Genomics Consortium, Nuffield Department of Medicine Oxford University, Old Road Campus Research Building Roosevelt Drive Oxford OX3 7DQ UK; ^4^ Diamond Light Source Ltd. Harwell Science and Innovation Campus Didcot OX11 0QX UK; ^5^ Research Complex at Harwell Harwell Science and Innovation Campus Didcot OX11 0FA UK; ^6^ Centre for Medicines Discovery University of Oxford Oxford OX1 3QU UK; ^7^ Department of Biochemistry University of Johannesburg Auckland Park 2006 South Africa; ^8^ Catalan Institution for Research and Advanced Studies (ICREA) Barcelona 08010 Spain

**Keywords:** Interfacial water molecules, Medicinal chemistry, Molecular recognition, Protein‐ligand interactions

## Abstract

The hydrophobic effect is a central force in molecular recognition, typically attributed to the ordering of water molecules around apolar groups. Hydrophobic interaction sites on proteins are therefore readily predicted based on surface polarity. Yet, in the bromodomain‐containing protein 4 (BRD4), a well‐known hydrophobic hot spot is paradoxically lined by a network of water molecules. Here we combine binding assays, structural data, molecular dynamics, and free‐energy calculations to resolve this apparent contradiction. We show that the water network functions as a *hydrophobic recognition motif* that cannot accommodate polar groups without disruption. Instead, as the protein pre‐organizes the water network, apolar groups can bind with minimal entropic cost. In turn, they reinforce the surrounding hydrogen‐bond network, limiting the mobility of the entire protein–water assembly. With this perspective, we identify water networks potentially functioning as hydrophobic motifs in other pharmacological targets, revealing a general but overlooked recognition element with broad implications in drug discovery and protein design.

## Graphical abstract

Hydrophobic groups can strengthen confined water networks, promoting order that propagates through the protein structure and drives recognition, creating a paradoxical hydrophobic binding hot spot. Polar groups are rejected because they disrupt the network, destabilizing the protein structure.

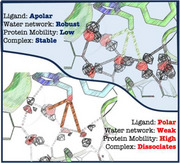

## Introduction

Water is the quintessential biological solvent. The fundamental characteristic of water molecules is their ability to form up to four hydrogen bonds, two as donor (through the hydrogen atoms) and two as acceptor (through the lone pairs). The four interactions are found in the structure of ice, while in liquid water the interactions are dynamic and the average number of hydrogen bonds per molecule is lower (quantifications range between two and four, depending on the experimental technique^[^ ^1^, ^2^ ^]^). The response to this polar environment determines, in large part, the structure and function of biomolecular systems. Polar and charged chemical moieties, such as glycans or the phosphate backbone of DNA, maximize their solvent exposure. Conversely, apolar groups tend to associate to minimize their direct contact with water. This is known as the hydrophobic effect, the driving force behind the formation of lipidic bilayers, folded proteins, protein‐ligand complexes and other biological structures. This effect can be largely captured with statistical approaches or by representing the solvent as a continuous medium with high dielectric response.^[^ ^3^, ^4^ ^]^ However, when discrete water molecules become trapped in particular environments, their behavior can be quite distinct from bulk. Depending on the shape and electrostatics of the solute, water molecules re‐arrange in ways and with consequences that can be difficult to predict. This is thoroughly investigated for materials such as minerals or hydrophobic polymers, where nano‐scale confinement changes the physicochemical properties of water, largely due to its ability to form hydrogen bonds.^[^ ^5^, ^6^ ^]^

Proteins create much smaller, complex and irregular surfaces, particularly in their binding sites. In such environments, the behavior of water has to be investigated at the level of individual molecules. In the unbound form, some hydration sites can be energetically unfavorable and displacing them can yield a significant increase in binding affinity.^[^ ^7^, ^8^, ^9^ ^]^ But other water molecules are still present in the ligand‐bound form of the binding site. They are considered an integral component of the tri‐dimensional structure of proteins and can play a major role in the recognition of substrates and ligands.^[^ ^10^, ^11^ ^]^ This is fairly common in protein‐ligand complexes and owing to its importance for molecular design and drug discovery, an abundance of methods have been developed to predict hydration sites and water displaceability.^[^ ^12^, ^13^, ^14^, ^15^ ^]^ However, little emphasis has been placed on understanding the molecular recognition properties of protein‐bound water molecules, assuming that they simply act as hydrogen bond donors and/or acceptors.

Here we investigate a network of water molecules in the binding site of the bromodomain‐containing protein 4 (BRD4), which become confined upon ligand binding. Empirical evidence suggests that, surprisingly, the waters create a hydrophobic binding hot spot around them. We generate a series of molecules that differ only on the polarity of the group in contact with the water network and characterize their interaction with BRD4 using biophysical, structural and computational techniques. This allows us to confirm, quantify and rationalize this counterintuitive behavior of a water network. Due to its paradoxical nature, this molecular recognition motif has thus far been overlooked and merits further investigation.

## Results and Discussion

Bromodomains are small protein domains that selectively recognize acetyl‐lysine, a posttranslational modification of lysine that is particularly important in the context of epigenetics, particularly as transcription‐activating signals coded in the histone tails. As such, bromodomains have been actively pursued as targets for the treatment of cancer.^[^ ^16^ ^]^ They are present in 46 human proteins and despite a large sequence variability, their 3D structure is well conserved.^[^ ^17^ ^]^ We will investigate the first bromodomain of BRD4 (i.e., BRD4(1)), as this is the best characterized. BRD4(1) recognizes its substrate through the formation of a hydrogen bond between Asn140 and the carbonyl moiety of acetyl lysine, whereas the methyl part of acetyl lysine is placed at the bottom of the cavity. Intriguingly, the methyl is in direct contact (<4 Å) with three water molecules that form no polar contact with the ligand (Figure S1). These waters are part of a larger hydration network that is consistently observed in the crystal structures of BRD4(1). Their preference to interact with hydrophobic substituents is further confirmed by analyzing a diverse set of ligands^[^ ^18^ ^]^ (Figure S1). While the displaceability of the structural water molecules has been thoroughly investigated,^[^ ^19^, ^20^ ^]^ no explanation has been put forward to justify the hydrophobic character of the water network.

**Figure 1:**
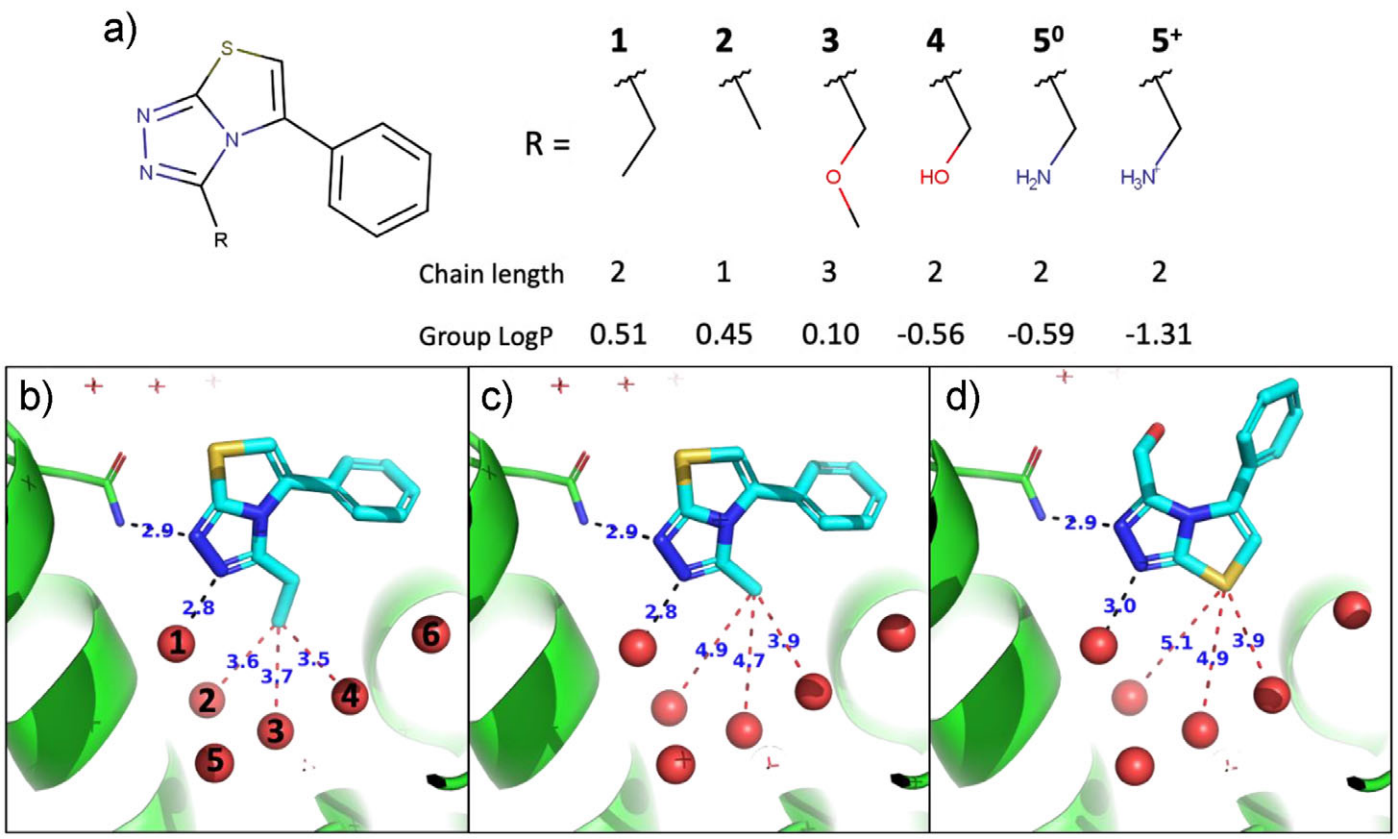
a) Ligand series with the different R groups directed to the methyl binding subpocket, where they can interact with the water network. **1**: Ethyl; **2**: Methyl; **3**: Methoxymethyl; **4**: Methyl alcohol; **5^0^**: Methyl amine; **5^+^**: Methyl ammonium. Group LogP is calculated from the atomic contributions of Wildman and Crippen.^[^ ^22^ ^]^ b) Crystal structure of **1**, indicating the water nomenclature used here. c) Crystal structure of **2**. d) Crystal structure of **4**.

Compound **1** is a BRD4(1) ligand that we previously identified using an automated computational approach. In line with what has been observed for other ligands, it places the ethyl group in the methyl binding subpocket of BRD4(1), making close contacts with the water network (Figure 1).^[^ ^21^ ^]^ Our synthetic protocol (see Methods) allowed us to substitute the ethyl group of **1** with isosteric functional groups of increased polarity (alcohol and amine), as well as bigger and smaller hydrophobic groups (Figure 1a). The dominant protonation state of **5** at physiological pH is the charged amine, but as the predicted pKa is close to the experimental pH of 7.4 (Figure S2), we will consider both microspecies in the simulations (referred to as **5^0^** and **5^+^**).

We then proceeded to determine the binding affinity of these compounds in a TR‐FRET assay to investigate their relative binding affinities (Table 1 and Figure S3). We also obtained the crystal structures of BRD4(1) in complex with **2** and **4** (Table S1). The binding affinity data suggest a relatively flat SAR, with respect to shape where shortening (**2**) or lengthening (**3**) the alkyl chain produces a 2‐fold and 3‐fold loss of efficacy with respect to **1**, respectively. The X‐ray crystallography structure of **2** (Figure 1c) shows that the ligand barely changes its binding mode compared to **1**. As a result, the average distance of the terminal methyl to the three surrounding water molecules increases from 3.6Å to 4.5Å. The scarce change in binding affinity despite creation of packing defect indicates that there is no specific interaction between the methyl and the water molecules. This behavior resembles hydrophobic pockets, where optimal occupation is often achieved before the van der Waals limit is reached.^[^ ^23^ ^]^ Molecular Dynamics (MD) simulations of **3** suggest that chain extension has a minimal impact on the water network, but pushes the triazole core away from its optimal position(Figure S4).

**Table 1:** Binding affinities determined experimentally by TR‐FRET and relative binding affinities calculated with FEP. Note that computational and experimental results for **4** and **5** are not comparable because they stem from different binding modes.

**Compound**	**TR‐FRET** IC_50_ *(fold change)*		**FEP** *ΔΔG*_bind_ *(K*_D_ *fold change)*
**1 (reference)**	26 µM *(1x)*		0.0 (1x)
**2**	54 µM *(2x)*		+0.8 kcal/mol (4x)
**3**	85 µM *(3.3x)*		n.d.
**4**	65 µM *(2.5x)*		+2.5 kcal/mol (65x)
**5**	157 µM *(6x)*	**5^0^**	+3.3 kcal/mol (248x)
		**5^+^**	+10.3 kcal/mol (30,000x)

**Figure 2:**
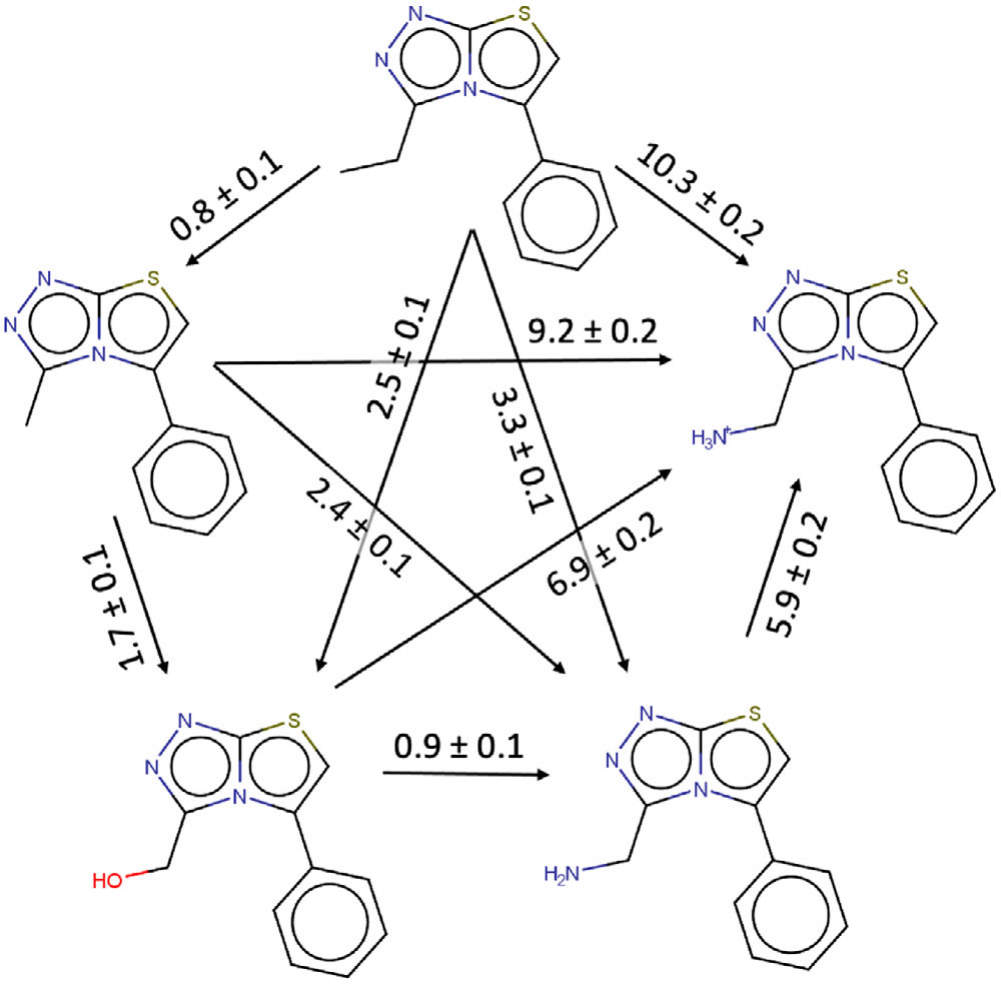
Relative Free Energies of the series, predicted by FEP calculations. The arrows indicate the direction of transformations.

Replacing the methyl in **1** with isosteric polar groups, (hydroxyl in **4** and amine in **5**), leads to a loss of affinity, but only by a factor of 2.5‐fold and 6‐fold, respectively. However, to our surprise, compound **4** displays a reversed binding mode, exposing the polar group to bulk solvent, and placing the sulphur atom of the thiazole ring, which is hydrophobic (atomic logP = 0.62 ^[^ ^22^ ^]^), adjacent to the water network (Figure 1d). This indicates that a polar substituent is repulsed to the extent of causing a complete reorientation of the molecule. Note that the reorientation cannot be justified by a gain of interaction of the newly introduced hydroxyl, as it actually shows a solvent‐exposed orientation and does not form a hydrogen‐bond with Asn40 (Figure S5). While we could not obtain a crystal structure for **5**, it seems logical to assume that it also changes its binding mode. These results confirm that the water network creates a hydrophobic binding spot, but complicates its experimental quantification. For this reason, we turn to free energy perturbation (FEP) calculations with MD simulations in explicit water solvation. This is a well‐stablished method to obtain quantitative predictions of relative binding affinities.^[^ ^24^ ^]^ In this case, we restrain the ligands to ensure that the original binding mode is preserved throughout the simulation. Individual FEP transformations have very low uncertainties and cycle closures in the FEP transformation network afford minimal errors, providing high confidence in the predicted values (Figure 2). The predicted binding free energies relative to **1** are shown in Table 1.

According to these computational results, replacing the terminal methyl of **1** ‐ which is in direct contact with the water network–with isosteric neutral polar substituents would have a large free energy cost (2.5 kcal/mol for alcohol (**4**); 3.3 kcal/mol for neutral amine (**5^0^**)). Introducing a charged amino group (**5^+^**) would be penalized by 10 kcal/mol. These results not only confirm the hydrophobic nature of the water network, but also reveal a surprisingly high selectivity factor. This makes sense from a functional perspective, as bromodomains specifically recognize acetylated lysine over the much more abundant unmodified lysine, but the molecular mechanism is certainly intriguing.

To shed some light on the counterintuitive behavior of the water network, we perform MD simulations of BRD4(1) in the unbound form and in complex with each ligand, analyzing the water densities at the hydration sites (Figure S6), which are converted to binding free energy values in Table 2. We also perform GIST energetic decomposition^[^ ^12^, ^14^ ^]^ to determine the intrinsic preference of the water molecules (Table 2 and Table S2). The water network is already very favorable in the unbound state, with average *ΔG*_bind_ values per water molecule of −0.92 kcal/mol (entire network) and −1.47 kcal/mol (three waters lining the methyl binding pocket). Binding of **1** isolates the water network from bulk solvent, which has a major stabilizing effect on the entire network (average *ΔG*_bind_ of −1.42 kcal/mol per hydration site) and on the three “hydrophobic” waters (average *ΔG*_bind_ of −1.73 kcal/mol). This trend is reversed in the presence of compound **4**, which also isolates the water network, but places a hydroxyl in the methyl binding subpocket (average *ΔG*_bind_ of −1.24 kcal/mol and −1.47 kcal/mol per hydration site on the entire network and the subpocket, respectively). Finally, the presence of the charged group in compound **5^+^** results in a major reduction in water densities, even compared to the ligand‐free state (average ΔG_bind_ of −0.72 kcal/mol and −1.12 kcal/mol per hydration site on the entire network and the subpocket, respectively). Interestingly, the GIST energetic decomposition indicates that the intrinsic preference of water molecules runs in exactly the opposite direction: compound **1** leaves the enthalpic term practically unchanged relative to the unbound state, but introduces a substantial entropic penalty, as the water molecules become better oriented in presence of the apolar moiety. The end result is a predicted *ΔG*_bind_ loss of 0.68 kcal/mol per hydration site relative to the unbound form. Compared to **1**, compounds **4** and **5^+^** improve both the enthalpy and the entropy of the water molecules, as their polar groups offer favorable interactions with the water molecules and allow for a more dynamic water network. For the three water molecules in the methyl binding site, GIST predicts that replacing the methyl in **1** with the hydroxyl in **4** improves the *ΔG*_bind_ by an average of −0.83 kcal/mol per hydration site. The equivalent value for introducing a charged amino group (compound **5^+^**) is −4.5 kcal/mol.

**Table 2:** Thermodynamic properties of hydration sites 2, 3, and 4 (as indicated in Figure 1a) and the entire network of nine water molecules. MD‐observed *ΔG*_bind_ is the free energy of water binding, derived from the MD‐observed densities. The GIST parameters are normalized per water molecule and reflect the intrinsic energy/entropy change relative to bulk solvent. Values relative to the most stable complex (BRD4(1)‐**1**) are indicated in parentheses. All values in kcal/mol. Table S2 provides values for each individual water and further decomposition of the GIST terms.

		MD‐observed	GIST		
		*ΔG*_bind_	*ΔH*_bind_	−*ΔTS*_bind_	*ΔG*_bind_
Unbound	W2	−1.5 *(+0.3)*	−5.8 *(−0.5)*	3.8 *(−0.6)*	−2.0 *(−1.1)*
	W3	−1.4 *(+0.2)*	−5.3 *(+0.3)*	3.8 *(−0.4)*	−1.6 *(−0.1)*
	W4	−1.5 *(+0.3)*	−6.5 *(+0.0)*	3.5 *(−0.7)*	−3.1 *(−0.8)*
	$\sum_{i=2}^{4}W_{i}$	**−4.4 *(+0.8)***	**−17.6 *(−0.2)***	**11.1 *(−1.8)***	**−6.7 *(−2.0)***
	$\sum_{i=1}^{9}W_{i}$	**−8.2 *(+4.6)***	**−57.3 *(+1.1)***	**22.2 *(−7.2)***	**−35.1 *(−6.1)***
1	W2	−1.8 *(0.0)*	−5.3 *(0.0)*	4.4 *(0.0)*	−0.9 *(0.0)*
	W3	−1.6 *(0.0)*	−5.7 *(0.0)*	4.2 *(0.0)*	−1.5 *(0.0)*
	W4	−1.8 *(0.0)*	−6.5 *(0.0)*	4.2 *(0.0)*	−2.3 *(0.0)*
	$\sum_{i=2}^{4}W_{i}$	**−5.2 *(0.0)***	**−17.5 *(0.0)***	**12.8 *(0.0)***	**−4.7 *(0.0)***
	$\sum_{i=1}^{9}W_{i}$	**−12.8 *(0.0)***	**−58.4 *(0.0)***	**29.4 *(0.0)***	**−29.0 *(0.0)***
4	W2	−1.5 *(+0.3)*	−5.7 *(−0.4)*	4.0 *(−0.4)*	−1.7 *(−0.8)*
	W3	−1.4 *(+0.2)*	−5.8 *(−0.1)*	3.8 *(−0.4)*	−2.0 *(−0.5)*
	W4	−1.5 *(+0.3)*	−7.3 *(−0.8)*	3.8 *(−0.4)*	−3.6 *(−1.3)*
	$\sum_{i=2}^{4}W_{i}$	**−4.4 *(+0.8)***	**−18.8 *(−1.3)***	**11.6 *(−1.2)***	**−7.2 *(−2.5)***
	$\sum_{i=1}^{9}W_{i}$	**−11.2 *(+1.6)***	**−58.7 *(−0.4)***	**26.9 *(−2.4)***	**−31.8 *(−2.8)***
5^+^	W2	−1.5 (+0.3)	−9.4 (−4.1)	3.3 (−1.1)	−6.1(−5.2)
	W3	−1.0 (+0.6)	−8.4 (−2.7)	2.9 (−1.3)	−5.5 (−4.0)
	W4	−0.8 (+1.0)	−9.2 (−2.7)	2.6 (−1.6)	−6.6 (−4.3)
	$\sum_{i=2}^{4}W_{i}$	**−3.3 *(+1.9)***	**−27.0 *(−9.5)***	**8.8 *(−4.0)***	**−18.2 *(−13.5)***
	$\sum_{i=1}^{9}W_{i}$	**−6.5 *(+6.3)***	**−62.2 *(−3.8)***	**19.3 *(−10.1)***	**−42.9 *(−13.9)***

**Figure 3:**
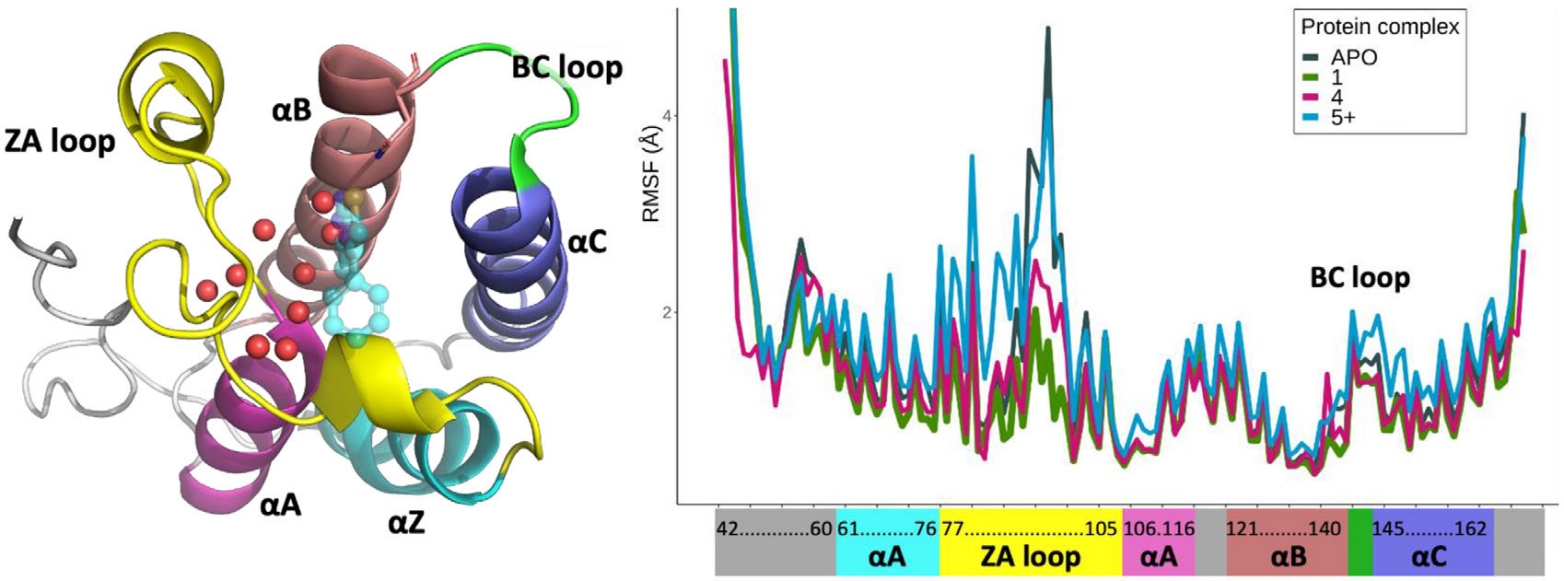
Structural elements of the BRD4(1) domain (left) and MD‐derived RMSF per residue in the ligand‐free state and bound to compounds **1**, **4,** and **5^+^** (right).

It must be noted that the *ΔG*_bind_ values obtained by MD and GIST are not in contradiction: the former takes into consideration the entire system, while the latter indicates the intrinsic energetics of a water molecule at a particular hydration site, relative to bulk. Results indicate that the water molecules would prefer a more polar environment, but they are part of a larger system that, globally, selects for hydrophobic groups. The enthalpic and entropic penalty paid by the water molecules is part of a trade‐off that, as the experimental results indicate, ultimately results in a lower free energy state. This state manifests itself as a global change in protein dynamics. Indeed, in our MD simulations we observe that binding of hydrophobic ligands results in a global decrease of protein dynamics, particularly reducing the mobility of the BC and ZA loops of BRD4. Polar and charged ligands exert the opposite effect (Figure 3 and Figure S7). The change in dynamics is directly linked to the robustness of the water network, because it connects multiple residues that are distant in sequence but close in space (Figure S8). In summary, placing a hydrophobic group around the hydration sites orders and strengthens the water network, tightening the connection between structural elements, and shifting the protein towards a lower‐mobility state.

Taken together, these analyses point to a curious mechanism for selective recognition of hydrophobic ligands in the methyl binding subpocket of BRD4(1): Polar groups form favorable interaction with the water molecules, but destabilize the water network and the entire protein structure, resulting in rapid ligand dissociation. Apolar groups form unfavorable interaction with the water molecules, but shields them from competing hydrogen bonds and creates a lower dielectric environment that reinforces the network of hydrogen bonds (as confirmed by neutron protein crystallography^[^ ^25^ ^]^), organizing not only the water network, but also the protein core. This reorganization offers a possible explanation for the characteristic enthalpic signatures observed in BRD4(1) ligands,^[^ ^25^, ^26^ ^]^ and is consistent with reports that the conformation of the ZA loop is critical for predicting enthalpic contributions to ligand binding.^[^ ^27^ ^]^

Precedents for this behavior exist in studies probing the enthalpy–entropy balance of inhibitor series that differ only in their solvent‐exposed groups. Hydrophobic substituents of particular shapes have been shown to promote the formation of ordered water networks at solute–solvent interfaces, resulting in enthalpy‐driven binding.^[^ ^28^, ^29^, ^30^ ^]^ Despite the striking structural and thermodynamic evidence for such water structuring, the overall impact on affinity was found to be modest due to enthalpy–entropy compensation.

Further insight into how water networks can act as hydrophobic hot spots comes from the work of Mochizuki and Molinero, who demonstrated that antifreeze glycoprotein 8 binds to ice through adsorption of methyl groups.^[^ ^31^ ^]^ In this case, binding is driven by the entropy gain associated with replacing the solvation shell around hydrophobic groups with pre‐organized water at the ice surface. This example illustrates that water networks can—paradoxically—function as hydrophobic recognition motifs, provided they are pre‐organized by their environment: in the solid‐state lattice for ice, and in our case, by the protein itself.

Surprised by the apparent uniqueness of the recognition motif, we performed a cursory examination of historical therapeutic targets. In phosphodiesterase 4 (PDE4), two metal ions (Zn^+2^ and Mg^+2^) create a highly ordered and coordinated water network. There are examples in the literature of researchers trying to either displace or form polar contacts with the water network, which turned out to be possible, but difficult.^[^ ^32^, ^33^, ^34^ ^]^ Yet, many potent ligands place aliphatic or aromatic carbon atoms in close contact with the water network, suggesting that these are favorable interactions (Figure S9). Confirming this assumption, the phenyl ring of ligand 3DE, which is in direct contact with 5 water molecules, increases affinity for PDE4D from 82 µM to 270 nM (*ΔΔG*_bind _= 3.4 kcal/mol)(Figure S10).^[^ ^35^ ^]^ Putative hydrophobic water networks in β‐Site Amyloid Cleaving Enzyme 1 (BACE1) are discussed in Supporting Information. A systematic re‐examination of historical structural data is likely to reveal further examples, extending beyond protein–ligand complexes.

## Conclusion

The role of water molecules in the formation of protein‐ligand complexes has been widely explored, but typically in terms of whether they should be displaced or engaged through polar interactions. Our investigation opens up a third and overlooked possibility: stabilizing the water network by placing hydrophobic groups around them. When such networks are tightly coupled to the protein, this may result in favorable recognition.

This paradoxical mechanism arises from systemic effects. Although water molecules intrinsically favor polar interactions, confinement alongside a low‐dielectric apolar ligand reinforces pre‐existing hydrogen bond networks. The resulting order propagates into the protein scaffold, reducing its mobility and stabilizing the bound state. This systemic effect is inextricable from the hydrophobicity of the water network and highlights its potential as a regulatory mechanism.

The finding that water networks in proteins can function as hydrophobic hot spots has broad implications. While demonstrated in BRD4, analogous features are apparent in PDE4 and BACE1, indicating that this is not an isolated phenomenon but a general, underappreciated recognition motif. Although we have focused on protein–ligand complexes, hydrophobic water networks are well suited to couple binding with conformational dynamics and may underlie other protein functions. Incorporating this concept into the toolbox of molecular design could open new avenues in drug discovery, protein engineering, and structural and chemical biology.

## Conflict of Interests

The authors declare no conflict of interest.

## Supporting information



Supporting Information

## Data Availability

The data that support the findings of this study are available in the Supporting Information of this article.
