# Supplementary material for: Water Networks as Hydrophobic Recognition Motifs in Proteins
Source: Angew Chem Int Ed Engl. 2025 Nov 20;65(2):e21138. doi: 10.1002/anie.202521138 (PMC12790326; doi:10.1002/anie.202521138)
Supplement: Supplementary file 1 — Supporting Information [file ANIE-65-e21138-s001.pdf]

Supplementary Information for:

Water Networks as Hydrophobic Recognition Motifs in Proteins.

Table of Contents

Supplementary Figures 2

Supplementary Tables 10

Putative hydrophobic water networks in BACE1 14

Methods 17

Protein Production 17

Binding assay 17

Structural Determination 18

Molecular Dynamics 18

Synthesis 21

^{1}H-NMR and ^{13}C-NMR spectra of new compounds 25

HPLC/MS analysis of new compounds 34

Molecular Formula Strings (SMILES) 42

REFERENCES 42

Supplementary Figures

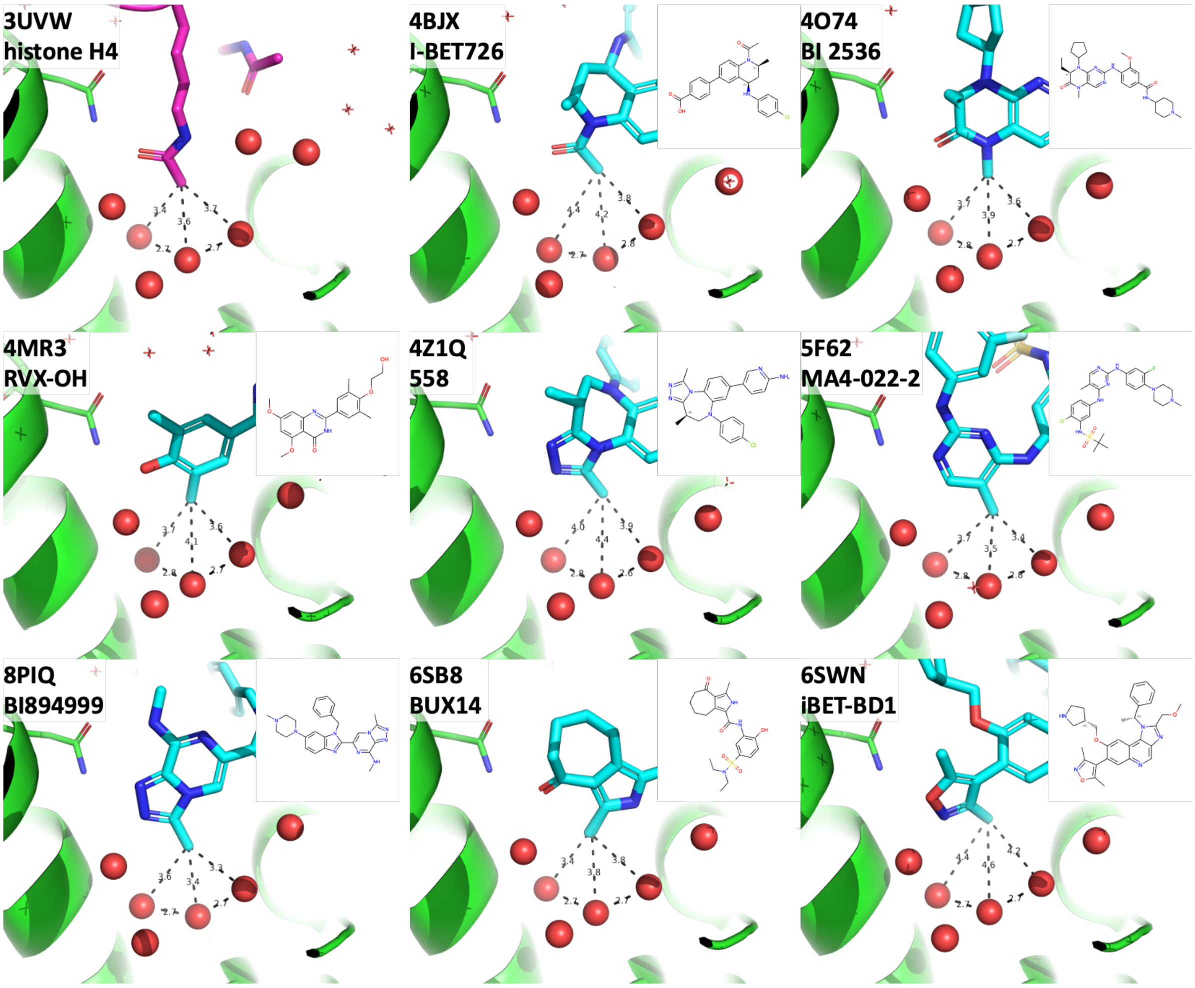

Figure S1: Experimental binding mode of histone H4 and eight diverse and potent BRD4(1) inhibitors, highlighting the contacts between their methyl group and 3 water molecules. Such conserved interaction motif across structurally unrelated ligands indicates that this is an intrinsically favourable interaction. PDB codes and ligand names are indicated on the top-left corner of each image. Citations: [1–8] (8PIQ remains unpublished).

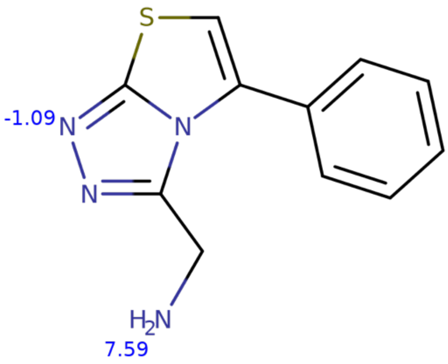

Microspecies at pH 7.4	
**5⁰** (NH_2)	39%
**5⁺** (NH_3^+)	61%

Figure S2: Calculated pKa of compound **5** (source: chemicalize.com from ChemAxon). The amine derivative can exist in two states at physiological pH, both of which have been considered in this study.

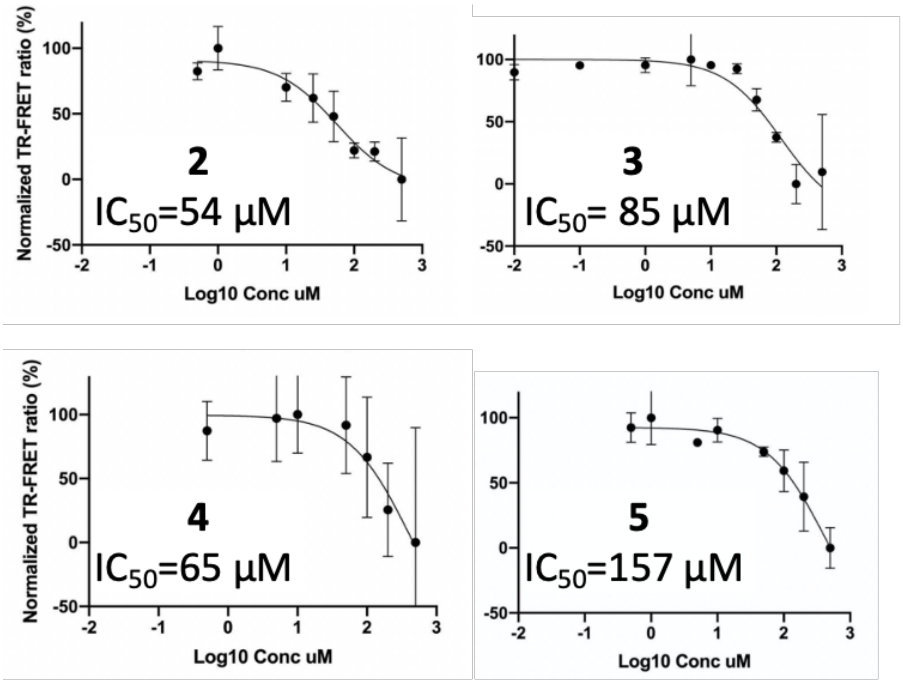

Figure S3: Normalized TR-FRET assay showing dose-dependently displacement of a tetra-acetylated H4 peptide from Brd4(BD1) in the presence of compounds **2**-**5**. IC_{50} values corresponding to best fit are reported inside each graph. Data for compound 1 is reported in Reference [9] and was performed simultaneously. Each dot represents the average of two independent replicas, and the error bars correspond to their standard deviation.

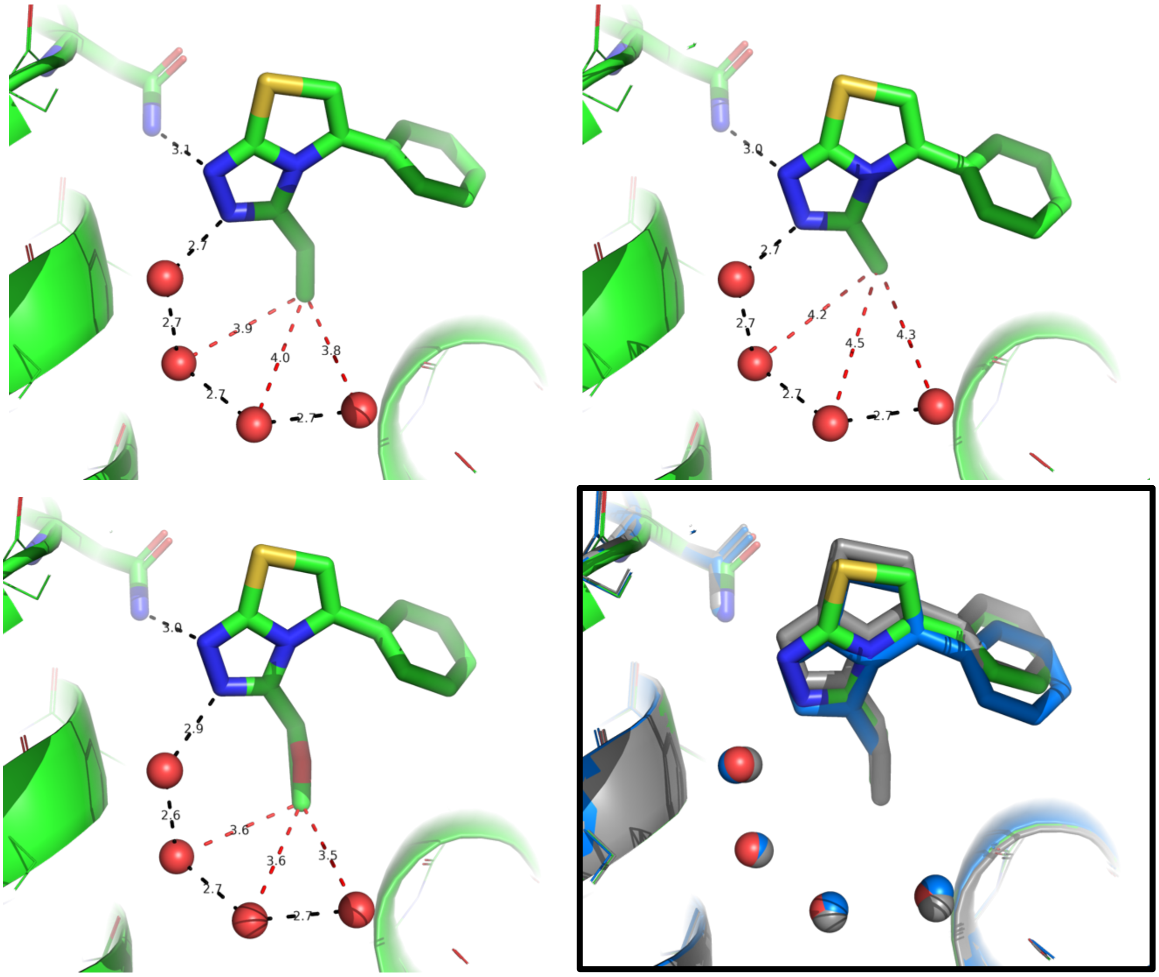

Figure S4. Average MD geometries of the compounds with hydrophobic chains: **1** (top left), **2** (top right) & **3** (bottom left). Water molecules have been placed at the site of maximal density for each simulation. The bottom-right panel shows the superimposition of all 3 structures, where **2** is shown in blue and **3** in grey. For compound **3**, the MD shows that chain lengthening causes a small (downward) displacement of the optimal position of W3 and W4 in the network, and a larger (upward) displacement of the triazole ring, resulting in suboptimal geometry. The average Methyl-water distances range from 4.3Å (**2**), to 3.9 Å (**1**) and 3.6 Å (**3**). As reference, the sum of the respective van der Waals radii is 3.5 Å. Note that all MD simulations start from the experimental geometry of compound **1** (PDB code 6ZF9), changing only the R-group (see Methods).

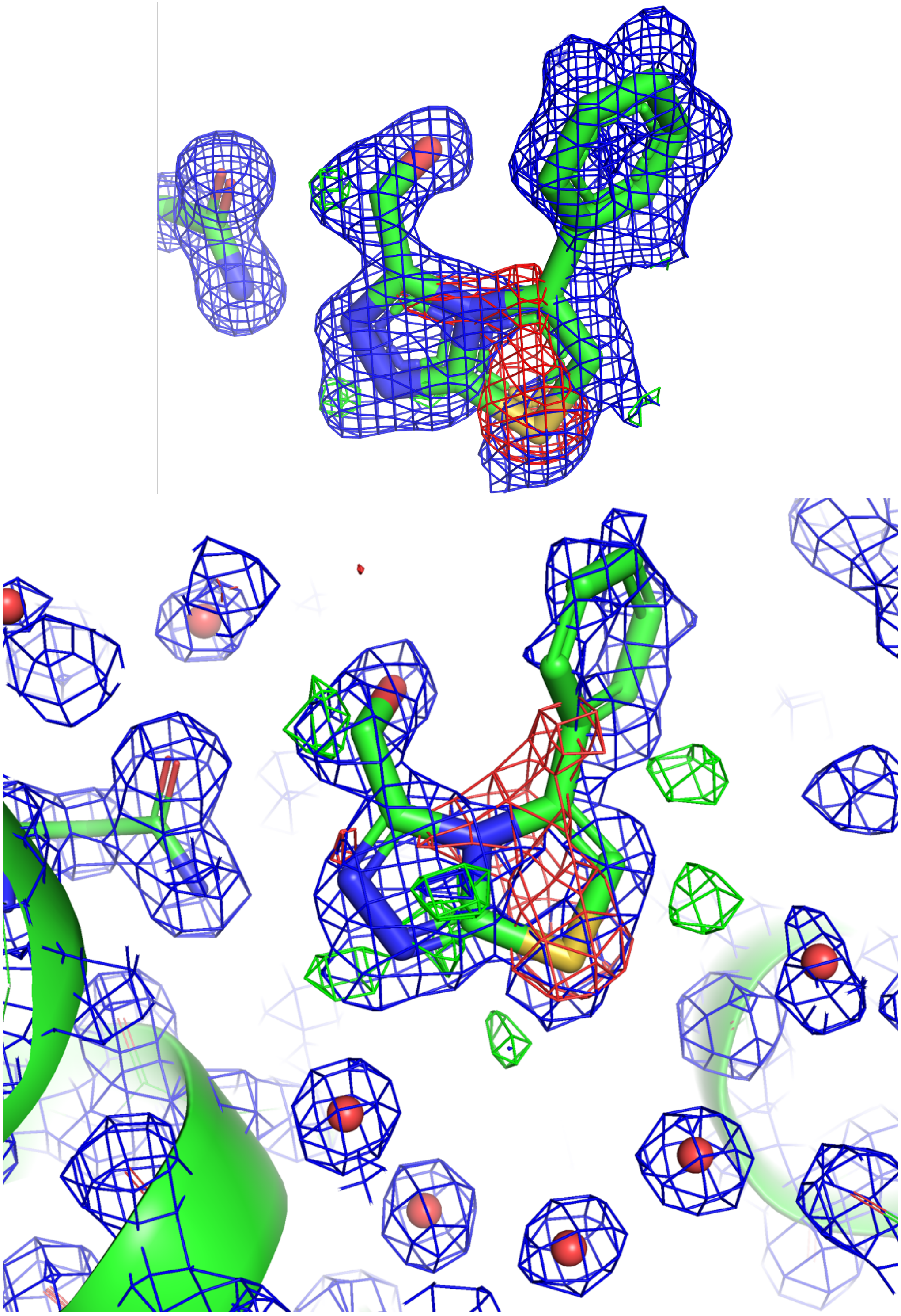

Figure S5. Electron density map of BRD4(1) in complex with compound **4**. The top figure shows the refined $2mF_o–DF_c$ electron map (blue, 1σ) and $mF_o–DF_c$ difference map (green/red, ±3σ) around compound **4**, Asn140 omitting everything else for clarity. The bottom figure shows the same densities for the ligand and the binding site, including the water network.

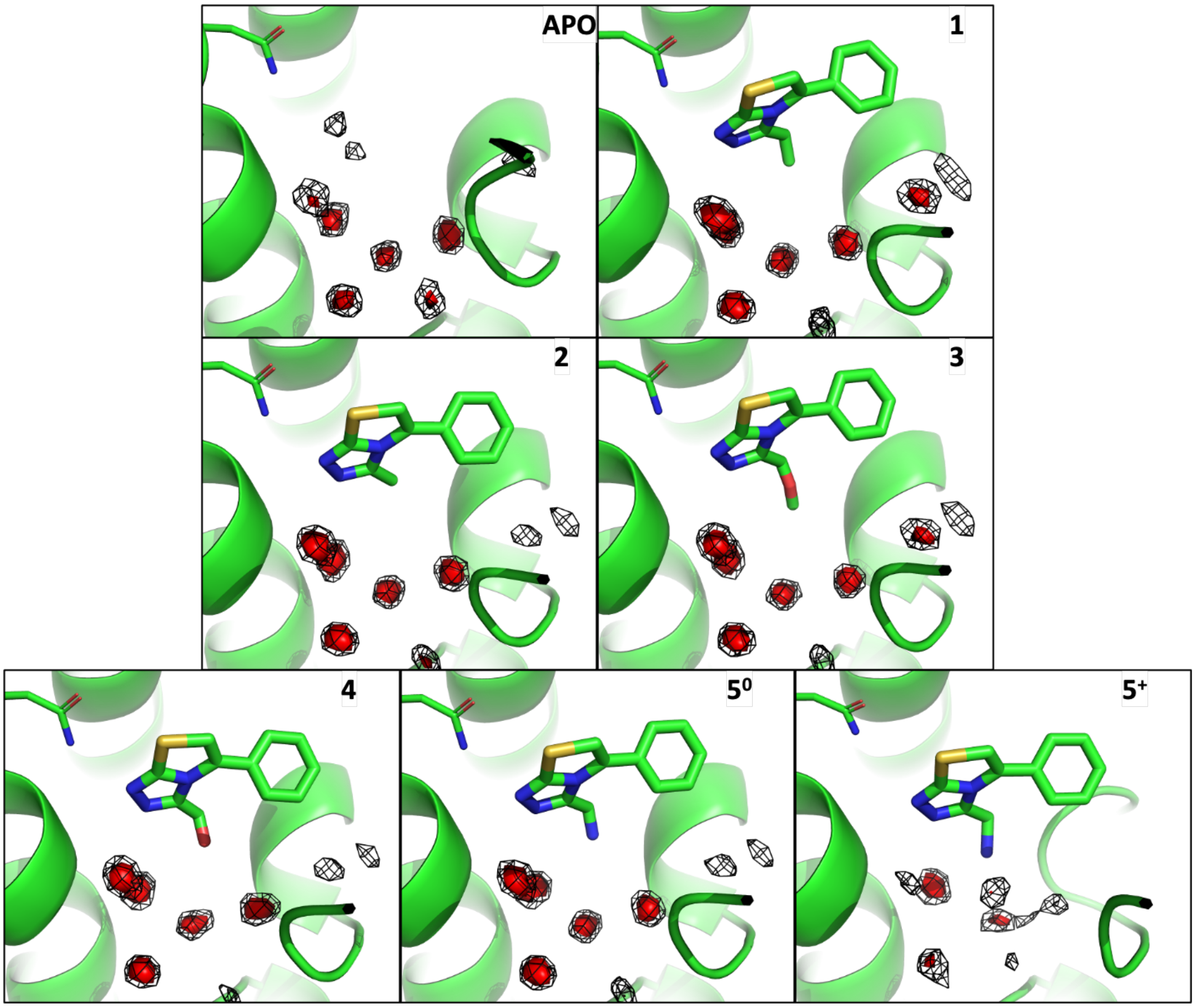

Figure S6. Qualitative analysis of the influence of various ligands on the BRD4(1) water network. The density of water oxygen is shown as red surface (10g, ΔG_{bind}=-1.37 kcal/mol) and black mesh (5g, ΔG_{bind}=-0.96 kcal/mol). Densities and geometries correspond to averages over unrestrained MD trajectories. The presence of ligands reinforces the water densities relative to the unbound state, except for ligand **5+**, which completely disrupts the network. Table S2 provides a quantitative analysis. Note that all MD simulations start from the experimental geometry of compound **1** (PDB code 6ZF9), changing only the R-group (see Methods).

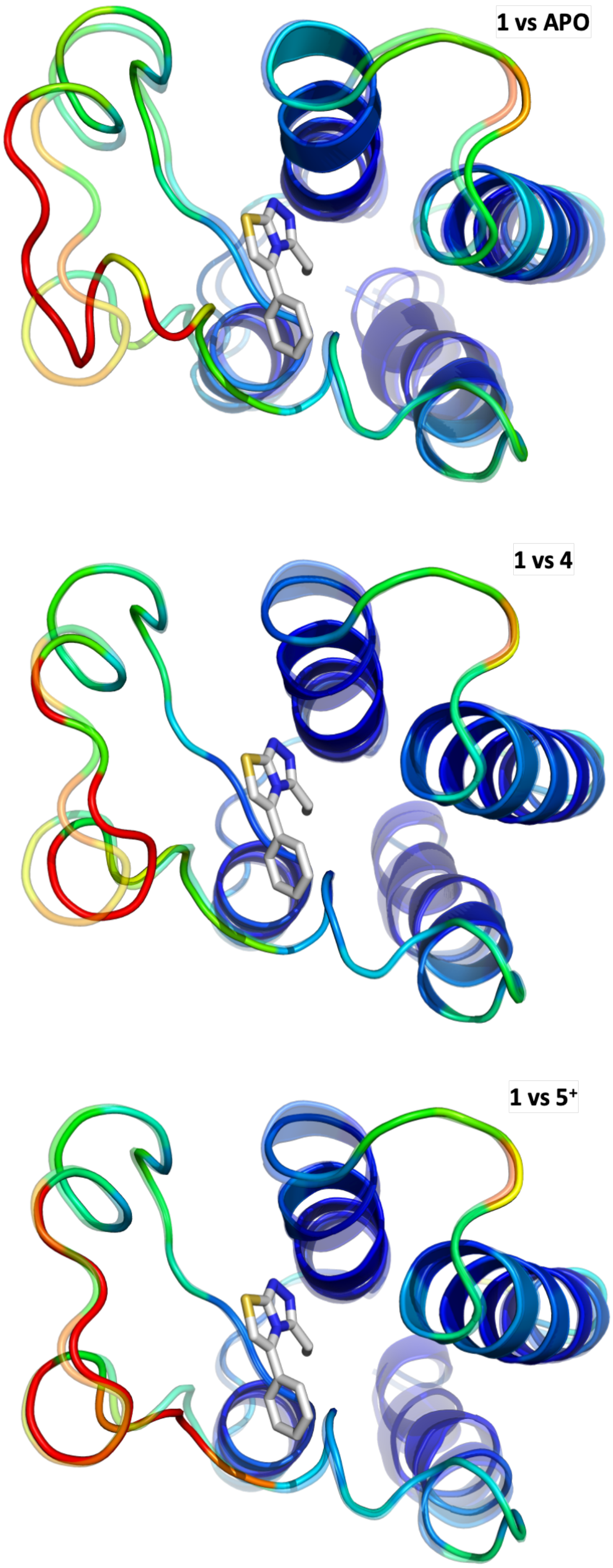

Figure S7. MD-derived average geometries of the BRD4(1) backbone with representative ligands, coloured by RMSF of the Cα (blue=0.5Å to red=1.5Å). The structure of the complex with ligand **1** is shown as reference (semi-transparent cartoon, grey ligand). Comparatively warmer colours in the loop regions of unbound (top), **4**-bound (middle) and **5**$^{+}$-bound (bottom), indicate that they are more mobile than **1**-bound BRD4. Note that all MD simulations start from the experimental geometry of compound **1** (PDB code 6ZF9), changing only the R-group (see Methods).

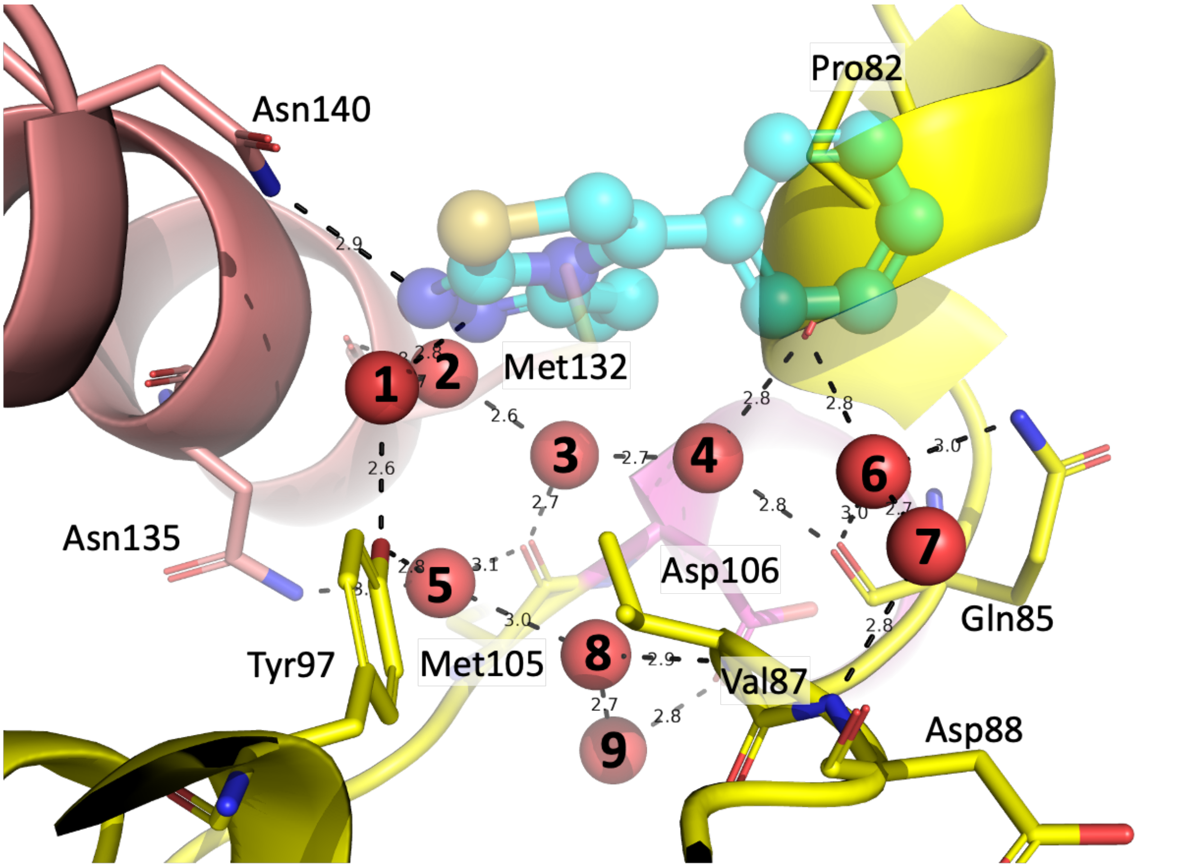

Figure S8: Detailed view of the BRD4(1) complex with **1** (PDB code 6ZF9). The water network connects multiple structural elements that converge on the binding site: W4 and W6 form hydrogen bonds with Pro82 and Gln85, two residues in different secondary structure elements at each side of the so-called ZA-channel; W7 and W8 form hydrogen bonds with the backbone of Asp88 and Val87, respectively, located at the beginning of the ZA loop; W1 and W5 hydrogen bond with the side-chain of Tyr97, in the middle of the ZA loop; W3, W5 and W9 interact with Met105 and Asp106, at the beginning of Helix αA; and W2 and W5 form hydrogen bonds with Met132 and Asn135, in Helix αB. Additionally, W2 interacts with the ligand, which is also connected to Asn140, in Helix αB.

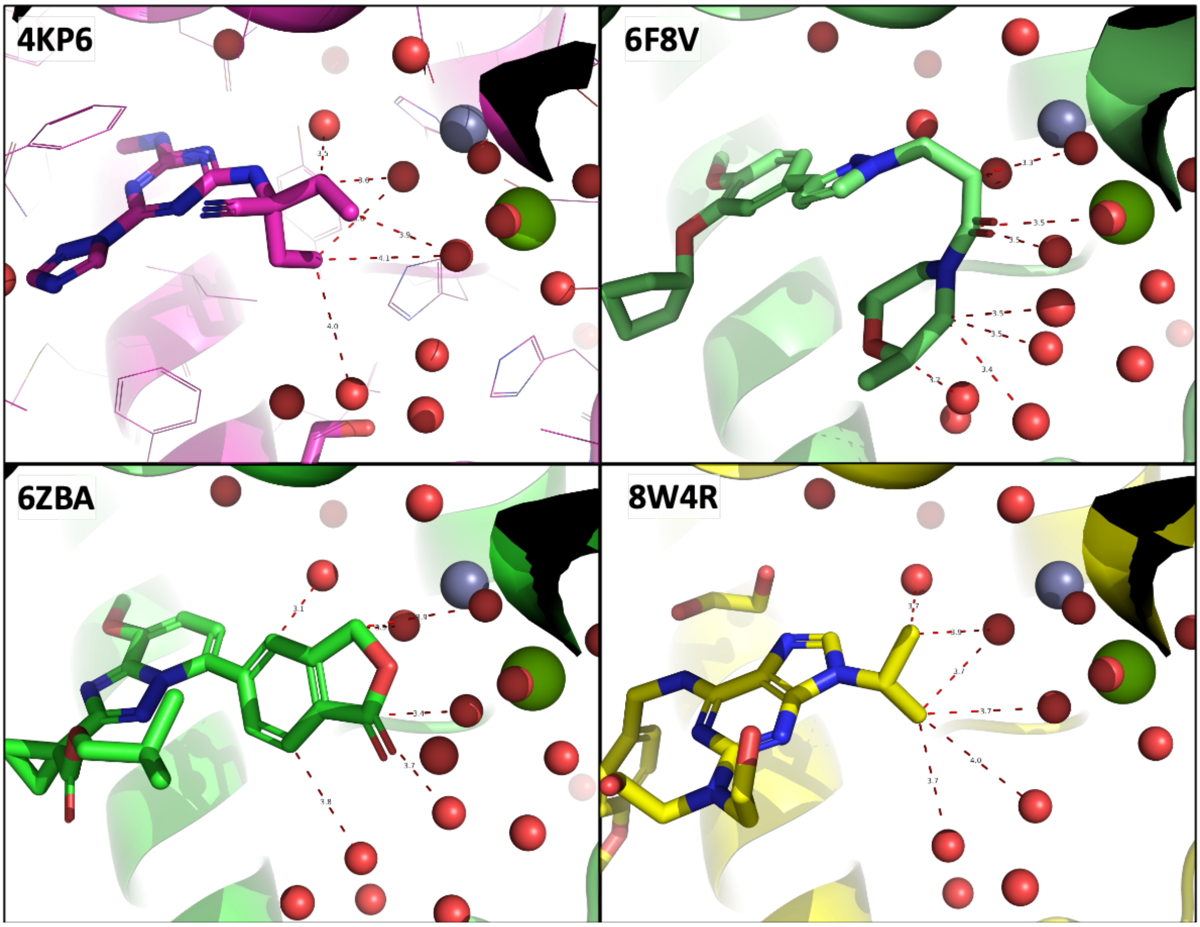

Figure S9: Examples of diverse ligands that make close contacts with the PDE4 water network through hydrophobic moieties (red dashed lines). PDB codes are indicated on the top-left corner of each image. Citations: [10–13].

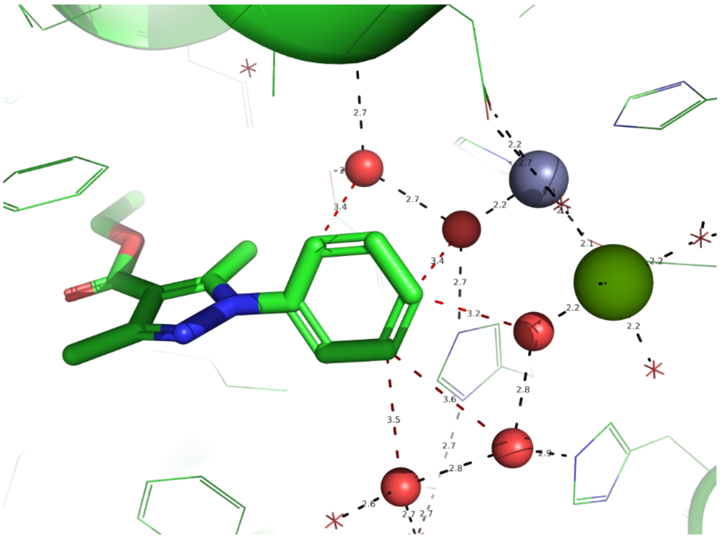

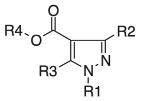

R1	R2	R3	R4	IC_{50} (μM; PDE4B)	IC_{50} (μM; PDE4D)
H-	CH_3-	CH_3-	H-	>200	>200
H-	CH_3-	CH_3-	CH_3-CH_2-	60	82
H-	H-	CH_3-	CH_3-CH_2-	>200	>200
H-	H-	NH_2-	CH_3-CH_2-	>200	>200
H-	H-	CF_3-	CH_3-CH_2-	>200	>200
H-	CH_3-	MXZ-	CH_3-CH_2-	>200	>200
H-	CH_3-	4-CH_3-Ph-	CH_3-CH_2-	15	19
Ph-	CH_3-	CH_3-	CH_3-CH_2-	0.31	0.27
Ph-	4-Cl-Ph-	H-	CH_3-CH_2-	1.5	0.88
QLY-	CH_3-	CH_3-	CH_3-CH_2-	17	19
BTP-	H-	NH_2-	CH_3-CH_2-	25	50

IC_{50} values are in μM.

Figure S10. Putative case of favourable hydrophobic contacts with a water network: PDE4D (PDB 1Y2C). Black lines indicate hydrogen bonds. Red lines indicate close contacts between an apolar atom of the ligand and water molecules in the network. IC_{50} values (left) are taken from [14] (main text reference 35).

Supplementary Tables

Table S1. Structural refinement details

	compound **4** (x603)	compound **2** (x606)
**PDB ID**	9QOB	9QNV
**Beamline**	DLS I04-1	DLS I04-1
**Data Collection**		
**Wavelength (Å)**	0.91587	0.91587
**Space Group**	$P2_12_12_1$	$P2_12_12_1$
**Cell dimensions**		
**a, b, c (Å)**	31.96, 47.09, 79.00	37.26, 43.86, 77.89
**α, β, ɣ (degrees)**	90.0, 90.0, 90.0	90.0, 90.0, 90.0
**Resolution range (Å)**	40.45 - 1.37 (1.393 - 1.370)	38.94 - 1.23 (1.29 - 1.23)
**No. measured intensities**	142865	197462
**No. unique reflections**	23965	37562
**Multiplicity**	6.0 (4.4)	5.3 (2.6)
**Mean I/σ(I)**	17.55 (3.73)	20.5 (4.4)
**Completeness (%)**	92.7 (84.3)	98.3 (88.7)
**Rmerge**	0.058 (0.319)	0.039 (0.186)
**CC1⁄2**	0.999 (0.902)	0.999 (0.933)
**Wilson B value (Å^2)**	10.2	7.0
**Refinement**		
**Software**	BUSTER	REFMAC
**Resolution range (Å)**	40.5 - 1.370	38.94 - 1.23
**Reflections: working/free**		
**Rwork (%)**	0.1698	0.11720
**Rfree (%)**	0.1962	0.14820
**No. Atoms**		
**Protein**	1090	1091
**Water**	110	216
**Ligands**	24	32
**B-factors**		
**Protein**	11.5	8.0
**Water**	22.7	23.7
**Ligand**	17.6	14.0
**R.M.S. deviations**		
**Bond lengths (Å)**	0.012	0.013
**Bond angles (°)**	1.12	1.79
**Ramachandran plot (%)**		
**favoured**	100	98.4
**outliers**	0	0

Table S2. Hydration sites (left) and their thermodynamic properties (table below). All values in kcal/mol and relative to bulk water. Observed ΔG_{bind} is actual free energy, derived from the MD-observed water densities (relative to bulk) using the Boltzmann relationship. For GIST, the first three terms are enthalpic contributions, which are added in the *SUM* column. The *Wat-Wat* term is expressed in relationship to the value in bulk solvent (i.e., -9.533 kcal/mol). GIST ΔG adds the enthalpic and entropic contributions, and captures the free energy of moving a water molecule from bulk to that particular location. Note that GIST ΔG reflects the intrinsic preferences of the water molecule, while *Observed ΔG* is the end product of all contributions by the entire system.

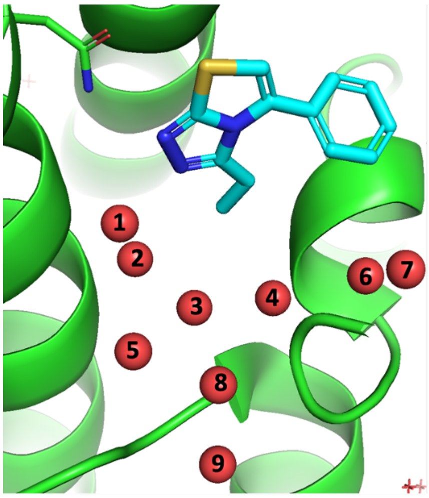

		Observed ΔG_{bind}	GIST ΔH_{bind} Wat-Prot	Wat-Lig	Wat-Wat*	SUM	GIST -ΔTS	GIST ΔG
**Unbound**	W1	-1.0	-10.1	0.0	4.6	-5.5	2.7	-2.8
	W2	-1.5	-9.3	0.0	3.5	-5.8	3.8	-2.0
	W3	-1.4	-8.1	0.0	2.7	-5.3	3.8	-1.6
	W4	-1.5	-12.3	0.0	5.7	-6.5	3.5	-3.1
	W5	-1.4	-14.6	0.0	6.7	-8.0	3.0	-4.9
	W6	-0.3	-12.9	0.0	6.0	-6.8	1.5	-5.3
	W7	0.1	-12.6	0.0	5.9	-6.7	0.5	-6.2
	W8	-0.4	-10.0	0.0	4.9	-5.2	1.7	-3.4
	W9	-0.8	-13.3	0.0	5.8	-7.4	1.8	-5.7
	SUM_ALL	-8.2	-103.2	0.0	45.9	-57.3	22.2	-35.1
	SUM_2,3,4	-4.4	-29.7	0.0	12.0	-17.7	11.0	-6.7
**1**	W1	-1.4	-9.5	-4.8	6.7	-7.7	3.9	-3.8
	W2	-1.8	-10.6	1.5	3.8	-5.3	4.4	-0.9
	W3	-1.6	-8.8	0.5	2.6	-5.7	4.2	-1.5
	W4	-1.8	-13.1	0.3	6.3	-6.5	4.2	-2.3
	W5	-1.7	-14.6	-0.4	6.9	-8.2	3.5	-4.6
	W6	-1.3	-14.5	-1.2	7.8	-7.9	3.2	-4.7
	W7	-0.9	-9.7	-0.1	5.1	-4.7	1.3	-3.4
	W8	-1.0	-8.2	-0.2	3.9	-4.6	2.6	-1.9
	W9	-1.3	-13.4	0.1	5.5	-7.8	2.0	-5.8
	SUM_ALL	-12.8	-102.4	-4.4	48.5	-58.4	29.4	-29.0
	SUM_2,3,4	-5.2	-32.4	2.3	12.6	-17.5	12.8	-4.7
**2**	W1	-1.6	-9.6	-5.2	6.6	-8.2	4.2	-4.0
	W2	-1.7	-10.6	1.7	3.6	-5.3	4.3	-1.0
	W3	-1.6	-8.7	0.7	2.5	-5.5	4.1	-1.4
	W4	-1.7	-12.9	0.2	5.9	-6.7	4.2	-2.6
	W5	-1.7	-15.1	-0.3	7.0	-8.4	3.6	-4.7
	W6	-1.0	-14.6	-1.1	7.8	-7.8	2.7	-5.1
	W7	-0.8	-9.0	0.0	5.0	-4.1	1.3	-2.8
	W8	-1.1	-8.6	-0.2	4.5	-4.4	2.6	-1.8
	W9	-1.3	-13.1	0.1	5.7	-7.4	1.8	-5.6
	SUM_ALL	-12.5	-102.1	-4.2	48.5	-57.7	28.8	-28.9

	SUM_2,3,4	-5.0	-32.1	2.6	11.9	-17.6	12.6	-4.9
	W1	-1.5	-9.4	-3.6	6.6	-6.4	3.6	-2.8
	W2	-1.6	-10.1	1.0	3.7	-5.4	4.1	-1.3
	W3	-1.5	-8.4	0.2	2.7	-5.5	3.9	-1.6
	W4	-1.6	-12.4	0.1	6.1	-6.2	3.8	-2.4
	W5	-1.5	-14.8	-0.3	6.9	-8.2	3.3	-4.9
**3**	W6	-1.3	-14.7	-1.0	7.5	-8.2	3.1	-5.1
	W7	-0.9	-8.8	0.0	4.5	-4.4	1.5	-2.9
	W8	-0.9	-7.9	-0.1	4.0	-4.0	2.4	-1.6
	W9	-1.3	-13.0	0.1	5.6	-7.4	1.8	-5.6
	SUM_ALL	-12.1	-99.6	-3.7	47.5	-55.8	27.5	-28.3
	SUM_2,3,4	-4.7	-30.9	1.3	12.6	-17.1	11.7	-5.3
	W1	-1.5	-9.4	-4.4	6.6	-7.2	3.8	-3.4
	W2	-1.5	-10.3	0.8	3.8	-5.7	4.0	-1.7
	W3	-1.4	-8.6	-0.1	2.9	-5.8	3.8	-2.0
	W4	-1.5	-12.2	-0.9	5.7	-7.3	3.8	-3.6
	W5	-1.6	-15.2	-0.5	7.2	-8.4	3.4	-5.0
**4**	W6	-1.0	-15.0	-1.3	7.9	-8.4	2.9	-5.6
	W7	-0.8	-9.3	-0.1	4.9	-4.4	1.4	-3.0
	W8	-0.8	-8.1	-0.1	4.1	-4.1	2.3	-1.8
	W9	-1.2	-13.4	0.1	5.9	-7.3	1.6	-5.7
	SUM_ALL	-11.2	-101.4	-6.4	49.0	-58.7	26.9	-31.8
	SUM_2,3,4	-4.4	-31.0	-0.1	12.4	-18.8	11.6	-7.2
	W1	-1.6	-9.7	-4.8	6.7	-7.8	4.1	-3.8
	W2	-1.7	-10.0	0.1	3.8	-6.1	4.1	-2.0
	W3	-1.5	-8.5	-0.5	2.8	-6.3	4.0	-2.3
	W4	-1.6	-12.5	-0.3	5.7	-7.0	3.7	-3.3
	W5	-1.6	-15.0	-0.4	7.0	-8.4	3.5	-4.9
**5^0**	W6	-0.9	-14.0	-1.2	7.5	-7.7	2.6	-5.2
	W7	-0.8	-10.2	-0.1	5.5	-4.9	1.3	-3.5
	W8	-0.9	-8.2	-0.1	4.1	-4.2	2.4	-1.9
	W9	-1.3	-13.2	0.1	5.7	-7.4	1.9	-5.5
	SUM_ALL	-11.8	-101.5	-7.2	48.8	-59.8	27.5	-32.3
	SUM_2,3,4	-4.7	-31.1	-0.7	12.4	-19.4	11.8	-7.6
	W1	-0.9	-8.8	-3.4	6.7	-5.4	2.3	-3.1
	W2	-1.5	-6.5	-10.7	7.8	-9.4	3.3	-6.1
	W3	-1.0	-7.0	-8.0	6.7	-8.4	2.9	-5.5
	W4	-0.8	-7.9	-7.9	6.6	-9.2	2.6	-6.6
	W5	-0.9	-14.6	2.7	5.5	-6.3	2.4	-4.0
**5^+**	W6	0.2	-7.3	-3.3	5.7	-5.0	0.7	-4.3
	W7	-0.1	-8.5	-0.4	5.0	-4.0	0.5	-3.4
	W8	-0.4	-6.1	-2.8	2.8	-6.1	2.3	-3.8
	W9	-1.0	-12.7	-1.4	5.7	-8.3	2.2	-6.1
	SUM_ALL	-6.5	-79.5	-35.2	52.5	-62.2	19.3	-42.9
	SUM_2,3,4	-3.3	-21.5	-26.6	21.1	-27.0	8.8	-18.2

Table S3. Water network in the unbound state (top) and thermodynamic analysis (bottom) of five hydration sites (named A to E) displaced by the ligand in the first and second layers around the investigated water network. The contour shows a water density equivalent to bulk solvent (1g, ΔG_{bind} = 0 kcal/mol), and reveals a low water density. Hydration sites B and C, which will be replaced by the aliphatic part of the ligand, do not form meaningful interactions with the investigated water network and are isolated from bulk solvent by a very low water density region. The position of ligand **1** and Asn140 (pink) are taken from the bound simulation and shown only for reference.

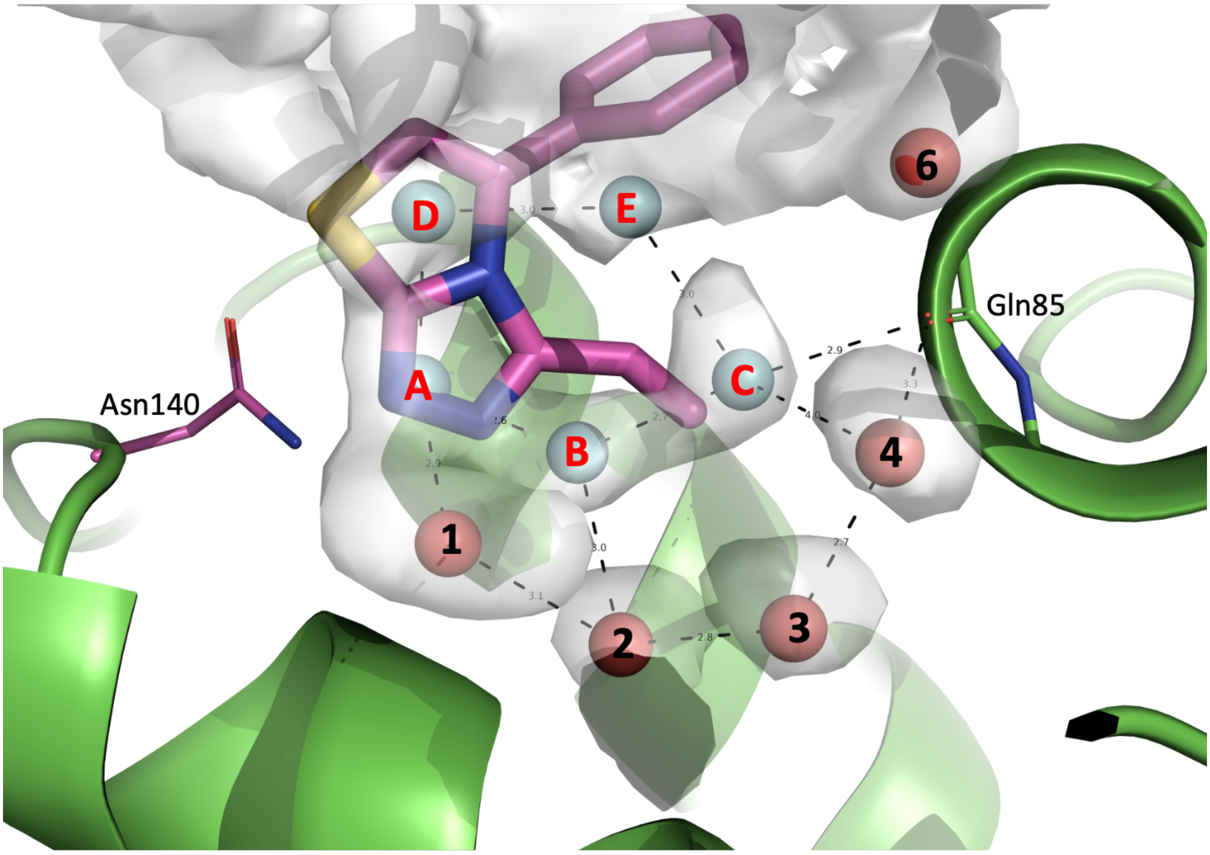

		Observed ΔG_{bind}	GIST ΔH_{bind}				GIST -ΔTS	GIST ΔG
			Wat-Prot	Wat-Lig	Wat-Wat*	SUM		
**Unbound**	W_A	-1.0	-6.3	0.0	2.3	-4.0	2.1	-1.8
	W_B	-0.8	-4.6	0.0	2.1	-2.5	2.2	-0.2
	W_C	-0.4	-7.2	0.0	4.9	-2.2	1.5	-0.8
	W_D	-0.4	-5.0	0.0	2.4	-2.6	0.9	-1.7
	W_E	-0.1	-2.7	0.0	1.5	-1.3	0.6	-0.7

Putative hydrophobic water networks in BACE1

The β-Site Amyloid Cleaving Enzyme 1 (BACE1) is an exceptionally well-studied system, Both the S1' and the S2' sites recognize small hydrophobic amino acids, and both present a water network surrounding the aliphatic side-chains (**Figure S10** and **Figure S11**, respectively). Some of the properties observed in those sites are reminiscent of what we have observed in BRD4. First, the tolerance for imperfect packing, which can explain that both Val and Ala are tolerated in S2' (**Figure S12**). Second, order to disorder transition, mediated by the formation of a more robust water network, which is particularly noticeable on site S1', where the water network connects to the flap (**Figure S10**). On this site, various series of compounds place a methyl group in direct contact with the water network,[15–17] whereas demethylation or bigger aliphatic groups appear to be disfavoured (**Figure S13**). MD and GIST analysis confirm that the water network becomes less stable in the absence of the ligand (**Figure S14**).

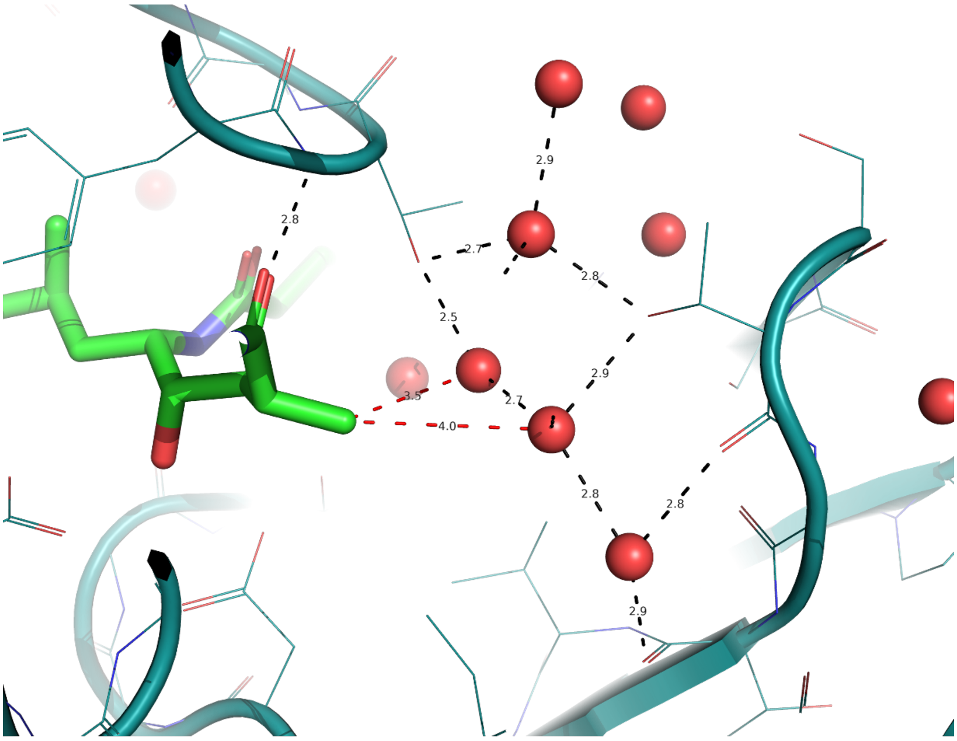

Figure S11: BACE1 Site S1'. PDB 2G94.[18] The water network connects to the flap (loop above the substrate, which can adopt an open or closed conformation) through Thr72.

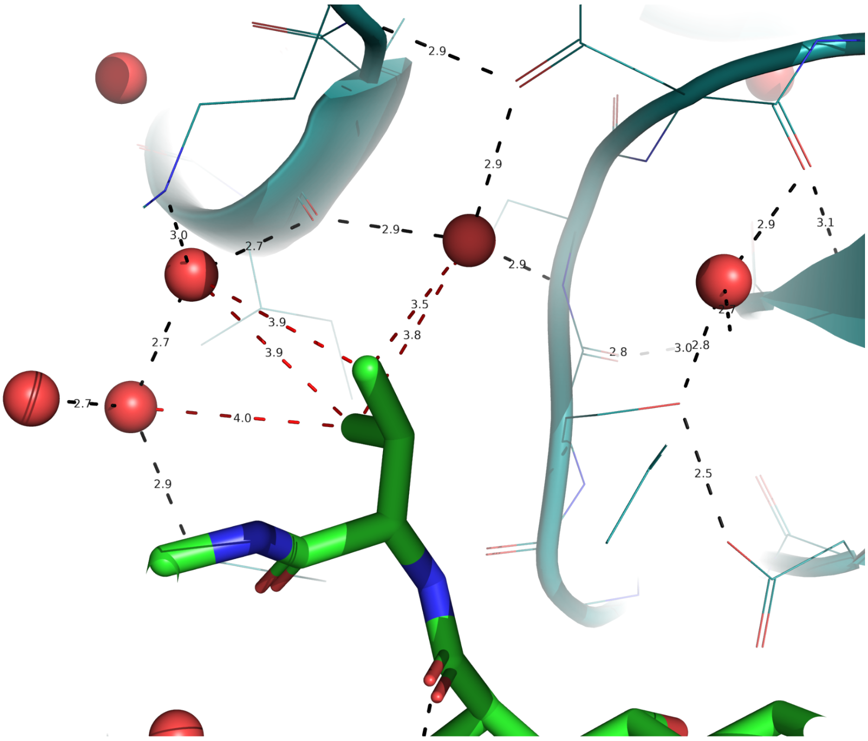

Figure S12: BACE1 Site S2’. PDB 2G94.[18]

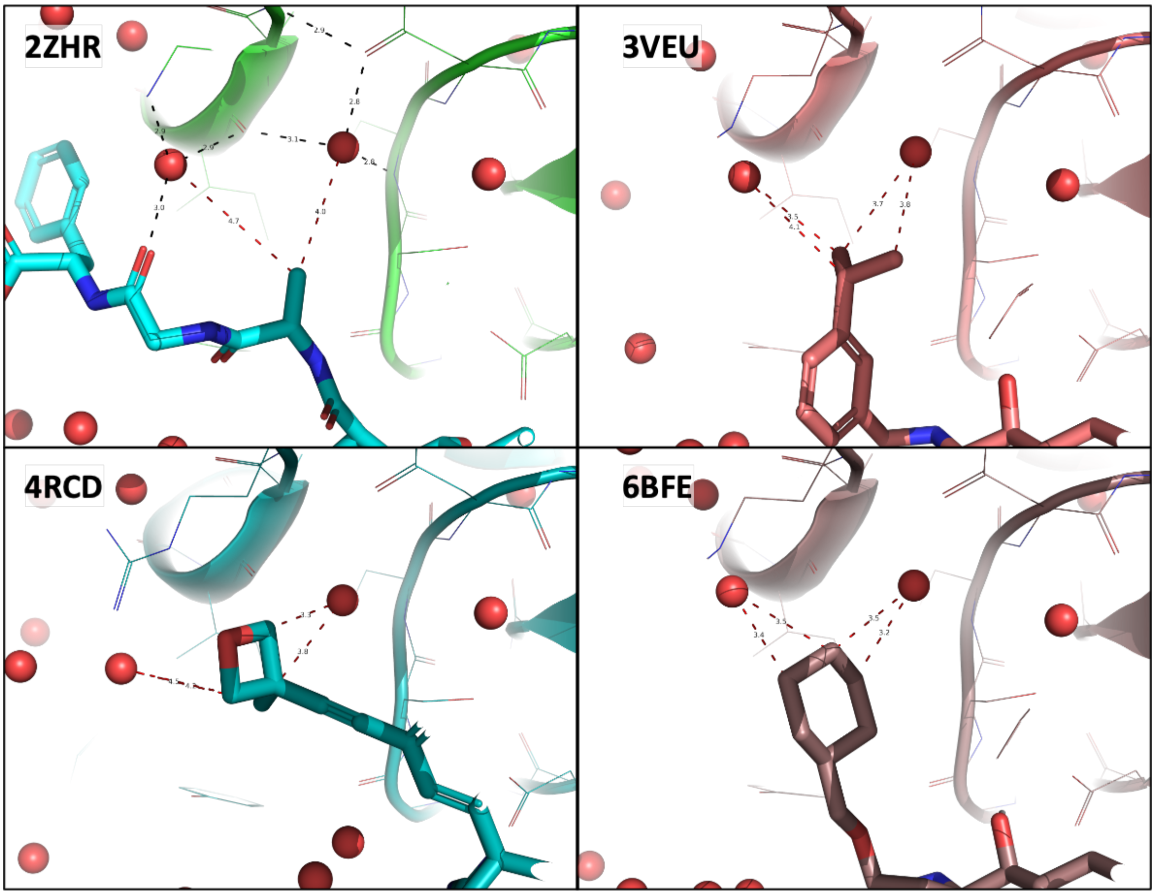

Figure S13: Examples of diverse ligands binding to Site S2’. Structure 2ZHR shows defective packing of the methyl with the water network. PDB codes are indicated on the top-left corner of each image. Citations: [19–22].

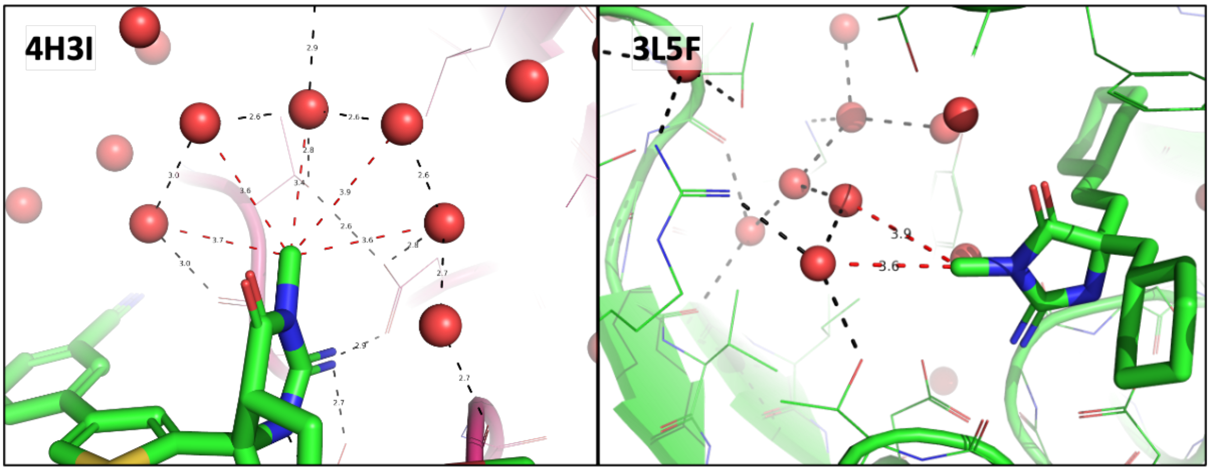

Figure S14: Examples of ligands forming close contacts with water molecules through a methyl group in the S1′ site of BACE1. PDB codes are indicated on the top-left corner of each image. Citations: [23,24].

Methods

Protein Production

The BRD4(1) construct was a kind donation of Dr. Alessio Ciulli (University of Dundee, UK) and it encodes residues 44-168. The cloned his-tagged BRD4(1) was expressed in Escherichia coli BL21 (DE3). For biophysical assays, the protein purification procedure consisted of immobilised metal ion affinity chromatography (IMAC), followed by TEV cleavage of the histidine tag and a second IMAC chromatography. For crystallisation assays, the purification protocol involved IMAC chromatography, followed by TEV cleavage and size-exclusion chromatography for crystallography. The expression and purification has already been described.[25]

Binding assay

The Time-Resolved Fluorescence Resonance Energy Transfer (TR-FRET) binding assay used to measure the disruption of the interaction of BRD4(1) with [Lys(Ac)5/8/12/16]-Histone H4 (1-21)-GGK(Biotin) (Eurogentec) with compounds **1**-**5**, was performed according to a previously described protocol.[26] The experiments were performed in 384-well black plates (NUNC) in a buffer composed of 50 mM HEPES, pH 7.4, 50 mM NaCl, 400 mM KF (Sigma), 0.5 mM CHAPS (Sigma), and 0.05% BSA (Sigma) in a final volume of 100 µL. BRD4(1) (100 nM) protein domain was mixed with 200 nM of the biotin-labelled histone peptides and incubated for 30 min at room temperature. Molecules were prepared in 250 mM HEPES, pH 7.4, 250 mM NaCl, and 20% DMSO at a single concentration to give a final concentration of 50 µM 1% DMSO in a total well volume of 100 µL and incubated for 1 h. Molecules showing inhibition were then screened in duplicate at several serial dilutions. Inhibition (%) at 50 µM was calculated with the following formula:

$$100 \times \left[1 - \frac{\text{competitor FRET ratio } \frac{665}{620} - \text{FRET ratio } \frac{665}{620} \text{ of the negative control}}{\text{FRET ratio } \frac{665}{620} \text{ of the positive control} - \text{FRET ratio } \frac{665}{620} \text{ of the negative control}}\right]$$

where the negative control was run without BRD4(1) and inhibitor and positive control, without inhibitor to give the bottom signal and top signal, respectively. The protein−peptide complexes at equilibrium were then detected with 2 nM Eu3+ cryptate-conjugated streptavidin (CisBio) and 10 nM anti-his6-XL665 (CisBio). Titration of a known inhibitor ((+)-JQ1) was used as the control for each experiment. IC50 values were calculated by plotting the log [competitor/inhibitor] versus the mean normalised response data from a single experiment measured in duplicate and fitting it to a four-parameter equation with variable Hill slope, using the GraphPad Prism software:

$$Y = \left\{\text{bottom} + \frac{\text{top} - \text{bottom}}{[1 + 10(\log \text{IC}_{50}x)]} \times \text{Hill slope}\right\}$$

The plates were measured in a CLARIOStar reader (BMG Labtech) using the homogeneous time-resolved fluorescence module (excitation, 337 nm with 200 flashes; emission, 620 and 665 nm). The 665 nm/620 nm ratios were converted to % normalised TR-FRET ratio = top ratio signal as 100% and

bottom ratio signal as 0% to normalise experiments performed at different days. Dose–response curves are shown in **Figure S3**.

Structural Determination

<u>1. Crystallisation.</u>

Aliquots of the mixture of BRD4(1) and the respective compound were set up for crystallisation using a mosquito crystallisation robot (TTP Labtech) by performing three different drop ratios of precipitant to protein per condition (200 + 100 nL, 150 + 150 nL, and 100 + 200 nL). All crystallizations were carried out using the sitting drop vapour diffusion method at 4 °C. Crystals of BRD4(1) in complex with compound **4** were grown by mixing 100 nL of BRD4(1) (5 mg/mL) and compound **4** (10 mM) mixture with 200 nL of reservoir solution consisting of 0.2 M sodium chloride, 0.1 M MES pH 6, 30% v/v Jeffamine® ED-2003. Crystals of BRD4(1) in complex with compound **2** were grown by mixing 100 nL of BRD4(1) (5 mg/mL) and compound **2** (10 mM) mixture with 200 nL of reservoir solution consisting of 0.2 M potassium chloride, 0.1 M glycine pH 9.5, 20% v/v pentaerythritol.

<u>2. Data Collection and Structure Solution.</u>

X-ray diffraction data were collected on beamlines I03 and I04-1 at the Diamond Light Source. Data were integrated with XDS[27] and scaled with AIMLESS[28] as part of the XIA2 and autoPROC auto-processing pipelines.[29,30] Initial refinement and map calculation was carried out with DIMPLE[31] using a ligand-free version of BRD4(1) (PDB 3MXF[25]) as starting model. Ligand restraints were generated with ACEDRG[32] and GRADE.[33] Refinement and model building was performed with REFMAC[34] and COOT,[35] respectively. Structure validation was performed with MolProbity.[36] Final models and structure factors have been deposited with PDB accession codes 9QOB and 9QNV. Data collection and refinement statistics are summarized in **Table S1**.

Molecular Dynamics

<u>1) Protein and Ligand Preparation</u>

Ligand **1** was co-crystallized in the previous study[9] with PDB code 6ZF9. The ligand pose was extracted from the PDB and missing hydrogens were added with MOE.[37] Ligands **2**, **3**, **4**, **5^0** and **5^+** were prepared from this pose using the "Build" tool in MOE.[37] The coordinates were maintained, changing only the position of the particular substituent. This part was minimised with MOE and each ligand was saved as a MOL2 file. Gaussian[38] was used to optimize and generate ESP charges and potential at the HF/6-31G(d) level, and RESP[39] partial charges were derived using Antechamber.[40] Parameterization with the GAFF2 force field was applied.

The protein was extracted from the PDB and it was prepared with MOE ("Protein Preparation" tool).[37] All the crystallized waters were removed except for seven water molecules that were kept (301, 304, 316, 320, 322, 324, 331). The protein was saved as MOL2 with AMBER nomenclature.

Each ligand, the unbound protein and the seven protein-ligand complexes were neutralised and solvated in an octahedral box of TIP3P water with tleap.[40] Topology (prmtop) and coordinates (prmcrd) Amber files were obtained for each of them.

2) Unbiased molecular dynamics.

The same molecular dynamics steps were applied to the three cases (unbound, ligand, complex) with pmemd.cuda of Amber[40] and in three independent replicas:
1) Minimization. Two steps of energy minimization for 1,000 cycles each were performed. The default algorithm is used with 10 cycles of steepest descent method and then conjugate gradient is switched on. Energy information is printed every 100 steps. Atoms of the ligands and the protein are restrained using a harmonic potential with a force constant of 25 kcal/mol·Å2 in the first step and a force constant of 5 kcal/mol·Å2 in the second step.
2) Progressive heating in NVT. Atoms of the ligands and the protein are restrained using a harmonic potential with a force constant of 5 kcal/mol·Å2. The initial temperature was set to 100K and was increased by 50K in 4 steps (150K, 200K, 250K, 300K).
3) Equilibration in NPT. Atoms of the ligands and the protein are restrained using a harmonic potential with a force constant of 5 kcal/mol·$Å^2$. The temperature was set to 300K. Constant pressure periodic boundary conditions are used with isotropic position scaling with a Berendsen barostat for a pressure of 1.0 atm and a pressure relaxation time of 2.0 ps. In a first step 10,000 MD-steps are performed and in a second step 490,000 MD-steps are performed, both with a time step of 0.002 ps, using SHAKE.
4) Progressive release of the restraints in NVT. Atoms of the ligands and the protein are restrained using a harmonic potential with a force constant of 5, 4, 3, 2 & 1 kcal/mol·Å2 for the first, second, third, fourth & fifth simulation, respectively. The temperature was set to 300K. In steps 1, 2, 3 and 4 the restraints were applied to both the protein and the ligand in the case of complexes.
5) Equilibration in NVT without restraints. No restraints are applied. The temperature was set to 300K. Constant volume.
In step 2, 3, 4, and 5 the following conditions are used unless specifically stated otherwise: energy information and the coordinates are written every 2000 steps; for each simulation 100,000 MD-steps are performed with a time step of 0.002 ps, using SHAKE; In the NVT and NPT ensemble the Langevin thermostat was used with a collision frequency γ of 4.0 with a random seed generator
6) Production. 200 steps of 1 ns in NVT (for a total of 200 ns) were performed. Energy information and the coordinates are written every 5000 steps. 500,000 MD-steps are performed with a time step of 0.002 ps, using SHAKE. The Langevin thermostat was used with a collision frequency γ of 4.0 with a random seed generator. The temperature was set to 300K. For the ligands in water only 20ns were performed. Nonbonded cut-off was 8.0 Å for free ligand simulation and 9.0 Å for the simulation of unbound protein and protein-ligand complexes.

3) Free Energy Calculations.

Minimization, heating and equilibration were performed following the protocol described above. The restart file after the last equilibration step was used to prepare the prmtop and prmcrd files for the alchemical transformations.
For each transformation the “START” ligand coordinates were obtained from the restart file. The coordinates for the common atoms were used for the “END” ligand. The atoms of the different substituent attached to the heterocycle were considered unique. tleap added them in the end ligand from the OFF file. Prmtop and prmcrd with both ligands in the same file were obtained (needed for pmemd). The same procedure was applied for ligands and complexes (**Scheme S1**).

21 lambdas were used: 0.0, 0.02, 0.04, 0.06, 0.08, 0.10, 0.15, 0.20, 0.30, 0.40, 0.50, 0.60, 0.70, 0.80, 0.85, 0.90, 0.92, 0.94, 0.96, 0.98, 1.00. Each lambda is run in parallel. For each lambda, it is performed 1 step of equilibration of 1ns and 20 steps of production of 1ns each (total 20ns).
Energy information is printed every 4000 steps. The coordinates are written every 4000 steps. 1,000,000 MD-steps are performed with a time step of 0.001 ps, not using SHAKE. The Langevin thermostat was used with a collision frequency γ of 4.0 with a random seed generator. The temperature was set to 300K. NVT was used in the equilibration step while the production step was in the NPT ensemble. Constant pressure periodic boundary conditions are used in production with isotropic position scaling with a Monte Carlo barostat for a pressure of 1.0 atm and a pressure relaxation time of 2.0 ps. Non-bonded cut-off was 10.0 Å. timask1 was the starting ligand, timask2 the ending ligand. Softcore potential was applied to the unique atoms. ∂V/∂λ values and the Bennett acceptance ratio scheme were calculated at every step. For some transformation to avoid using unnecessary space, the coordinates are written every 10,000 steps. Amber18 was used with the patch update.16.[40]
Analysis was carried out with *Alchemical Analysis*, a program from the Mobley lab (https://github.com/MobleyLab/alchemical-analysis).[41]

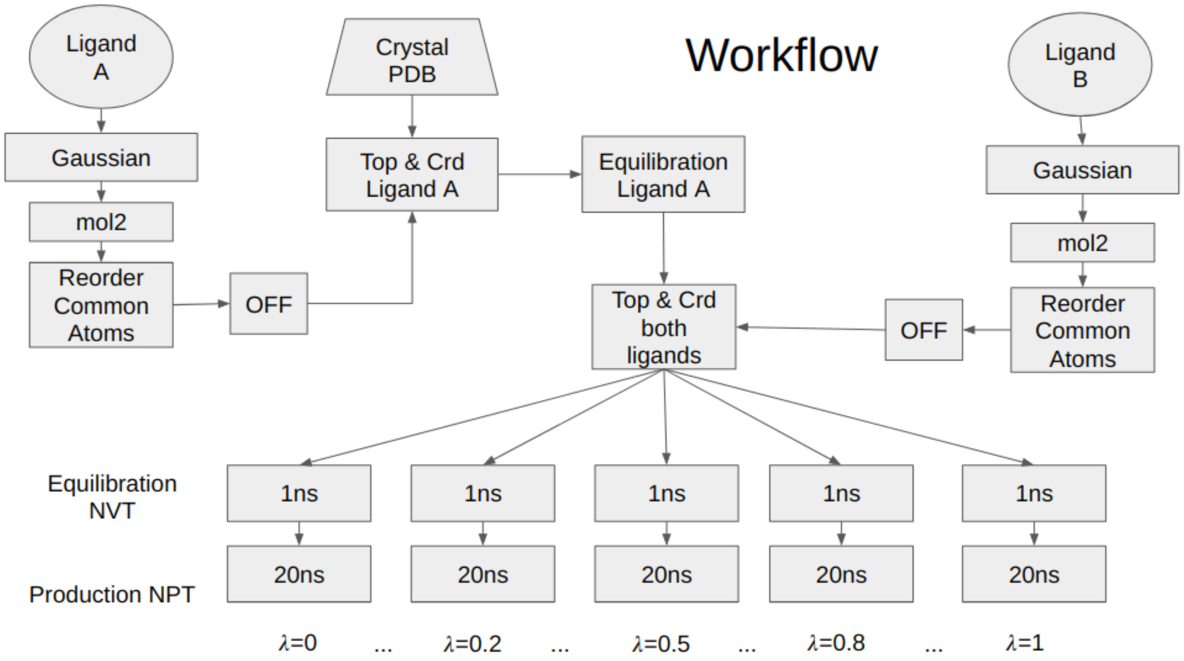

Scheme S1: Workflow used for the Alchemical Transformation to calculate the Relative Free Energies of Binding.

4) GIST Analysis

cpptraj,[42] part of the AmberTools package,[43] was used to analyse the 600 ns (3 replicas x 200 ns) generated for each system. The α-carbons of the residues lining the binding site (80, 81, 82, 83, 84, 85, 86, 87, 88, 92, 94, 97, 101, 105, 106, 107, 132, 133, 135, 136, 139, 140, 146, 147, 149, 150 & 153) were used to align each snapshot to the initial geometry. The centre of mass of the same atoms was placed at the origin of coordinates. All simulations resulted in stable trajectories, except for **5**$^+$, which

dissociated during the 3 replicas. Accordingly, for this ligand we used 160 ns from the beginning of the three replicas, where the binding mode remained stable. For all other systems, we used the entire 600 ns. Grid inhomogeneous solvation theory (GIST) analysis[44,45] was performed using the following parameters: refdens 0.0329 temp 300.0 excludeions gridcntr 0.0 0.0 0.0 griddim 28 28 28 gridspacn 0.50. To generate the reported values (Table 2 and Table S2), the position of each water molecule (taken from the initial PDB structure 6ZF9) was used as reference, the eight most-populated voxels (higher OW density) within 1.0A were selected, and their values were weight-averaged.

Note on the Accuracy of the Simulations:

Considering the simplicity of the protein force-field and the three-point water model used here (TIP3P), one must wonder about the ability of the simulations to reproduce the natural system. After completing the analyses, we must conclude that the level of theory is adequate, and the results also offer an explanation: the phenomenon described here is the result of how water molecules place and orient themselves in different environments. The electrostatic and Lennard-Jones potentials that force-fields use to mimic non-bonded interactions (including hydrogen bonds) are sufficient to identify the most favourable positions and orientations of water molecules in response to different environments. However, second order effects such as polarization or small changes in geometry (note that the H-O distance and the H-O-H angle is fixed in the TIP3P water model), will have an effect that prevent the calculations from being completely accurate. We want to emphasize that the simulations are instrumental to understand the phenomenon, but we do not expect (nor seek) quantitative predictions, as they would require a higher level of theory that explicitly considers geometric and electronic reorganization of individual water molecules in their respective environments.

Synthesis

General information

Synthesis of **1** has been described in 1 where it was named compound **23.** Compounds **2**-**5** were synthesized using the same general procedure, detailed below.

Reagents, solvents, and starting products were acquired from commercial sources. Evaporation of solvents was accomplished with a rotary evaporator. The term "concentration" refers to vacuum evaporation using a Büchi rotavapor. When indicated, the reaction products were purified by flash chromatography on silica gel (35−70 µm) with the indicated solvent system. The melting points were measured in a MFB 59510M Gallekamp instrument. IR spectra were performed in a spectrophotometer Spectrum Two FT-IR Spectrometer, and only noteworthy IR absorptions (cm^{-1}) are listed. NMR spectra were recorded in $CDCl_3$ at 400 MHz (^1H) and chemical shifts are reported in δ values downfield from TMS or relative to residual $CDCl_3$ (7.26 ppm, 77.00 ppm), DMSO-d_6 (2.50 ppm, 39.52 ppm) or CD_3OD (3.31 ppm, 49.00 ppm) as an internal standard. Data are reported in the following manner: chemical shift, multiplicity, coupling constant (*J*) in hertz (Hz) and integrated intensity. Multiplicities are reported using the following abbreviations: s, singlet; d, doublet; dd, doublet of doublets; dm, doublet of multiplets; m, multiplet; and bs, broad signal. Evaporation of solvents was accomplished with a rotary evaporator. The accurate mass analyses were carried out using a LC/MSD-TOF spectrophotometer. HPLC-MS (Agilent 1260 Infinity II) analysis was conducted on a Poroshell 120 EC-C15 (4.6 mm x50 mm, 2.7 µm) at 40 ºC. Mobile phase (A: H_20 + 0.05% formic acid and B: acetonitrile + 0.05% formic acid) using a gradient elution. Flow rate 0.6 mL/min. The DAD detector was set at 254 nm and the injection volume was 5 µL and oven temperature 40 °C. All tested compounds possess a purity of at least 95% (HPLC-MS).

2-Chloro-*N*'-(4-phenylthiazol-2-yl)acetohydrazide (PreSSR-1)

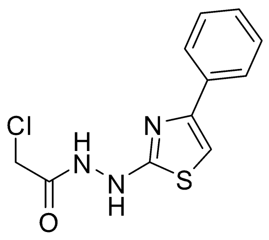

To a cooled solution (0 °C) of 2-hydrazinyl-4-phenylthiazole (1.50 g, 7.85 mmol) in THF (20 mL) was added dropwise a solution of chloroacetyl chloride (0.70 mL, 8.87 mmol) in THF (7 mL). The reaction mixture was stirred at 0 °C for 30 minutes and the precipitate formed was collected by filtration and washed with water to give **PreSSR-1** (1.95 g, 93%) as a white solid. ^{1}H-NMR Spectral data are coincident with the previously reported.26

IR (ATR): 3163, 2969, 2737, 1723, 1624, 1537, 1485, 1397, 1339, 908, 799, 741, 683 cm^{-1}; ^{1}H NMR (400 MHz, DMSO-d$_6$) δ 4.22 (s, 2H, C*H*$_2$), 7.28 (s, 1H, SC*H*), 7.29-7.33 (m, 1H, Ar*H*), 7.38-7.42 (m, 2H Ar*H*), 7.80-7.82 (dd, *J* = 8.0 Hz, 2H, Ar*H*), 9.17 (bs, 1H, N*H*CN), 10.92 (bs, 1H, N*H*CO); ^{13}C NMR (400 MHz, DMSO-d$_6$) δ 41.0 (*C*H$_2$), 103.6 (S*C*H), 125.8 (2*C*HAr), 128.0 (*C*HAr), 128.7 (2CHAr), 133.5 (*C-ipso*), 148.7 (N*C*CH), 166.2 (*C*O), 171.8 (N*C*S); HRMS $C_{11}H_{11}ClN_3OS$ [M+H]$^+$ 268.0306; found, 268.0307.

3-(Chloromethyl)-5-phenylthiazolo[2,3-*c*][1,2,4]triazole (SSR-1)

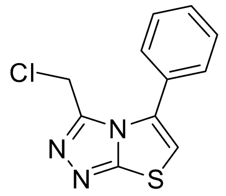

A mixture of **PreSSR-1** (1.22 g, 4.57 mmol) in xylene (10 mL) and phosphoryl chloride (7.65 mL, 82.07 mmol) was heated at 110 °C for 4 hours. Then, the reaction mixture was evaporated and neutralized with saturated $NaHCO_3$ solution. The aqueous phase was extracted with EtOAc, the combined organic phases were washed with brine, dried over Na_2SO_4, and evaporated to give a residue, which was purified by flash column chromatography (EtOAc) to give **SSR-1** as a yellowish solid (824 mg, 72%).

IR (ATR): 3164, 3111, 2924, 1724, 1603, 1486, 1194, 1095, 1001, 802, 756, 730, 684 cm^{-1}; ^{1}H NMR (400 MHz, $CDCl_3$) δ 4.42 (s, 2H, C*H*$_2$), 6.89 (s, 1H, SC*H*), 7.47-7.53 (m, 3H Ar*H*), 7.73-7.75 (dm, *J* = 8.2 Hz, 2H, Ar*H*); ^{13}C NMR (400 MHz, HETCOR, $CDCl_3$) δ 46.6 (*C*H$_2$), 103.8 (S*C*H), 107.0 (C-*ipso*), 126.0 (2*C*HAr), 128.8 (*C*HAr), 129.3 (2*C*HAr), 129.5, 147.2, 167.8 (N*C*N, N*C*CH, N*C*S); HRMS $C_{11}H_9ClN_3S$ [M+H]$^+$ 250.0200; found, 250.0205.

3-(Hydroxymethyl)-5-phenylthiazolo[2,3-*c*][1,2,4]triazole (SSR-2; compound **4** in main text**)**

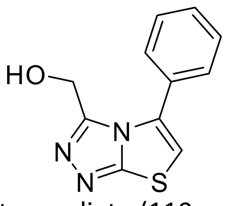

A mixture of **SSR-1** (400 mg, 1.60 mmol), sodium acetate (400 mg, 4.88 mmol), sodium iodide (16 mg, 0.11 mmol) and DMF (6 mL) was heated at 100 °C for 2 hours. The solvent was removed under reduced pressure and the residue was partitioned between EtOAc and water. The organic phase was washed with brine, dried over Na_2SO_4, and evaporated to give the corresponding acetoxy intermediate (240 mg,) as a brown solid. Then, a mixture of acetoxy intermediate (110 mg), MeOH (15 mL) and a solution of NaOH 3N (1 mL) was heated at reflux for 15 minutes. The MeOH was evaporated and the resulting aqueous solution was neutralized with saturated $NaHCO_3$ solution. The aqueous phase was extracted with EtOAc, the combined organic phases were washed with brine, dried over Na_2SO_4, and evaporated to give a residue, which was purified by column chromatography (1:0 to 97:3 EtOAc:MeOH) to give **SSR-2** as a yellow solid (32 mg, 34%).[46]

IR (NaCl): 3263, 3105, 2924, 2854, 1704, 1538, 1469, 1442, 1295, 1017, 775, 693 cm^{-1}; ^{1}H NMR (400 MHz, $CDCl_3$) δ 4.61 (s, 2H, C*H*$_2$), 6.80 (s, 1H, SC*H*), 7.53-7.57 (m, 3H Ar*H*), 7.58-7.61 (dd, *J* = 7.4 Hz, 2H, Ar*H*); ^{13}C NMR (400 MHz, $CDCl_3$) δ 54.7 (*C*H$_2$), 114.2 (SCH), 128.2 (*C-ipso*), 129.2 (2*C*HAr), 129.3 (2*C*HAr),

130.7 (*C*HAr), 131.6, 148.4, 158.6 (N*C*N, N*C*CH, N*C*S); HRMS $C_{11}H_{10}N_3OS$ $[M+H]^+$ 232.0539; found, 232.0534. Purity 95.7% (t_R= 3.43 min).

_N'_-(4-Phenylthiazol-2-yl)acetohydrazide (PreSSR-3)

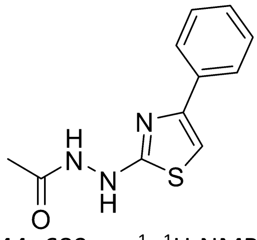

To a cooled solution (0 °C) of 2-hydrazinyl-4-phenylthiazole (300 mg, 1.57 mmol) in THF (5 mL) was added dropwise a solution of acetyl chloride (0.13 mL, 1.82 mmol) in THF (3 mL). The reaction mixture was stirred at 0 °C for 30 minutes and the precipitate formed was collected by filtration and washed with water to give **PreSSR-3** (351 mg, 96%) as a white solid.

IR (ATR): 3365, 3108, 2949, 2835, 1687, 1616, 1499, 1285, 1171, 992, 903, 744, 689 cm^{-1}; ^{1}H NMR (400 MHz, DMSO-d$_6$) δ 1.91 (s, 3H, C*H*$_3$), 7.23 (s, 1H, SC*H*), 7.26-7.31 (m, 1H, Ar*H*), 7.37-7.40 (m, 2H, Ar*H*), 7.80-7.83 (dd, *J* = 9.2 Hz, 2H, Ar*H*), 9.42 (bs, 1H, N*H*CN), 10.16 (bs, 1H, N*H*CO); ^{13}C NMR (400 MHz, DMSO-d$_6$) δ 20.6 (*C*H$_3$), 103.3 (S*C*H), 125.9 (2*C*HAr), 128.1 (*C*HAr), 128.7 (2*C*HAr), 133.0 (*C-ipso*), 147.8 (N*C*CH), 169.3 (*C*O), 172.8 (N*C*S); HRMS $C_{11}H_{12}N_3OS$ $[M+H]^+$ 234.0696; found, 234.0699.

3-Methyl-5-phenylthiazolo[2,3-*c*][1,2,4]triazole (SSR-3; compound **2** in main text**)**

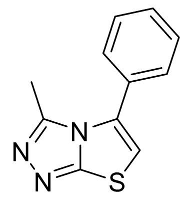

A mixture of **PreSSR-3** (115 mg, 0.49 mmol) in xylene (2 mL) and phosphoryl chloride (1.65 mL, 17.7 mmol) was heated at 110 °C for 4 hours. Then, the reaction mixture was evaporated and neutralized with saturated $NaHCO_3$ solution. The aqueous phase was extracted with EtOAc, the combined organic phases were washed with brine, dried over Na_2SO_4, and evaporated to give a residue, which was purified by column chromatography (EtOAc) to give **SSR-3** as a yellowish solid (51 mg, 48%).

IR (ATR): 3028, 2928, 1716, 1492, 1473, 1434, 1378, 1031, 762, 717, 700, 666 cm^{-1}; ^{1}H NMR (400 MHz, $CDCl_3$) δ 2.23 (s, 3H, C*H*$_3$), 6.72 (s, 1H, SC*H*), 7.44-7.46 (dd, *J* = 8.0 Hz, 2H, Ar*H*), 7.49-7.54 (m, 3H Ar*H*); ^{13}C NMR (400 MHz, $CDCl_3$) δ 12.6 (*C*H$_3$), 113.7 (S*C*H), 128.2 (*C-ipso*), 128.9 (2*C*HAr), 129.6 (2*C*HAr), 130.5 (*C*HAr), 131.0, 144.9, 157.9 (N*C*CH, N*C*N, N*C*S); HRMS $C_{11}H_{10}N_3S$ $[M+H]^+$ 216.0590; found, 216.0588. Purity 98.9% (t_R= 3.72 min).

3-(Azidomethyl)-5-phenylthiazolo[2,3-*c*][1,2,4]triazole (SSR-N$_3$)

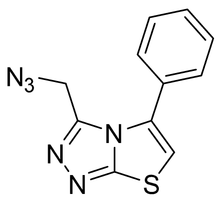

A mixture of **SSR-1** (400 mg, 1.60 mmol) and sodium azide (208 mg, 3.20 mmol) in DMF (8 mL) was heated at 50 °C for 2 hours. The reaction was quenched with water and was extracted with EtOAc. The combined organic phases were washed with brine, dried over Na_2SO_4, and evaporated to give a residue, which was purified by column chromatography (1:1 of hexane:EtOAc) to give **SSR-N$_3$** (203 mg, 46%) as a beige solid.[47]

IR (ATR): 3034, 2856, 2095, 1471, 1317, 1263, 1197, 1035, 814, 764, 702 cm^{-1}; ^{1}H NMR (400 MHz, $CDCl_3$) δ 4.36 (s, 2H, C*H*$_2$), 6.83 (s, 1H, SC*H*), 7.54-7.57 (m, 5H Ar*H*); HRMS $C_{11}H_9N_6S$ $[M+H]^+$ 257.0604; found, 257.0607.

3-(Aminomethyl)-5-phenylthiazolo[2,3-*c*][1,2,4]triazole (SSR-6; compound **5** in main text**)**

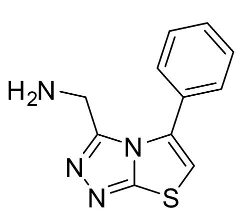

To a mixture of **SSR-N_3** (100 mg, 0.39 mmol) in THF (4 mL), triphenylphosphine (160 mg, 0.61 mmol) and water (28 µL 1.56 mmol) were added. The mixture was heated at 65 °C for 2 hours. Then, the reaction mixture was concentrated to give a residue, which was crystallized with acetonitrile to give **SSR-6** as a white solid (50 mg, 56%).

M.p. 82-84 °C (acetonitrile); IR (ATR): 2921, 2851, 1573, 1470, 1306, 1142, 1027, 760, 702 cm^{-1}; ^1H NMR (400 MHz, CD_3OD) δ 4.04 (s, 2H, C*H*$_2$), 7.29 (s, 1H, SC*H*), 7.61-7.63 (m, 3H Ar*H*), 7.65-7.68 (m, 2H, Ar*H*); HRMS $C_{11}H_{11}N_4S$ $[M+H]^+$ 231.0699; found, 231.0702. Purity 95.7% (t_R= 2.95 min).[48]

2-Methoxy-*N*'-(4-phenylthiazol-2-yl)acetohydrazide (PreSSR-7)

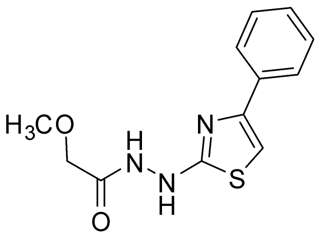

To a cooled solution (0 °C) of 2-hydrazinyl-4-phenylthiazole (300 mg, 1.57 mmol) in THF (5 mL) was added dropwise a solution of 2-methoxyacetyl chloride (0.16 mL, 1.75 mmol) in THF (2 mL). The reaction mixture was stirred at 0 °C for 30 minutes and the precipitate formed was collected by filtration and washed with water to give **PreSSR-7** (338 mg, 82%) as a white solid.

IR (ATR): 3122, 2873, 2772, 1612, 1518, 1414, 1230, 1180, 1002, 774, 692 cm^{-1}; ^1H NMR (400 MHz, $CDCl_3$) δ 3.37 (s, 3H, C*H*$_3$), 3.99 (s, 2H, C*H*$_2$), 7.27 (s, 1H, SC*H*), 7.30-7.33 (m, 1H Ar*H*), 7.39-7.42 (m, 2H Ar*H*), 7.80-7.82 (d, *J* = 7.6 Hz, 2H, Ar*H*), 10.04 (bs, 1H, N*H*CN), 10.52 (bs, 1H, N*H*CO); ^{13}C NMR (400 MHz, $CDCl_3$) δ 58.8 (*C*H$_3$O), 70.6 (*C*H$_2$), 103.4 (S*C*H), 125.8 (2*C*HAr), 128.0 (*C*HAr), 128.7 (2*C*HAr), 133.3 (*C-ipso*), 148.3, 169.2, 172.4 (N*C*CH, N*C*N, N*C*S); HRMS $C_{12}H_{14}N_3O_2S$ $[M+H]^+$ 264.0801; found, 264.0807.

3-(Methoxymethyl)-5-phenylthiazolo[2,3-*c*][1,2,4]triazole (SSR-7; compound **3** in main text**)**

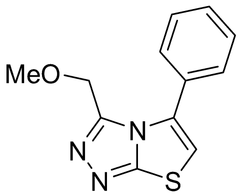

A mixture of **PreSSR-7** (100 mg, 0.38 mmol) in xylene (2 mL) and phosphoryl chloride (1.24 mL, 13.3 mmol) was heated at 110 °C for 4 hours. Then, the reaction mixture was evaporated and neutralized with saturated $NaHCO_3$ solution. The aqueous phase was extracted with EtOAc, the combined organic phases were washed with brine, dried over Na_2SO_4, and evaporated to give a residue, which was purified by flash column chromatography (EtOAc) to give **SSR-7** as a white solid (59 mg, 63%).

IR (ATR): 3094, 2973, 1612, 1599, 1518, 1225, 1176, 912, 712, 687 cm^{-1}; ^1H NMR (400 MHz, $CDCl_3$) δ 3.04 (s, 3H, CH_3), 4.38 (s, 2H, C*H*$_2$), 6.79 (s, 1H, SC*H*), 7.52-7.58 (m, 5H Ar*H*); ^{13}C NMR (400 MHz, $CDCl_3$) δ 57.9 (*C*H$_3$), 63.4 (*C*H$_2$), 114.2 (S*C*H), 128.5 (*C*-ipso), 128.8 (2*C*HAr), 129.3 (2*C*HAr), 130.4 (CHAr), 131.7 (N*C*CH), 145.3 (N*C*N), 159.0 (N*C*S); HRMS $C_{12}H_{12}N_3OS$ $[M+H]^+$ 246.0696; found, 246.0692. Purity 99.7% (t_R= 3.78 min).

^{1}H-NMR and ^{13}C-NMR spectra of new compounds

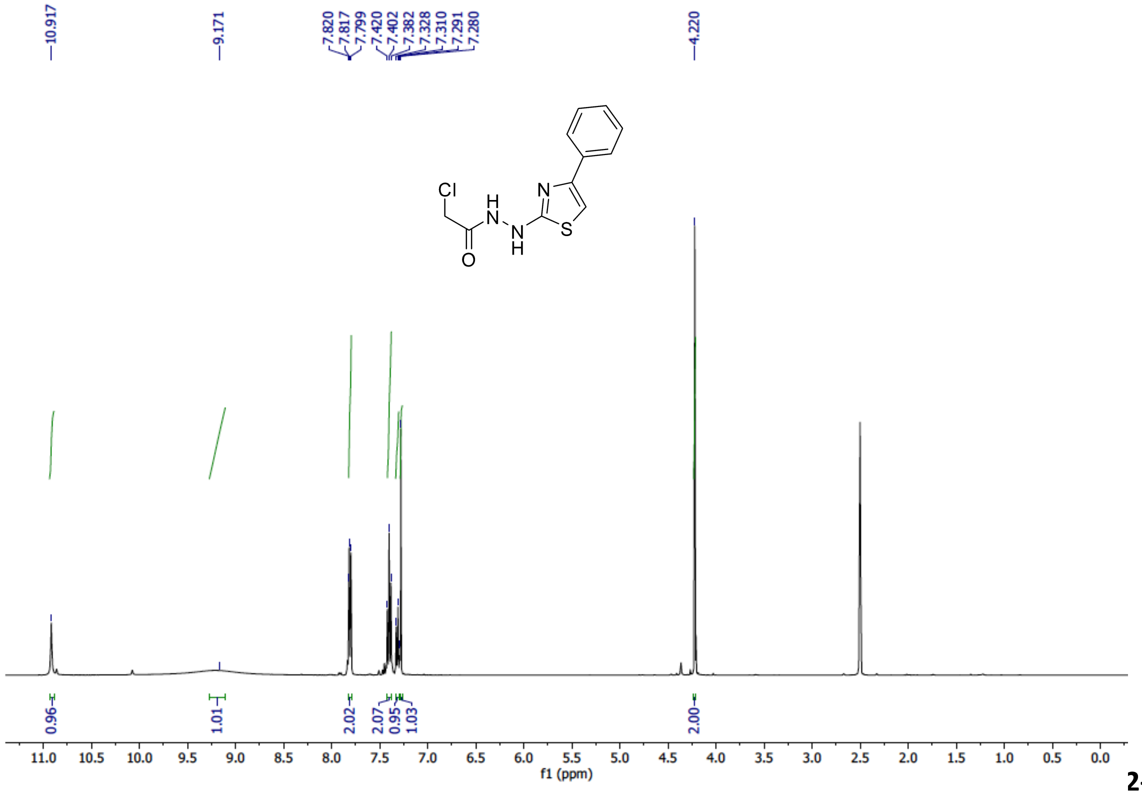

2-Chloro-*N*'-(4-phenylthiazol-2-yl)acetohydrazide (PreSSR-1)

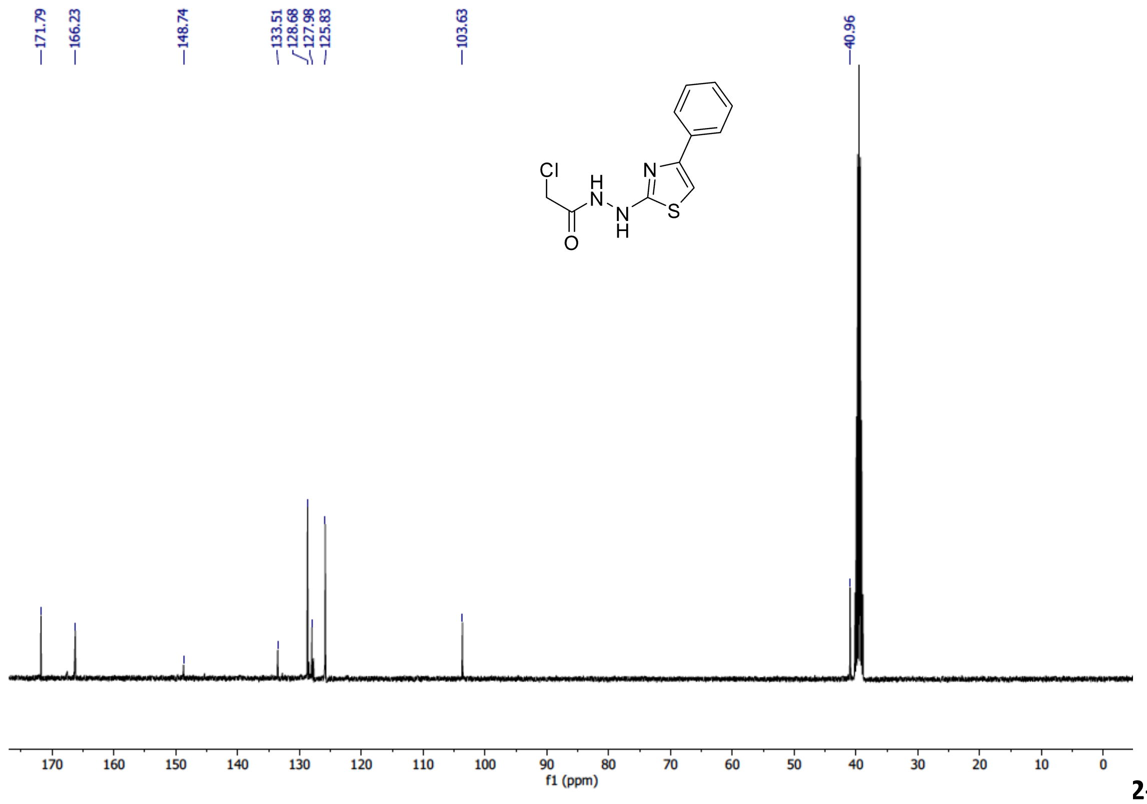

2-Chloro-*N*'-(4-phenylthiazol-2-yl)acetohydrazide (PreSSR-1)

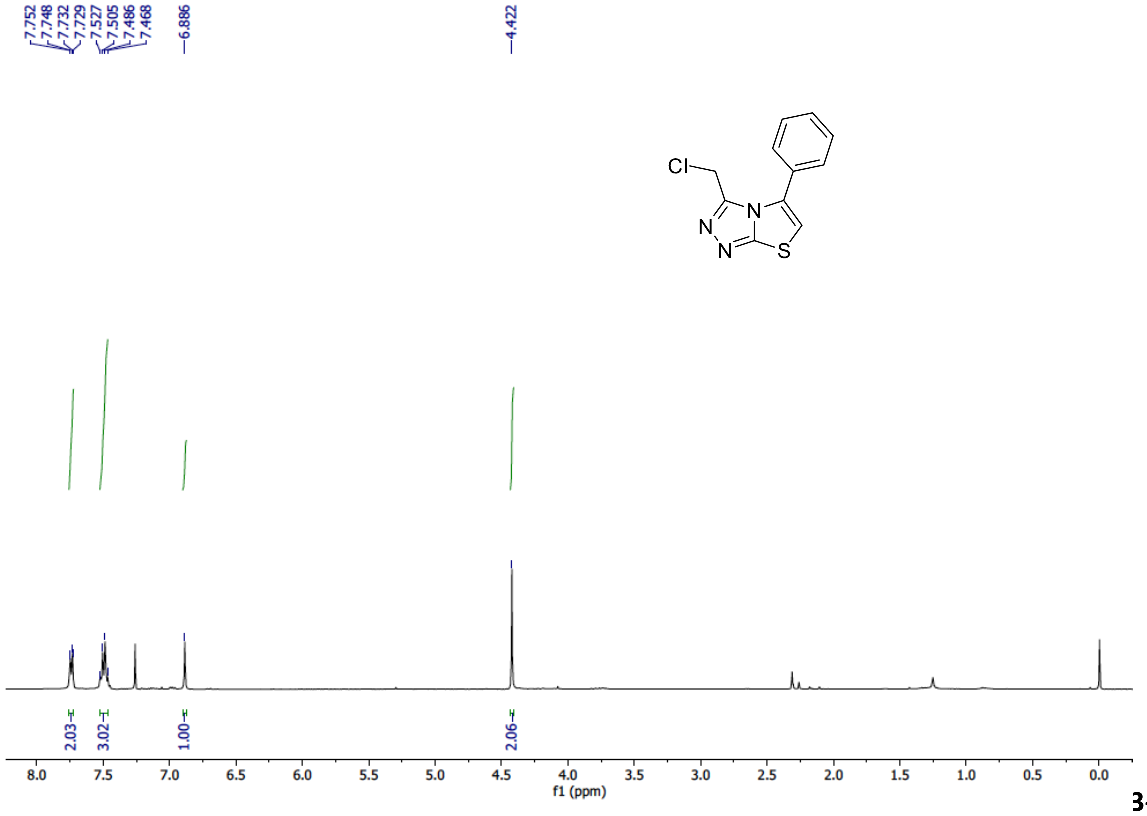

3-(Chloromethyl)-5-phenylthiazolo[2,3-*c*][1,2,4]triazole (SSR-1)

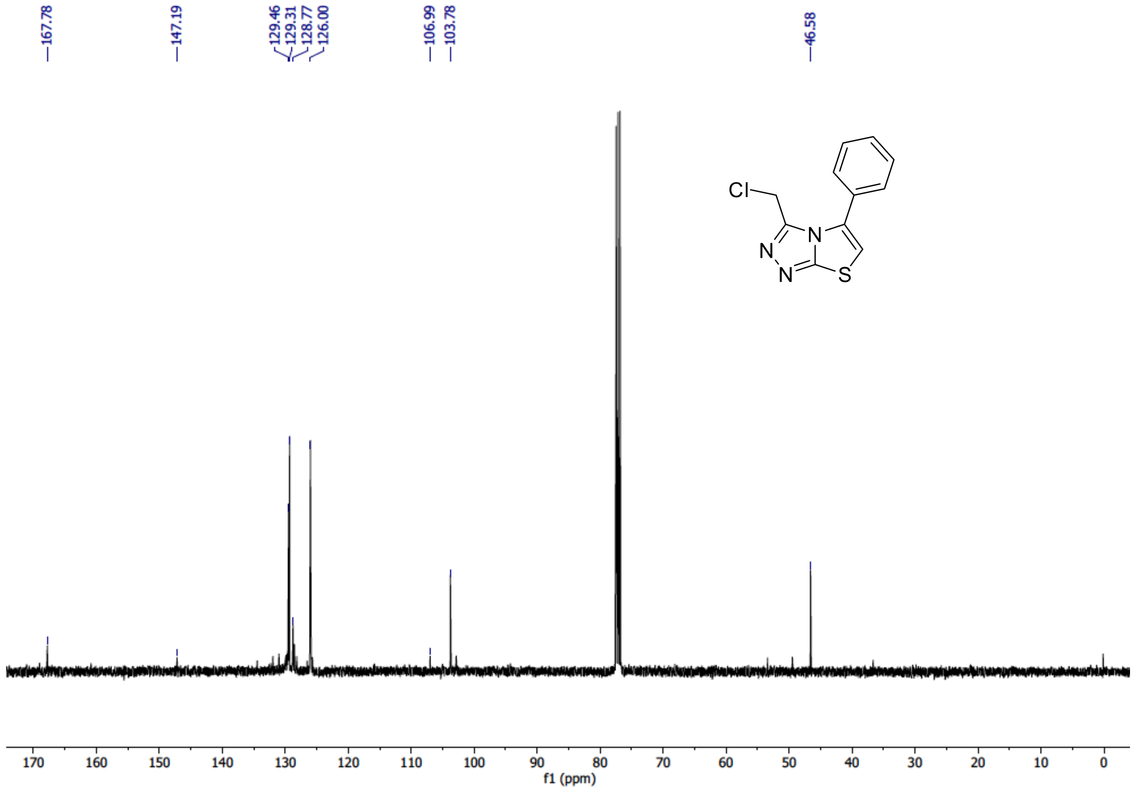

3-(Chloromethyl)-5-phenylthiazolo[2,3-*c*][1,2,4]triazole (SSR-1)

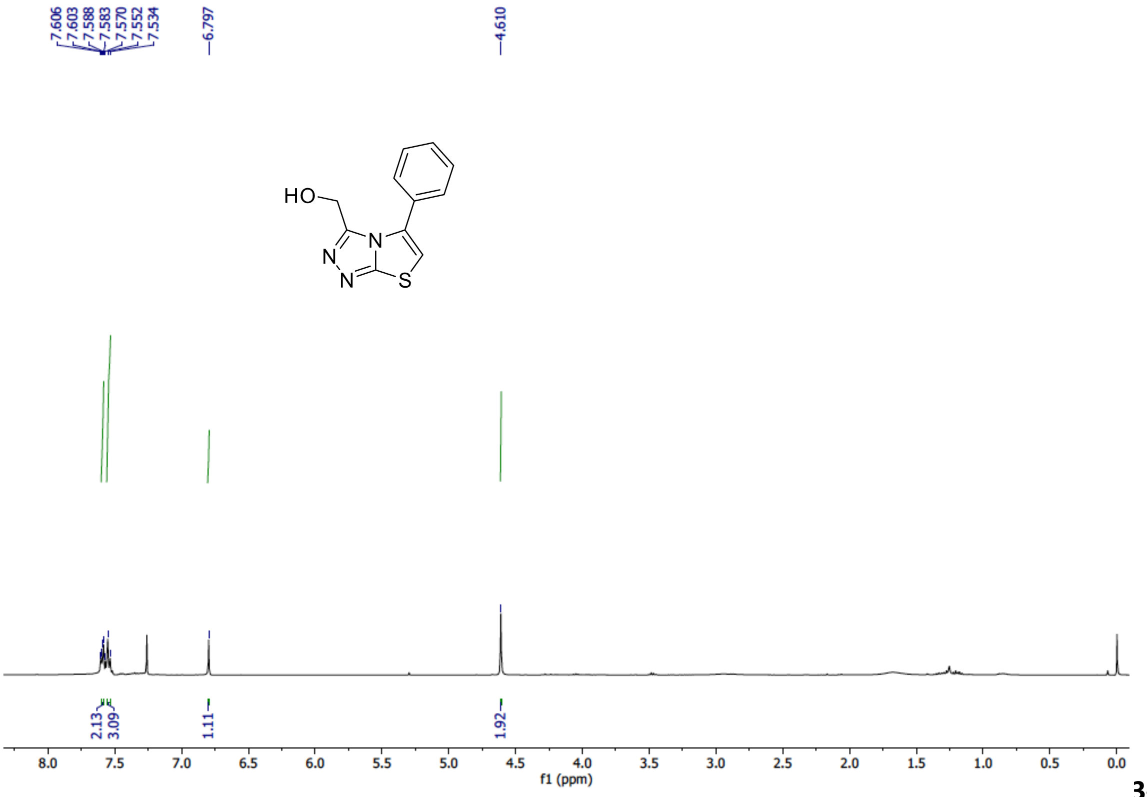

3-(Hydroxymethyl)-5-phenylthiazolo[2,3-*c*][1,2,4]triazole (SSR-2)

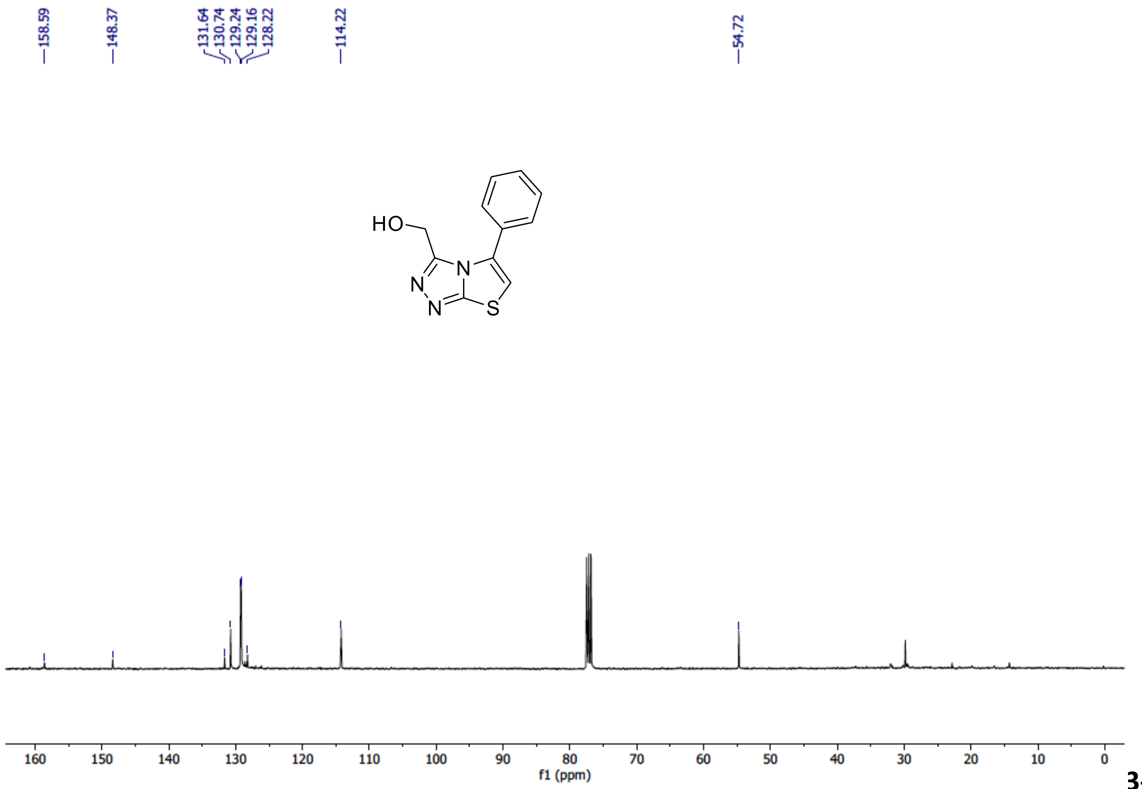

3-(Hydroxymethyl)-5-phenylthiazolo[2,3-*c*][1,2,4]triazole (SSR-2)

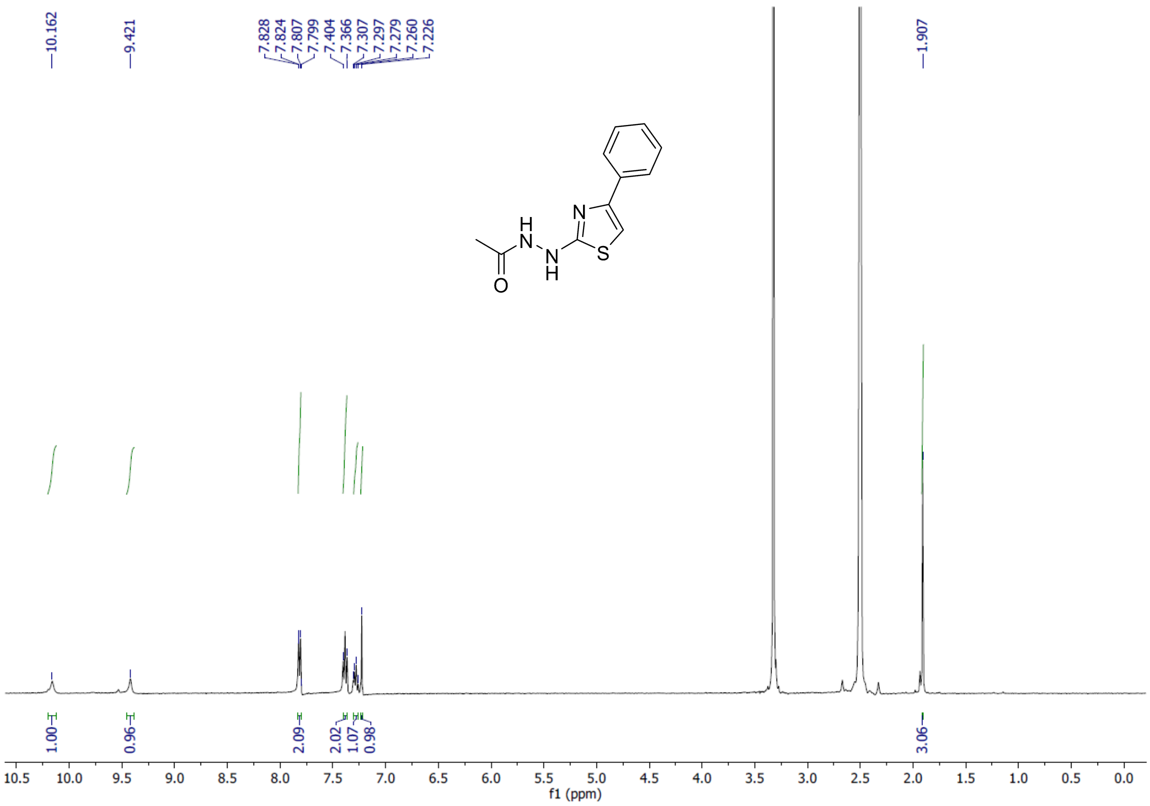

***N*'-(4-Phenylthiazol-2-yl)acetohydrazide (PreSSR-3)**

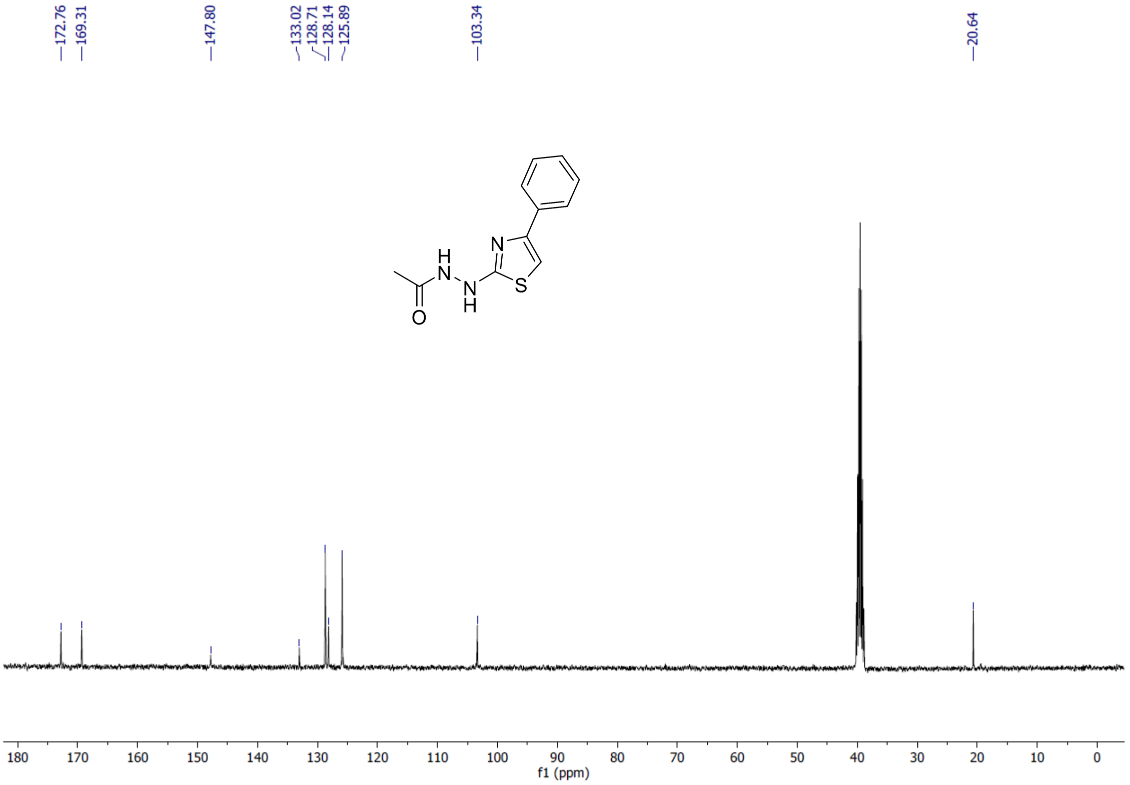

***N*'-(4-Phenylthiazol-2-yl)acetohydrazide (PreSSR-3)**

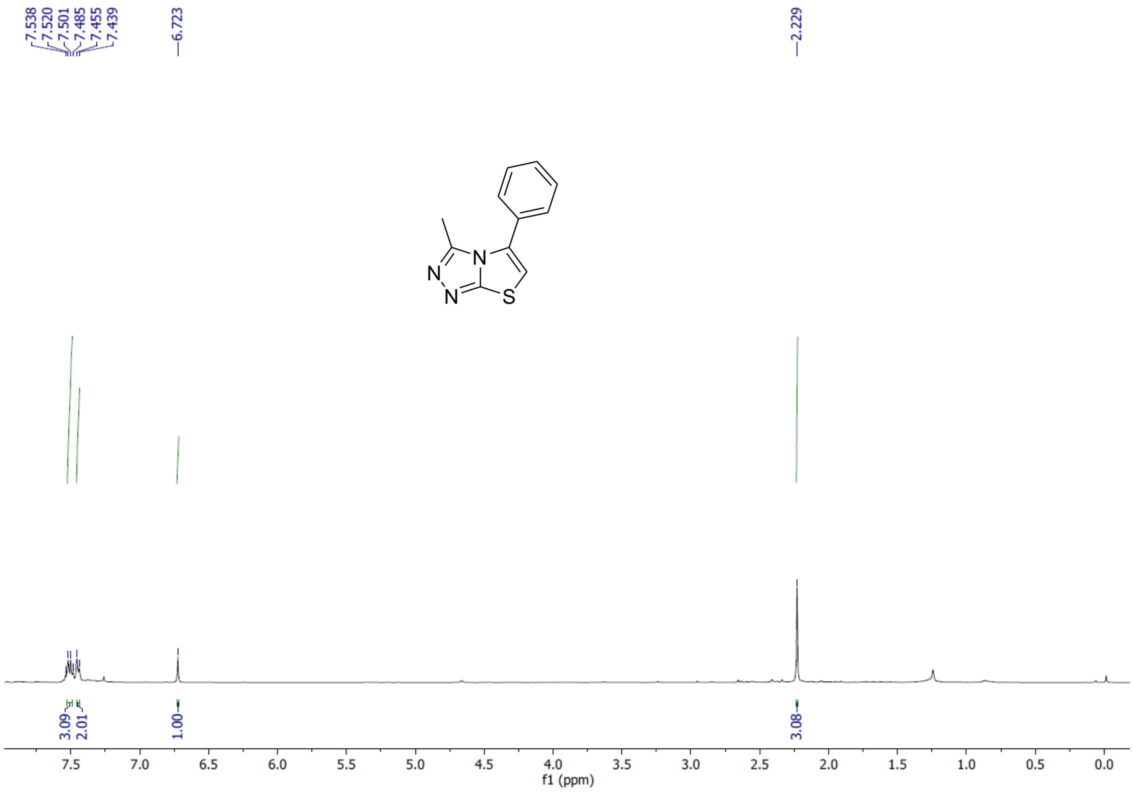

3-Methyl-5-phenylthiazolo[2,3-*c*][1,2,4]triazole (SSR-3)

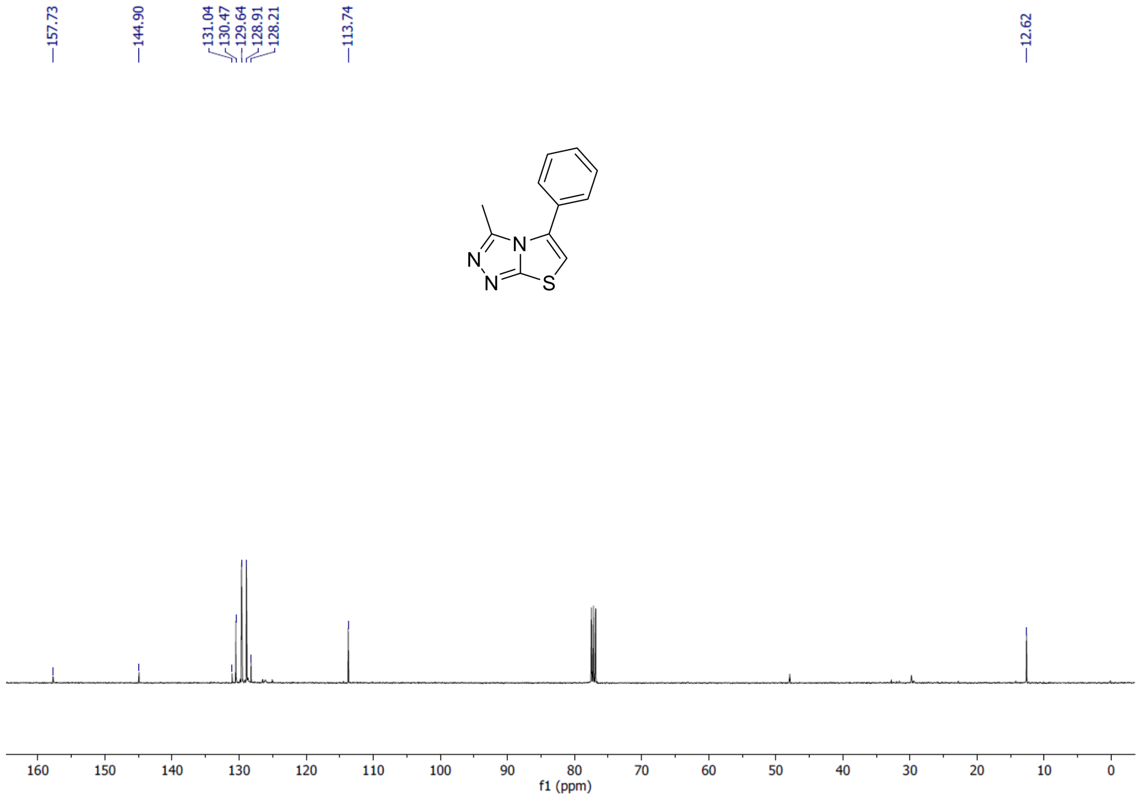

3-Methyl-5-phenylthiazolo[2,3-*c*][1,2,4]triazole (SSR-3)

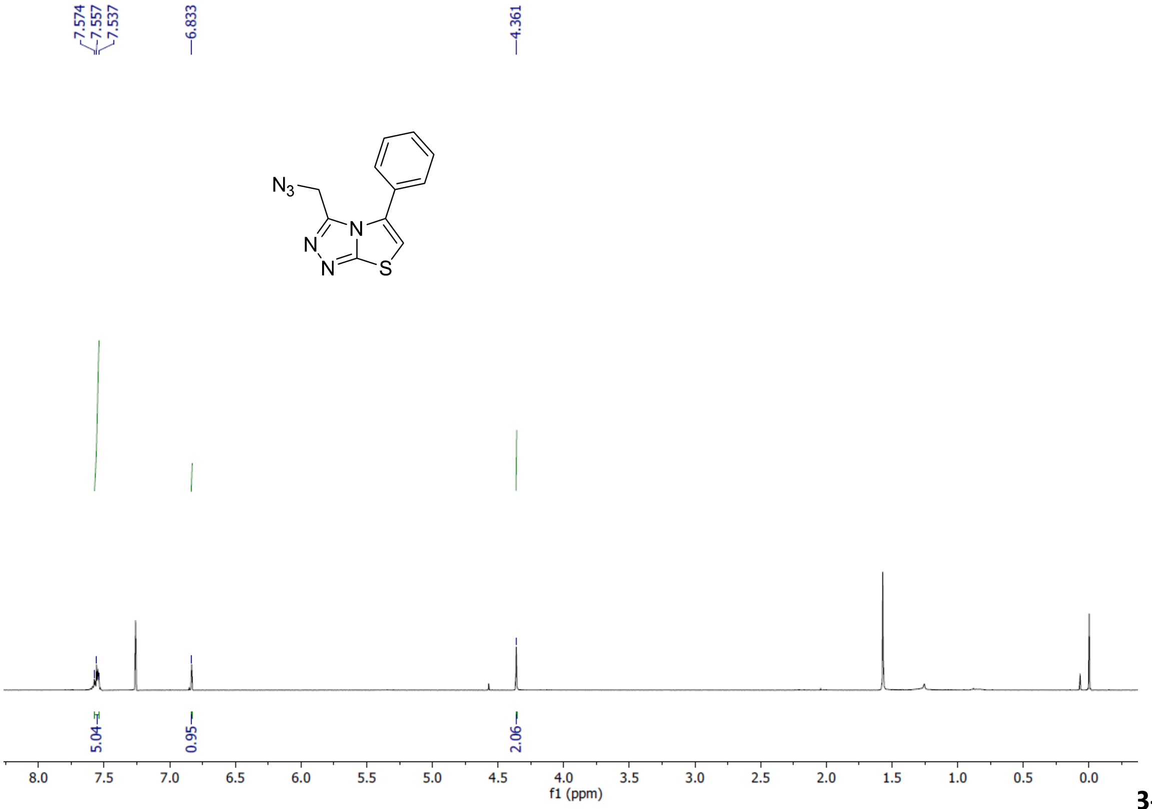

3-(Azidomethyl)-5-phenylthiazolo[2,3-*c*][1,2,4]triazole (SSR-N_3)

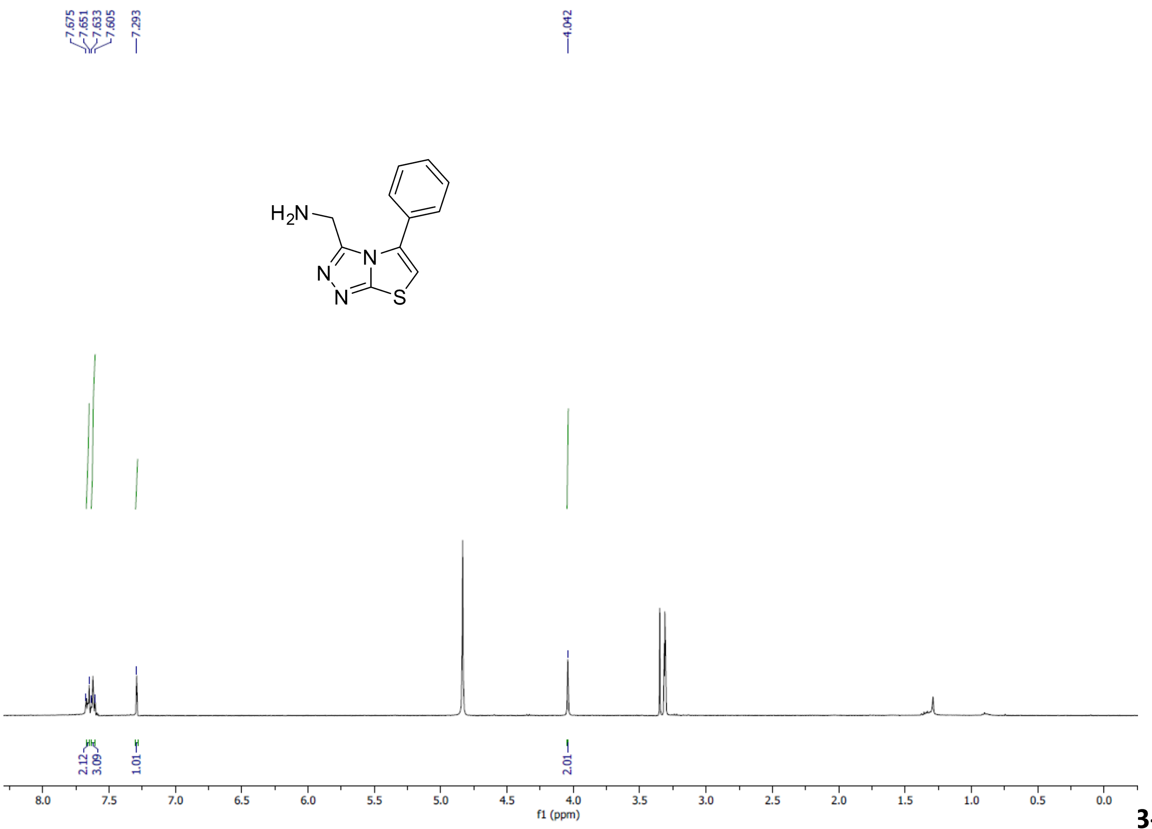

3-(Aminomethyl)-5-phenylthiazolo[2,3-*c*][1,2,4]triazole (SSR-6)

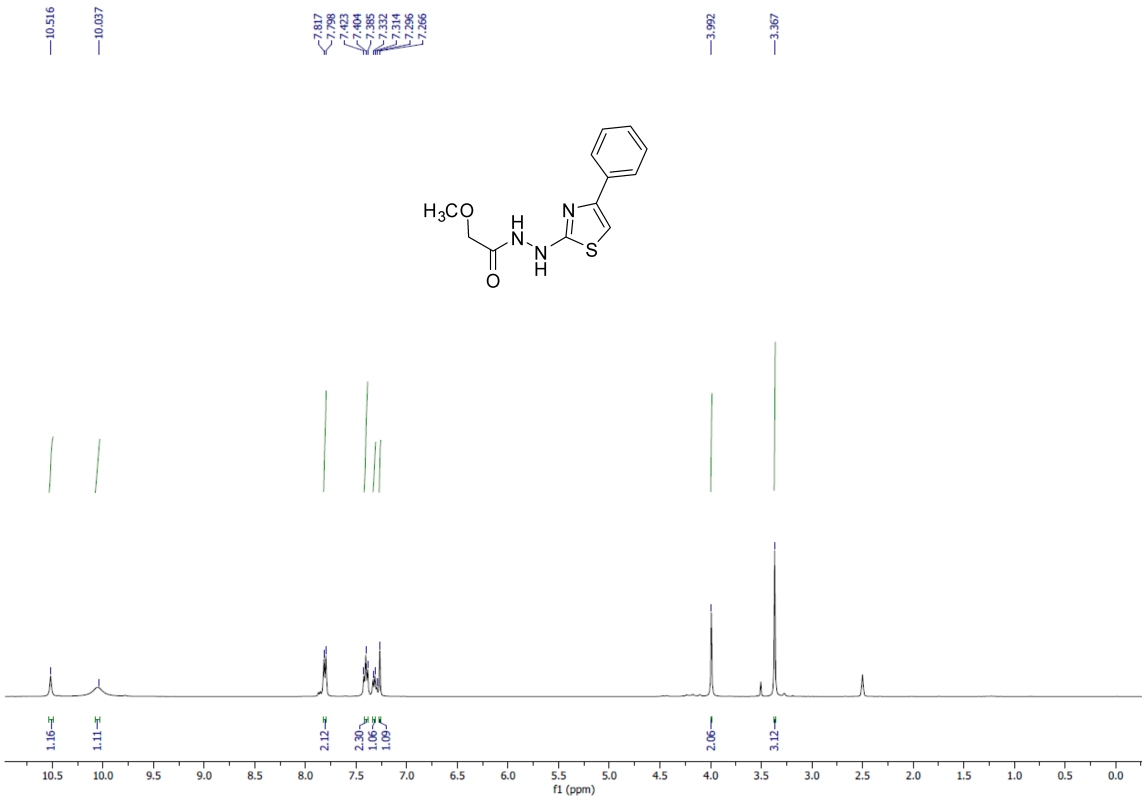

2-Methoxy-*N*'-(4-phenylthiazol-2-yl)acetohydrazide (PreSSR-7)

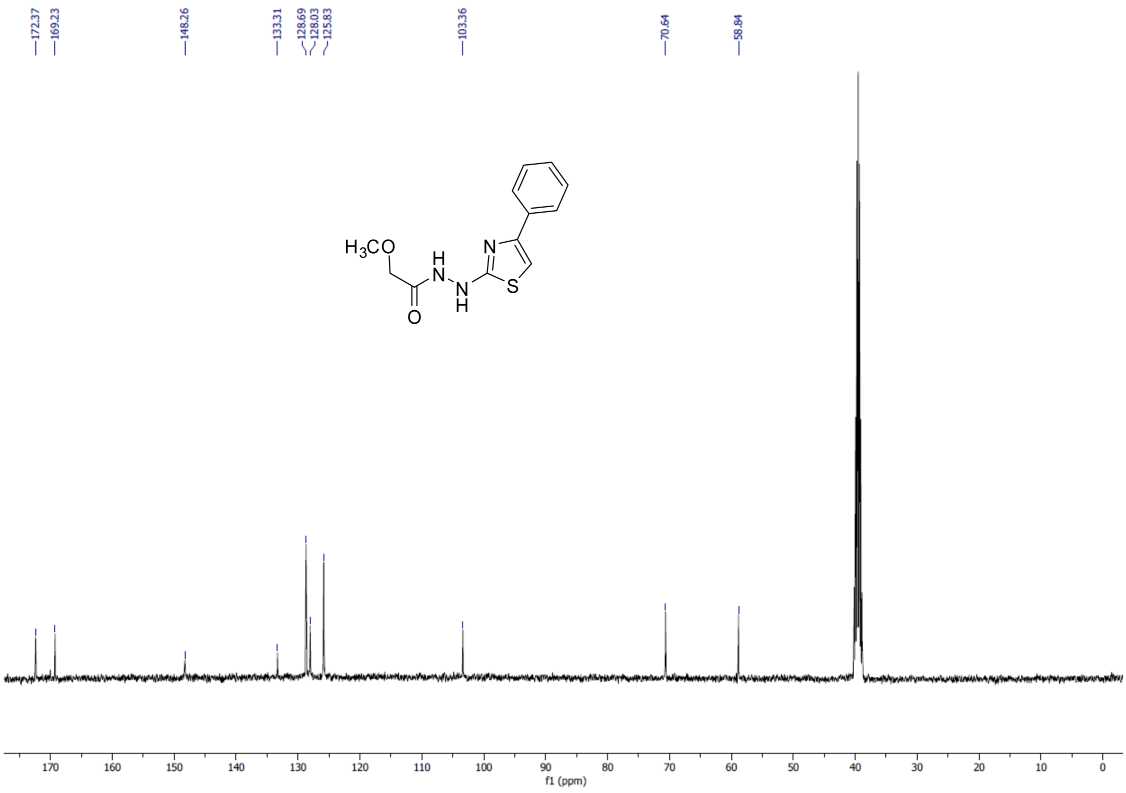

2-Methoxy-*N*'-(4-phenylthiazol-2-yl)acetohydrazide (PreSSR-7)

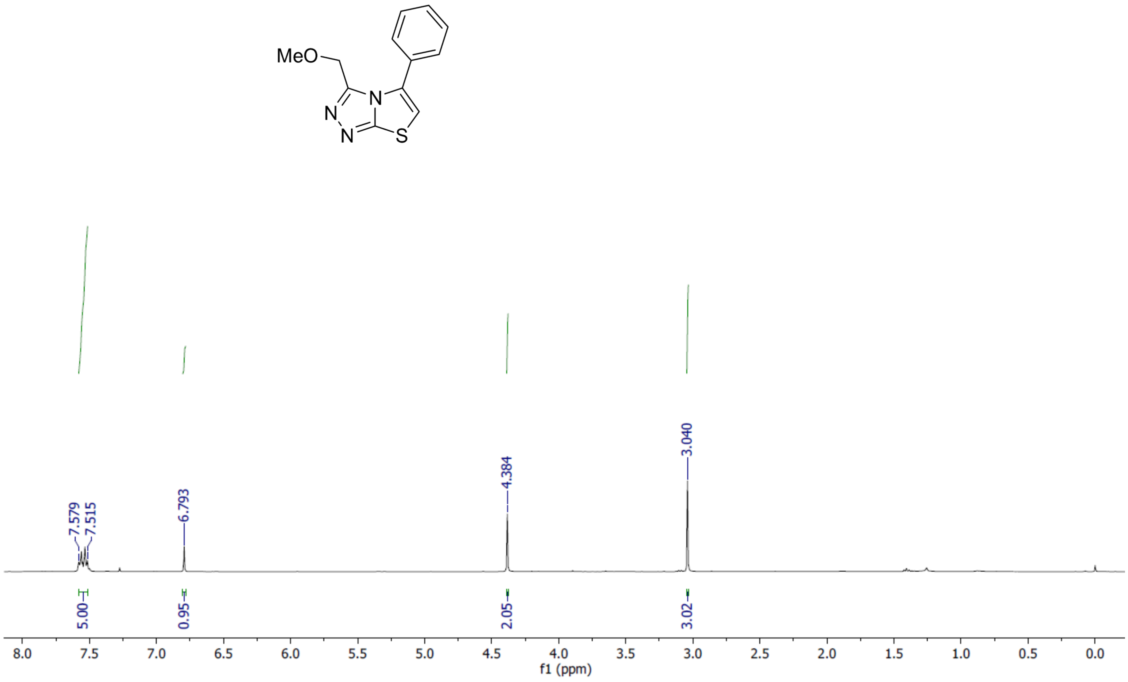

3-(Methoxymethyl)-5-phenylthiazolo[2,3-*c*][1,2,4]triazole (SSR-7)

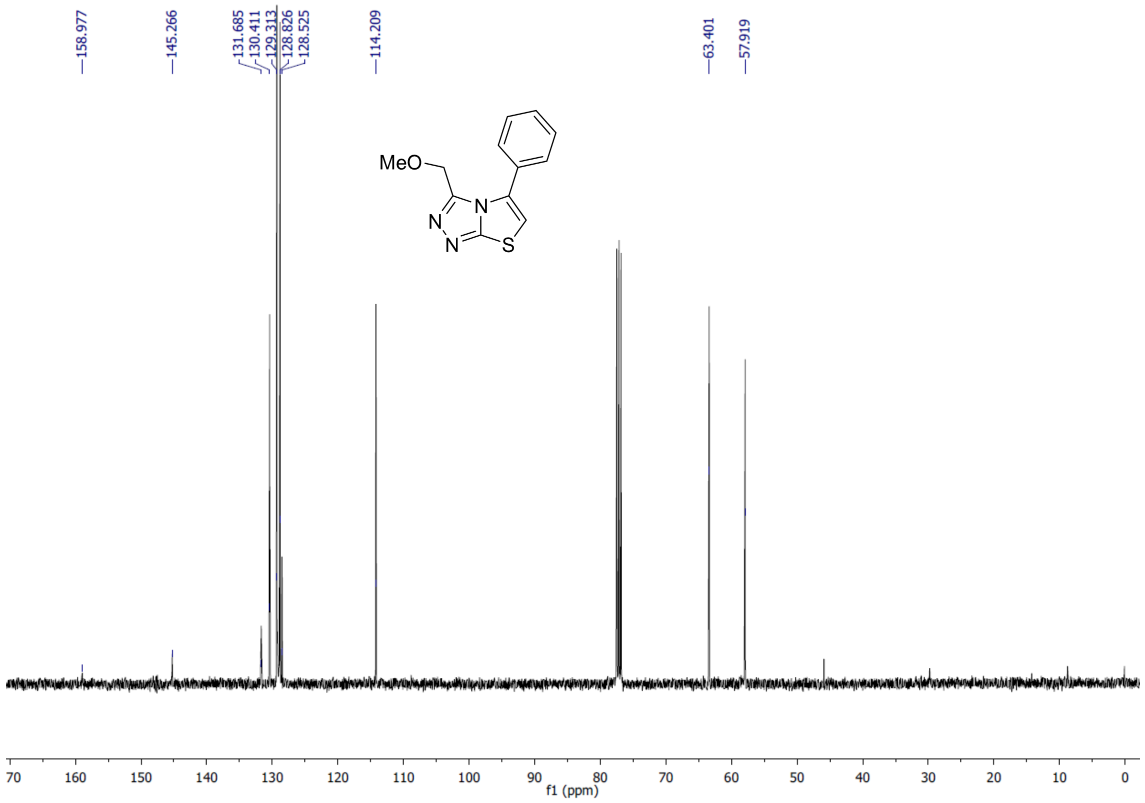

3-(Methoxymethyl)-5-phenylthiazolo[2,3-*c*][1,2,4]triazole (SSR-7)

HPLC/MS analysis of new compounds

3-(Hydroxymethyl)-5-phenylthiazolo[2,3-*c*][1,2,4]triazole (SSR-2)

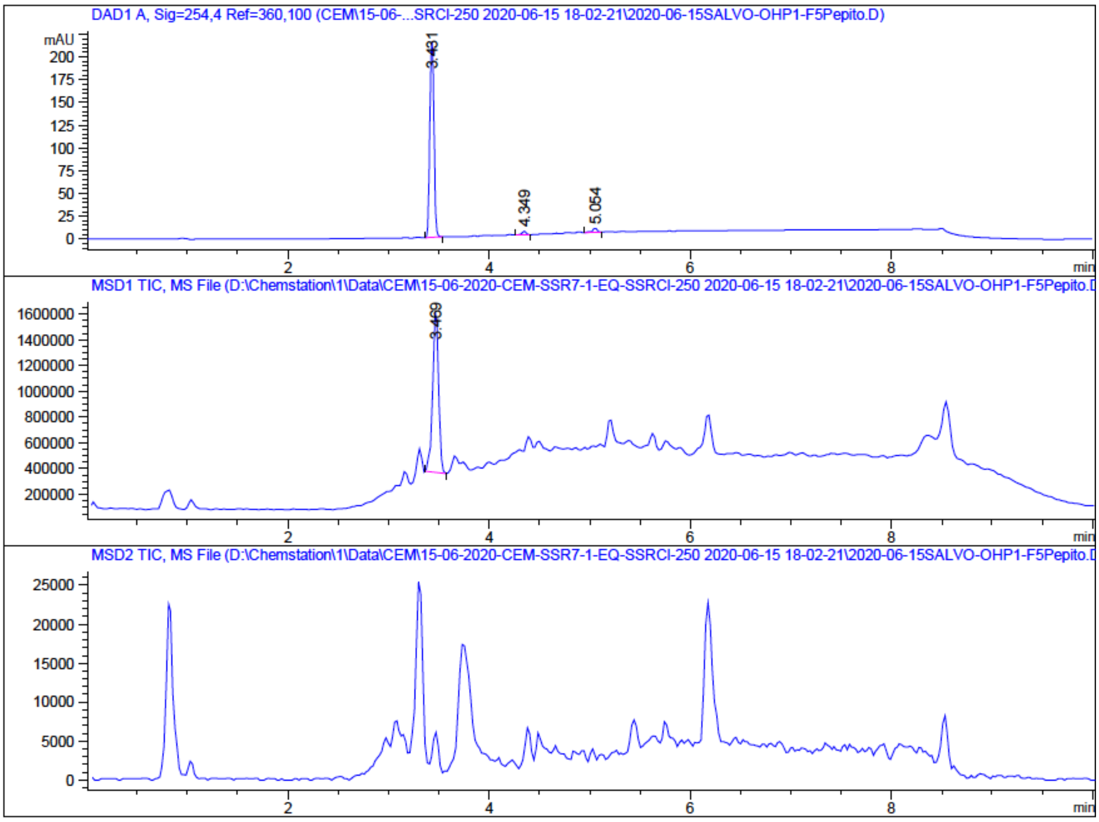

===

Area Percent Report

===

Sorted By : Signal
Multiplier : 1.0000
Dilution : 1.0000
Do not use Multiplier & Dilution Factor with ISTDs

Signal 1: DAD1 A, Sig=254,4 Ref=360,100

Peak #	RetTime [min]	Type	Width [min]	Area [mAU*s]	Height [mAU]	Area %
1	3.431	BB	0.0472	614.90924	215.97429	95.6650
2	4.349	BB	0.0485	12.29305	3.93260	1.9125
3	5.054	BB	0.0528	15.57113	4.45277	2.4225
Totals :				642.77342	224.35966	

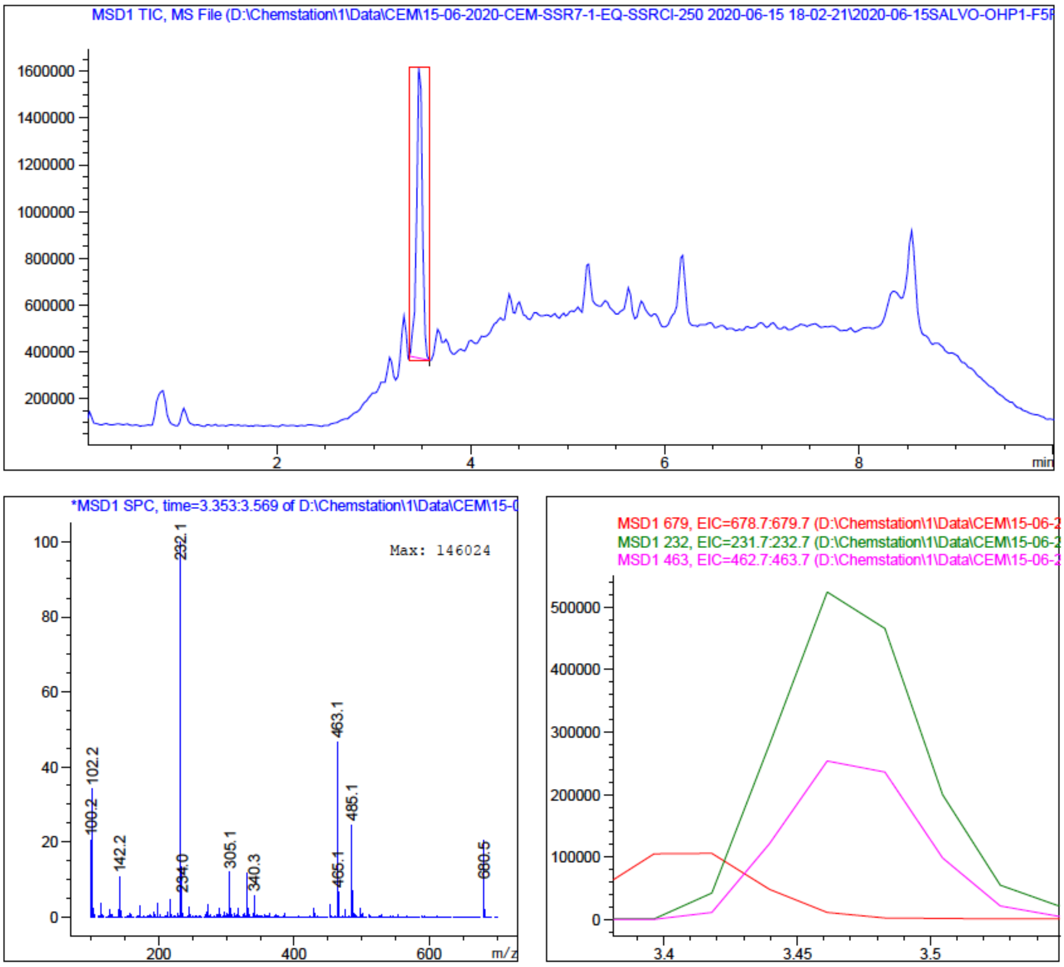
MSD1 TIC, MS File (D:\Chemstation\1\Data\CEM\15-06-2020-CEM-SSR7-1-EQ-SSRCI-250 2020-06-15 18-02-21\2020-06-15SALVO-OHP1-F5F
1600000
1400000
1200000
1000000
800000
600000
400000
200000
2
4
6
8
min
*MSD1 SPC, time=3.353:3.569 of D:\Chemstation\1\Data\CEM\15-0
Max: 146024
100
80
60
40
20
0
232.1
463.1
485.1
102.2
100.2
142.2
234.0
305.1
340.3
465.1
680.5
200
400
600
m/z
MSD1 679, EIC=678.7:679.7 (D:\Chemstation\1\Data\CEM\15-06-2
MSD1 232, EIC=231.7:232.7 (D:\Chemstation\1\Data\CEM\15-06-2
MSD1 463, EIC=462.7:463.7 (D:\Chemstation\1\Data\CEM\15-06-2
500000
400000
300000
200000
100000
0
3.4
3.45
3.5

3-Methyl-5-phenylthiazolo[2,3-*c*][1,2,4]triazole (SSR-3)

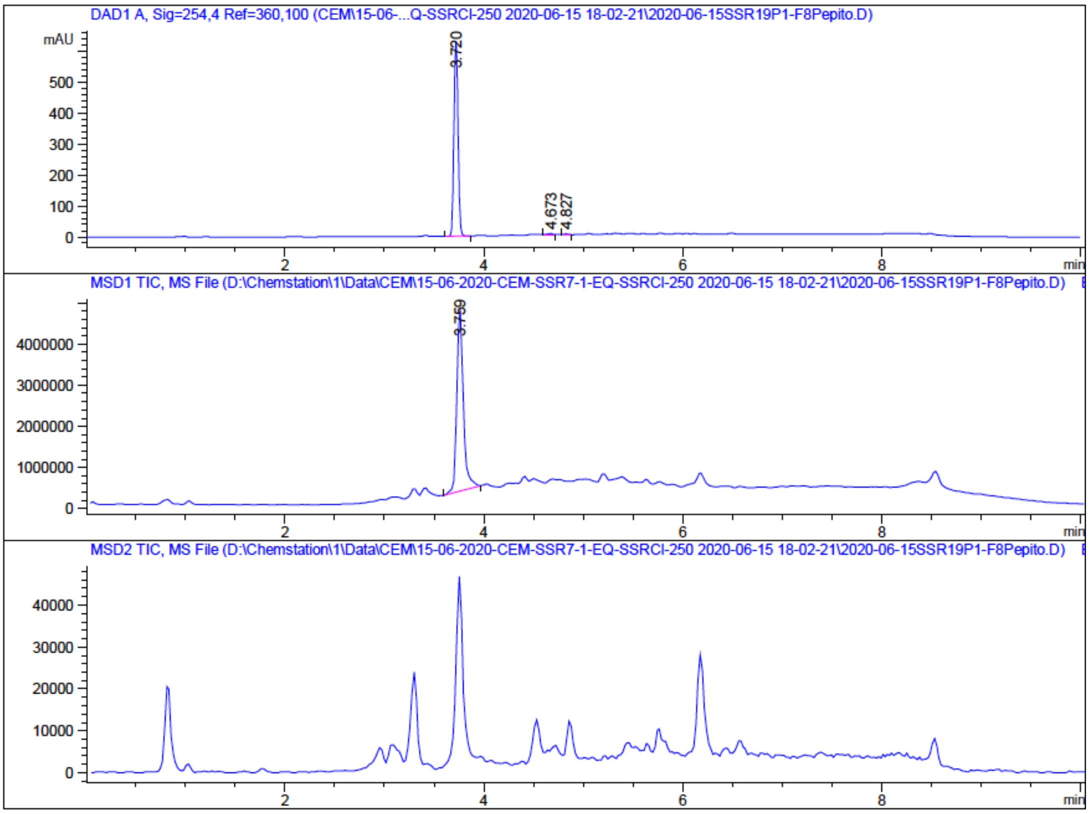

===

Area Percent Report

===

Sorted By : Signal
Multiplier : 1.0000
Dilution : 1.0000
Do not use Multiplier & Dilution Factor with ISTDs

Signal 1: DAD1 A, Sig=254,4 Ref=360,100

Peak #	RetTime [min]	Type	Width [min]	Area [mAU*s]	Height [mAU]	Area %
1	3.720	BB	0.0459	1836.90613	632.00922	98.9354
2	4.673	BB	0.0518	11.93774	3.33148	0.6430
3	4.827	BB	0.0443	7.82875	2.82401	0.4217

Totals : 1856.67261 638.16470

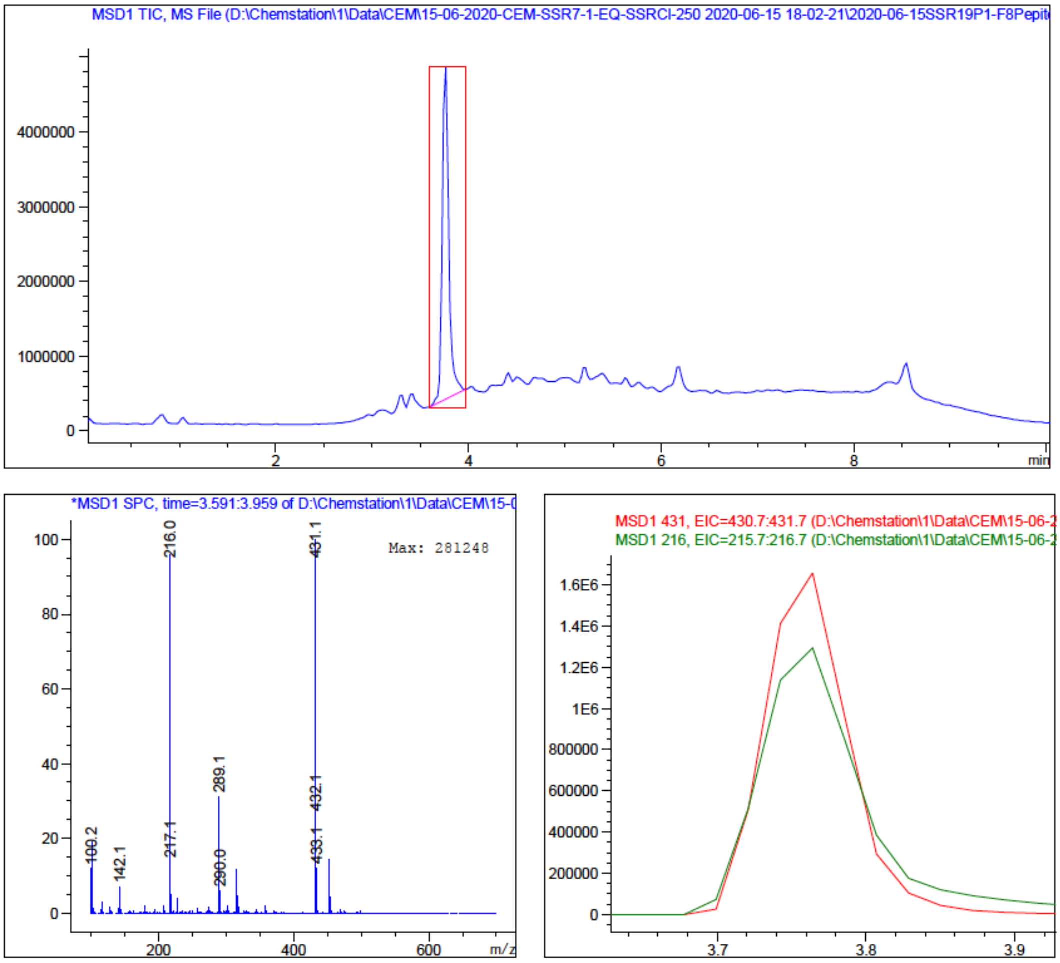
MSD1 TIC, MS File (D:\Chemstation\1\Data\CEM\15-06-2020-CEM-SSR7-1-EQ-SSRCI-250 2020-06-15 18-02-21\2020-06-15SSR19P1-F8Pepit
4000000
3000000
2000000
1000000
0
2
4
6
8
min
*MSD1 SPC, time=3.591:3.959 of D:\Chemstation\1\Data\CEM\15-0
Max: 281248
100
80
60
40
20
0
216.0
431.1
289.1
432.1
433.1
217.1
290.0
109.2
142.1
200
400
600
m/z
MSD1 431, EIC=430.7:431.7 (D:\Chemstation\1\Data\CEM\15-06-2
MSD1 216, EIC=215.7:216.7 (D:\Chemstation\1\Data\CEM\15-06-2
1.6E6
1.4E6
1.2E6
1E6
800000
600000
400000
200000
0
3.7
3.8
3.9

3-(Aminomethyl)-5-phenylthiazolo[2,3-*c*][1,2,4]triazole (SSR-6)

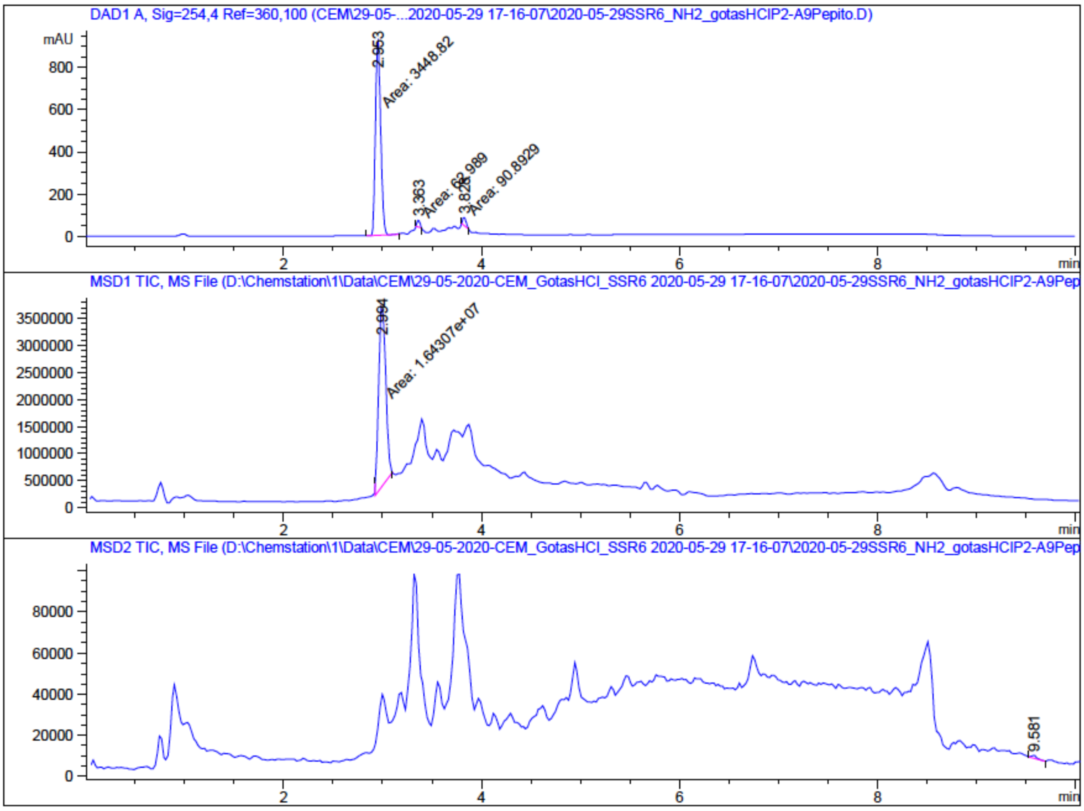


```
=====================================================================
                         Area Percent Report
=====================================================================

Sorted By             :      Signal
Multiplier            :      1.0000
Dilution              :      1.0000
Do not use Multiplier & Dilution Factor with ISTDs


Signal 1: DAD1 A, Sig=254,4 Ref=360,100

Peak RetTime Type  Width     Area      Height     Area
  #   [min]        [min]   [mAU*s]     [mAU]       %
----|-------|----|-------|----------|----------|--------|
   1   2.953 MM    0.0618 3448.82031  929.85583  95.7287
   2   3.363 MM    0.0340   62.98902   30.86420   1.7484
   3   3.828 MM    0.0399   90.89293   37.98321   2.5229

Totals :                  3602.70226  998.70325
```

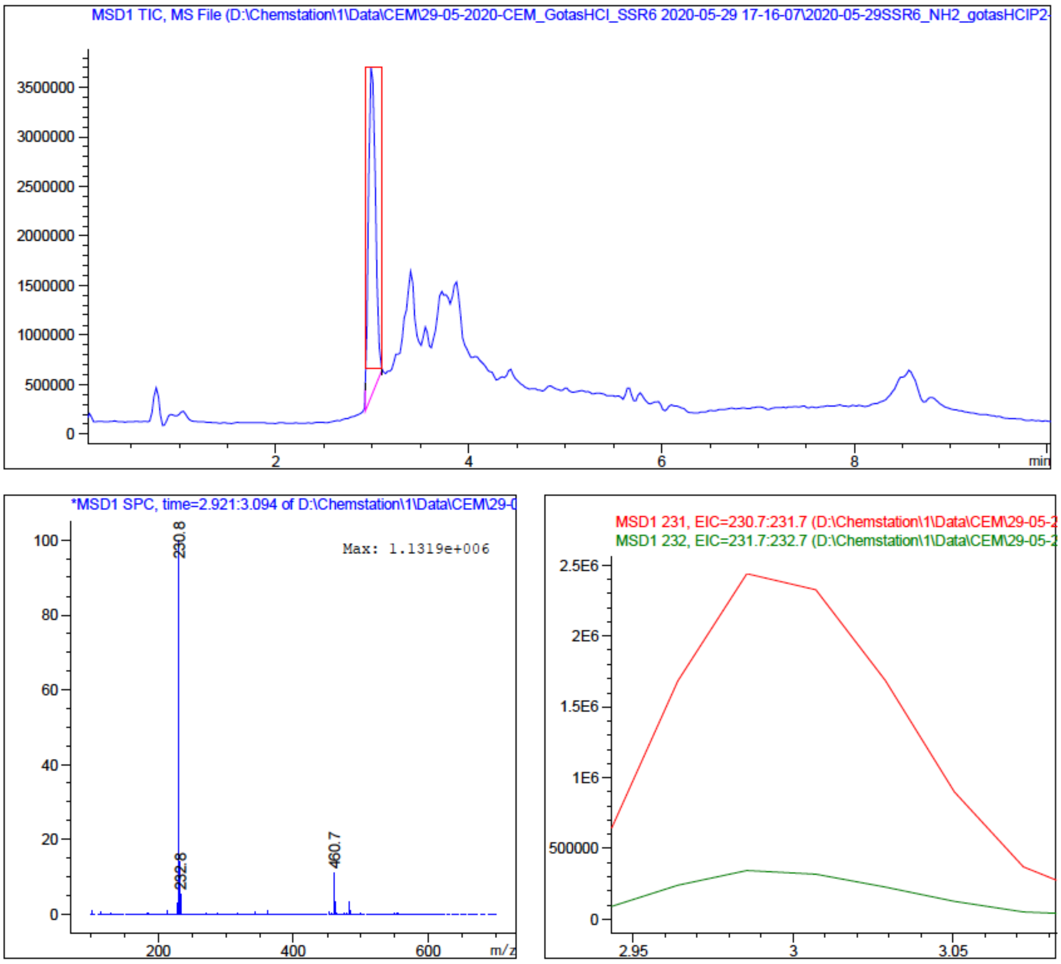
MSD1 TIC, MS File (D:\Chemstation\1\Data\CEM\29-05-2020-CEM_GotasHCl_SSR6 2020-05-29 17-16-07\2020-05-29SSR6_NH2_gotasHClP2
3500000
3000000
2500000
2000000
1500000
1000000
500000
0
2
4
6
8
min
*MSD1 SPC, time=2.921:3.094 of D:\Chemstation\1\Data\CEM\29-0
Max: 1.1319e+006
230.8
232.8
460.7
100
80
60
40
20
0
200
400
600
m/z
MSD1 231, EIC=230.7:231.7 (D:\Chemstation\1\Data\CEM\29-05-2
MSD1 232, EIC=231.7:232.7 (D:\Chemstation\1\Data\CEM\29-05-2
2.5E6
2E6
1.5E6
1E6
500000
0
2.95
3
3.05

3-(Methoxymethyl)-5-phenylthiazolo[2,3-*c*][1,2,4]triazole (SSR-7)

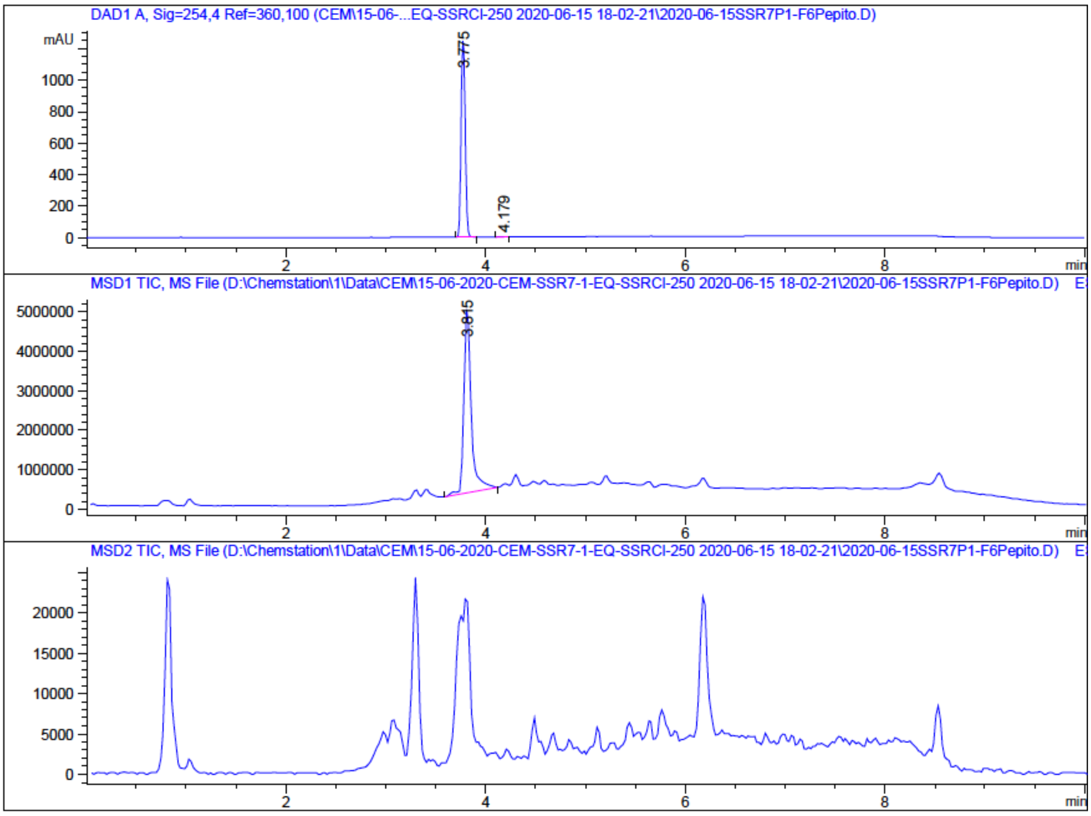

===

Area Percent Report

===

Sorted By : Signal

Multiplier : 1.0000

Dilution : 1.0000

Do not use Multiplier & Dilution Factor with ISTDs

Signal 1: DAD1 A, Sig=254,4 Ref=360,100

Peak #	RetTime [min]	Type	Width [min]	Area [mAU*s]	Height [mAU]	Area %
1	3.775	BB	0.0477	3602.73730	1245.94885	99.7270
2	4.179	BB	0.0552	9.86081	2.79220	0.2730

Totals : 3612.59811 1248.74106

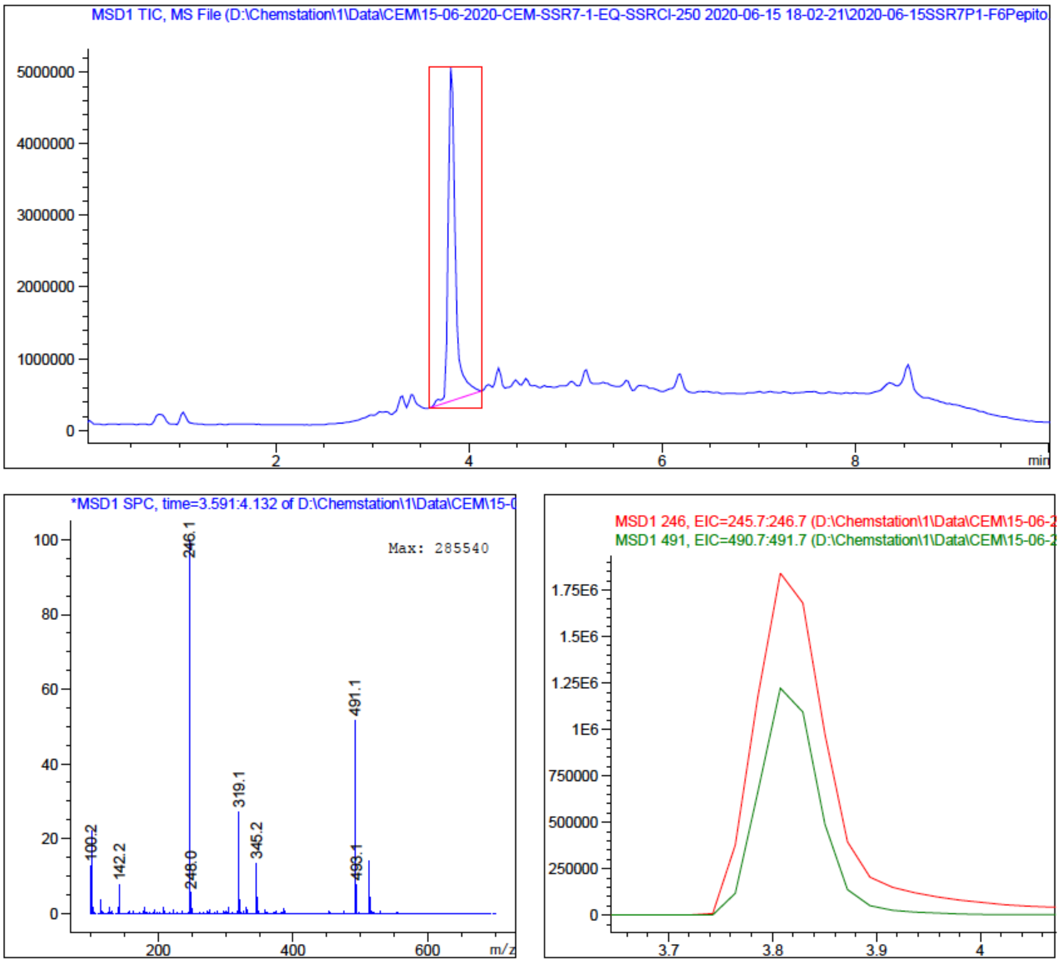
MSD1 TIC, MS File (D:\Chemstation\1\Data\CEM\15-06-2020-CEM-SSR7-1-EQ-SSRCI-250 2020-06-15 18-02-21\2020-06-15SSR7P1-F6Pepito
5000000
4000000
3000000
2000000
1000000
0
2
4
6
8
min
*MSD1 SPC, time=3.591:4.132 of D:\Chemstation\1\Data\CEM\15-0
Max: 285540
100
80
60
40
20
0
246.1
491.1
319.1
345.2
100.2
142.2
248.0
493.1
200
400
600
m/z
MSD1 246, EIC=245.7:246.7 (D:\Chemstation\1\Data\CEM\15-06-2
MSD1 491, EIC=490.7:491.7 (D:\Chemstation\1\Data\CEM\15-06-2
1.75E6
1.5E6
1.25E6
1E6
750000
500000
250000
0
3.7
3.8
3.9
4

Molecular Formula Strings (SMILES)

Main text ID	Synthesis ID	SMILES
**n.a.**	**SSR-1**	ClCC1=NN=C2N1C(C3=CC=CC=C3)=CS2
**4**	**SSR-2**	OCC1=NN=C2N1C(C3=CC=CC=C3)=CS2
**2**	**SSR-3**	CC1=NN=C2N1C(C3=CC=CC=C3)=CS2
**5**	**SSR-6**	NCC1=NN=C2N1C(C3=CC=CC=C3)=CS2
**3**	**SSR-7**	COCC1=NN=C2N1C(C3=CC=CC=C3)=CS2
**1**	**n.a.**	CCC1=NN=C2N1C(C3=CC=CC=C3)=CS2

REFERENCES

[1] O. Gilan, I. Rioja, K. Knezevic, M. J. Bell, M. M. Yeung, N. R. Harker, E. Y. N. Lam, C.-W. Chung, P. Bamborough, M. Petretich, M. Urh, S. J. Atkinson, A. K. Bassil, E. J. Roberts, D. Vassiliadis, M. L. Burr, A. G. S. Preston, C. Wellaway, T. Werner, J. R. Gray, A.-M. Michon, T. Gobbetti, V. Kumar, P. E. Soden, A. Haynes, J. Vappiani, D. F. Tough, S. Taylor, S.-J. Dawson, M. Bantscheff, M. Lindon, G. Drewes, E. H. Demont, D. L. Daniels, P. Grandi, R. K. Prinjha, M. A. Dawson, "Selective targeting of BD1 and BD2 of the BET proteins in cancer and immunoinflammation" *Science* **2020**, *368*, 387–394.

[2] P. Filippakopoulos, S. Picaud, M. Mangos, T. Keates, J. P. Lambert, D. Barsyte-Lovejoy, I. Felletar, R. Volkmer, S. Müller, T. Pawson, A. C. Gingras, C. H. Arrowsmith, S. Knapp, "Histone recognition and large-scale structural analysis of the human bromodomain family" *Cell* **2012**, *149*, 214–231.

[3] A. Wyce, G. Ganji, K. N. Smitheman, C.-W. Chung, S. Korenchuk, Y. Bai, O. Barbash, B. Le, P. D. Craggs, M. T. McCabe, K. M. Kennedy-Wilson, L. V. Sanchez, R. L. Gosmini, N. Parr, C. F. McHugh, D. Dhanak, R. K. Prinjha, K. R. Auger, P. J. Tummino, "BET inhibition silences expression of MYCN and BCL2 and induces cytotoxicity in neuroblastoma tumor models" *PLoS One* **2013**, *8*, e72967.

[4] S. W. J. Ember, J.-Y. Zhu, S. H. Olesen, M. P. Martin, A. Becker, N. Berndt, G. I. Georg, E. Schönbrunn, "Acetyl-lysine binding site of bromodomain-containing protein 4 (BRD4) interacts with diverse kinase inhibitors" *ACS Chem Biol* **2014**, *9*, 1160–1171.

[5] S. Picaud, C. Wells, I. Felletar, D. Brotherton, S. Martin, P. Savitsky, B. Diez-Dacal, M. Philpott, C. Bountra, H. Lingard, O. Fedorov, S. Müller, P. E. Brennan, S. Knapp, P. Filippakopoulos, "RVX-208, an inhibitor of BET transcriptional regulators with selectivity for the second bromodomain" *Proc Natl Acad Sci U S A* **2013**, *110*, 19754–19759.

[6] A. M. Taylor, R. G. Vaswani, V. S. Gehling, M. C. Hewitt, Y. Leblanc, J. E. Audia, S. Bellon, R. T. Cummings, A. Côté, J.-C. Harmange, H. Jayaram, S. Joshi, J. M. Lora, J. A. Mertz, A. Neiss, E. Pardo, C. G. Nasveschuk, F. Poy, P. Sandy, J. W. Setser, R. J. Sims, Y. Tang, B. K. Albrecht, "Discovery of Benzotriazolo[4,3-d][1,4]diazepines as Orally Active Inhibitors of BET Bromodomains" *ACS Med Chem Lett* **2016**, *7*, 145–150.

[7] S. W. Ember, Q. T. Lambert, N. Berndt, S. Gunawan, M. Ayaz, M. Tauro, J.-Y. Zhu, P. J. Cranfill, P. Greninger, C. C. Lynch, C. H. Benes, H. R. Lawrence, G. W. Reuther, N. J. Lawrence, E. Schönbrunn, "Potent Dual BET Bromodomain-Kinase Inhibitors as Value-Added Multitargeted Chemical Probes and Cancer Therapeutics" *Mol Cancer Ther* **2017**, *16*, 1054–1067.

[8] M. Hügle, P. Regenass, R. Warstat, M. Hau, K. Schmidtkunz, X. Lucas, D. Wohlwend, O. Einsle, M. Jung, B. Breit, S. Günther, “4-Acyl Pyrroles as Dual BET-BRD7/9 Bromodomain Inhibitors Address BETi Insensitive Human Cancer Cell Lines” *J Med Chem* **2020**, *63*, 15603–15620.

[9] S. G. Piticchio, M. Martínez-Cartró, S. Scaffidi, M. Rachman, S. Rodriguez-Arevalo, A. Sanchez-Arfelis, C. Escolano, S. Picaud, T. Krojer, P. Filippakopoulos, F. von Delft, C. Galdeano, X. Barril, “Discovery of Novel BRD4 Ligand Scaffolds by Automated Navigation of the Fragment Chemical Space.” *Journal of medicinal chemistry* **2021**, *64*, 17887–17900.

[10] R. Gewald, C. Grunwald, U. Egerland, “Discovery of triazines as potent, selective and orally active PDE4 inhibitors” *Bioorg Med Chem Lett* **2013**, *23*, 4308–4314.

[11] T. Prosdocimi, L. Mollica, L. Mollica, S. Donini, M. S. Semrau, A. P. Lucarelli, E. Aiolfi, A. Cavalli, P. Storici, S. Alfei, C. Brullo, O. Bruno, E. Parisini, “Molecular Bases of PDE4D Inhibition by Memory-Enhancing GEBR Library Compounds” *Biochemistry* **2018**, *57*, 2876–2888.

[12] J. Larsen, M. Lambert, H. Pettersson, T. Vifian, M. Larsen, A. Ollerstam, P. Hegardt, C. Eskilsson, S. Laursen, A. Soehoel, T. Skak-Nielsen, L. M. Hansen, N. Ø. Knudsen, S. Eirefelt, M. D. Sørensen, T. G. Stilou, S. F. Nielsen, “Discovery and Early Clinical Development of Isobutyl 1-[8-Methoxy-5-(1-oxo-3*H*-isobenzofuran-5-yl)-[1,2,4]triazolo[1,5-*a*]pyridin-2-yl]cyclopropanecarboxylate (LEO 39652), a Novel ‘Dual-Soft’ PDE4 Inhibitor for Topical Treatment of Atopic Dermatitis” *J Med Chem* **2020**, *63*, 14502–14521.

[13] J. Liu, X. Zhang, G. Chen, Q. Shao, Y. Zou, Z. Li, H. Su, M. Li, Y. Xu, “Drug repurposing and structure-based discovery of new PDE4 and PDE5 inhibitors” *Eur J Med Chem* **2023**, *262*, 115893.

[14] G. L. Card, L. Blasdel, B. P. England, C. Zhang, Y. Suzuki, S. Gillette, D. Fong, P. N. Ibrahim, D. R. Artis, G. Bollag, M. V. Milburn, S.-H. Kim, J. Schlessinger, K. Y. J. Zhang, “A family of phosphodiesterase inhibitors discovered by cocrystallography and scaffold-based drug design.” *Nature biotechnology* **2005**, *23*, 201–7.

[15] Z. Zhu, Z.-Y. Sun, Y. Ye, J. Voigt, C. Strickland, E. M. Smith, J. Cumming, L. Wang, J. Wong, Y.-S. Wang, D. F. Wyss, X. Chen, R. Kuvelkar, M. E. Kennedy, L. Favreau, E. Parker, B. A. McKittrick, A. Stamford, M. Czarniecki, W. Greenlee, J. C. Hunter, “Discovery of Cyclic Acylguanidines as Highly Potent and Selective β-Site Amyloid Cleaving Enzyme (BACE) Inhibitors: Part I—Inhibitor Design and Validation” *J. Med. Chem.* **2010**, *53*, 951–965.

[16] M. Mandal, Z. Zhu, J. N. Cumming, X. Liu, C. Strickland, R. D. Mazzola, J. P. Caldwell, P. Leach, M. Grzelak, L. Hyde, Q. Zhang, G. Terracina, L. Zhang, X. Chen, R. Kuvelkar, M. E. Kennedy, L. Favreau, K. Cox, P. Orth, A. Buevich, J. Voigt, H. Wang, I. Kazakevich, B. A. McKittrick, W. Greenlee, E. M. Parker, A. W. Stamford, “Design and Validation of Bicyclic Iminopyrimidinones As Beta Amyloid Cleaving Enzyme-1 (BACE1) Inhibitors: Conformational Constraint to Favor a Bioactive Conformation” *J. Med. Chem.* **2012**, *55*, 9331–9345.

[17] K. Fujimoto, S. Yoshida, G. Tadano, N. Asada, K. Fuchino, S. Suzuki, E. Matsuoka, T. Yamamoto, S. Yamamoto, S. Ando, N. Kanegawa, Y. Tonomura, H. Ito, D. Moechars, F. J. R. Rombouts, H. J. M. Gijsen, K. Kusakabe, “Structure-Based Approaches to Improving Selectivity through Utilizing Explicit Water Molecules: Discovery of Selective β-Secretase (BACE1) Inhibitors over BACE2” *J. Med. Chem.* **2021**, *64*, 3075–3085.

[18] A. K. Ghosh, N. Kumaragurubaran, L. Hong, H. Lei, K. A. Hussain, C.-F. Liu, T. Devasamudram, V. Weerasena, R. Turner, G. Koelsch, G. Bilcer, J. Tang, “Design,

synthesis and X-ray structure of protein-ligand complexes: important insight into selectivity of memapsin 2 (beta-secretase) inhibitors" *J Am Chem Soc* **2006**, *128*, 5310–5311.

[19] H. Shimizu, A. Tosaki, K. Kaneko, T. Hisano, T. Sakurai, N. Nukina, "Crystal structure of an active form of BACE1, an enzyme responsible for amyloid beta protein production" *Mol Cell Biol* **2008**, *28*, 3663–3671.

[20] H. Rueeger, R. Lueoend, O. Rogel, J.-M. Rondeau, H. Möbitz, R. Machauer, L. Jacobson, M. Staufenbiel, S. Desrayaud, U. Neumann, "Discovery of cyclic sulfone hydroxyethylamines as potent and selective β-site APP-cleaving enzyme 1 (BACE1) inhibitors: structure-based design and in vivo reduction of amyloid β-peptides" *J Med Chem* **2012**, *55*, 3364–3386.

[21] T. A. Dineen, K. Chen, A. C. Cheng, K. Derakhchan, O. Epstein, J. Esmay, D. Hickman, C. E. Kreiman, I. E. Marx, R. C. Wahl, P. H. Wen, M. M. Weiss, D. A. Whittington, S. Wood, R. T. Fremeau, R. D. White, V. F. Patel, "Inhibitors of β-site amyloid precursor protein cleaving enzyme (BACE1): identification of (S)-7-(2-fluoropyridin-3-yl)-3-((3-methyloxetan-3-yl)ethynyl)-5'H-spiro[chromeno[2,3-b]pyridine-5,4'-oxazol]-2'-amine (AMG-8718)" *J Med Chem* **2014**, *57*, 9811–9831.

[22] A. B. Bueno, J. Agejas, H. Broughton, R. Dally, T. B. Durham, J. F. Espinosa, R. González, P. J. Hahn, A. Marcos, R. Rodríguez, G. Sanz, J. F. Soriano, D. Timm, P. Vidal, H.-C. Yang, J. R. McCarthy, "Optimization of Hydroxyethylamine Transition State Isosteres as Aspartic Protease Inhibitors by Exploiting Conformational Preferences" *J Med Chem* **2017**, *60*, 9807–9820.

[23] M. Mandal, Z. Zhu, J. N. Cumming, X. Liu, C. Strickland, R. D. Mazzola, J. P. Caldwell, P. Leach, M. Grzelak, L. Hyde, Q. Zhang, G. Terracina, L. Zhang, X. Chen, R. Kuvelkar, M. E. Kennedy, L. Favreau, K. Cox, P. Orth, A. Buevich, J. Voigt, H. Wang, I. Kazakevich, B. A. McKittrick, W. Greenlee, E. M. Parker, A. W. Stamford, "Design and validation of bicyclic iminopyrimidinones as beta amyloid cleaving enzyme-1 (BACE1) inhibitors: conformational constraint to favor a bioactive conformation" *J Med Chem* **2012**, *55*, 9331–9345.

[24] Z. Zhu, Z.-Y. Sun, Y. Ye, J. Voigt, C. Strickland, E. M. Smith, J. Cumming, L. Wang, J. Wong, Y.-S. Wang, D. F. Wyss, X. Chen, R. Kuvelkar, M. E. Kennedy, L. Favreau, E. Parker, B. A. McKittrick, A. Stamford, M. Czarniecki, W. Greenlee, J. C. Hunter, "Discovery of cyclic acylguanidines as highly potent and selective beta-site amyloid cleaving enzyme (BACE) inhibitors: Part I--inhibitor design and validation" *J Med Chem* **2010**, *53*, 951–965.

[25] P. Filippakopoulos, J. Qi, S. Picaud, Y. Shen, W. B. Smith, O. Fedorov, E. M. Morse, T. Keates, T. T. Hickman, I. Felletar, M. Philpott, S. Munro, M. R. McKeown, Y. Wang, A. L. Christie, N. West, M. J. Cameron, B. Schwartz, T. D. Heightman, N. La Thangue, C. A. French, O. Wiest, A. L. Kung, S. Knapp, J. E. Bradner, "Selective inhibition of BET bromodomains" *Nature* **2010**, *468*, 1067–1073.

[26] C. W. Chung, H. Coste, J. H. White, O. Mirguet, J. Wilde, R. L. Gosmini, C. Delves, S. M. Magny, R. Woodward, S. A. Hughes, E. V. Boursier, H. Flynn, A. M. Bouillot, P. Bamborough, J. M. G. Brusq, F. O. J. Gellibert, E. J. Jones, A. M. Riou, P. Homes, S. L. Martin, I. J. Uings, J. Toum, C. A. Clément, A. B. Boullay, R. L. Grimley, F. M. Blandel, R. K. Prinjha, K. Lee, J. Kirilovsky, E. Nicodeme, "Discovery and characterization of small molecule inhibitors of the BET family bromodomains" *Journal of Medicinal Chemistry* **2011**, *54*, 3827–3838.

[27] W. Kabsch, "XDS." *Acta crystallographica. Section D, Biological crystallography* **2010**, *66*, 125–32.

[28] P. R. Evans, G. N. Murshudov, “How good are my data and what is the resolution?” *Acta Crystallographica Section D: Biological Crystallography* **2013**, *69*, 1204–1214.
[29] G. Winter, “Xia2: An expert system for macromolecular crystallography data reduction” *Journal of Applied Crystallography* **2010**, *43*, 186–190.
[30] C. Vonrhein, C. Flensburg, P. Keller, A. Sharff, O. Smart, W. Paciorek, T. Womack, G. Bricogne, “Data processing and analysis with the autoPROC toolbox” *Acta Crystallographica Section D: Biological Crystallography* **2011**, *67*, 293–302.
[31] M. D. Winn, C. C. Ballard, K. D. Cowtan, E. J. Dodson, P. Emsley, P. R. Evans, R. M. Keegan, E. B. Krissinel, A. G. W. Leslie, A. McCoy, S. J. McNicholas, G. N. Murshudov, N. S. Pannu, E. A. Potterton, H. R. Powell, R. J. Read, A. Vagin, K. S. Wilson, “Overview of the CCP4 suite and current developments” *Acta Crystallographica Section D: Biological Crystallography* **2011**, *67*, 235–242.
[32] F. Long, R. A. Nicholls, P. Emsley, S. Graæulis, A. Merkys, A. Vaitkus, G. N. Murshudov, “AceDRG: a stereochemical description generator for ligands.” *Acta crystallographica. Section D, Structural biology* **2017**, *73*, 112–122.
[33] O. S. Smart, T. O. Womack, A. Sharff, C. Flensburg, P. Keller, W. Paciorek, C. Vonrhein, G. Bricogne **2011**.
[34] G. N. Murshudov, P. Skubák, A. A. Lebedev, N. S. Pannu, R. A. Steiner, R. A. Nicholls, M. D. Winn, F. Long, A. A. Vagin, “REFMAC5 for the refinement of macromolecular crystal structures” *Acta Crystallographica Section D: Biological Crystallography* **2011**, *67*, 355–367.
[35] P. Emsley, B. Lohkamp, W. G. Scott, K. Cowtan, “Features and development of Coot” *Acta Crystallographica Section D: Biological Crystallography* **2010**, *66*, 486–501.
[36] V. B. Chen, W. B. Arendall, J. J. Headd, D. A. Keedy, R. M. Immormino, G. J. Kapral, L. W. Murray, J. S. Richardson, D. C. Richardson, “MolProbity: All-atom structure validation for macromolecular crystallography” *Acta Crystallographica Section D: Biological Crystallography* **2010**, *66*, 12–21.
[37] **2018**.
[38] M. J. Frisch, G. W. Trucks, H. B. Schlegel, G. E. Scuseria, M. A. Robb, J. R. Cheeseman, G. Scalmani, V. Barone, G. A. Petersson, H. Nakatsuji, X. Li, M. Caricato, A. Marenich, J. Bloino, B. G. Janesko, R. Gomperts, B. Mennucci, H. P. Hratchian, J. V. Ortiz, A. F. Izmaylov, J. L. Sonnenberg, D. Williams-Young, F. Ding, F. Lipparini, F. Egidi, J. Goings, B. Peng, A. Petrone, T. Henderson, D. Ranasinghe, V. G. Zakrzewski, J. Gao, N. Rega, G. Zheng, W. Liang, M. Hada, M. Ehara, K. Toyota, R. Fukuda, J. Hasegawa, M. Ishida, T. Nakajima, Y. Honda, O. Kitao, H. Nakai, T. Vreven, K. Throssell, J. A. Montgomery, Jr., J. E. Peralta, F. Ogliaro, M. Bearpark, J. J. Heyd, E. Brothers, K. N. Kudin, V. N. Staroverov, T. Keith, R. Kobayashi, J. Normand, K. Raghavachari, A. Rendell, J. C. Burant, S. S. Iyengar, J. Tomasi, M. Cossi, J. M. Millam, M. Klene, C. Adamo, R. Cammi, J. W. Ochterski, R. L. Martin, K. Morokuma, O. Farkas, J. B. Foresman, and D. J. Fox **2016**.
[39] C. I. Bayly, P. Cieplak, W. Cornell, P. A. Kollman, “A well-behaved electrostatic potential based method using charge restraints for deriving atomic charges: the RESP model” *The Journal of Physical Chemistry* **1993**, *97*, 10269–10280.
[40] P. A. K. D.A. Case, H.M. Aktulga, K. Belfon, I.Y. Ben-Shalom, S.R. Brozell, D.S. Cerutti, T.E. Cheatham, III, G.A. Cisneros, V.W.D. Cruzeiro, T.A. Darden, R.E. Duke, G. Giambasu, M.K. Gilson, H. Gohlke, A.W. Goetz, R. Harris, S. Izadi, S.A. Izmailov, C. Jin, K. Ka **2018**.
[41] P. V. Klimovich, M. R. Shirts, D. L. Mobley, “Guidelines for the analysis of free energy calculations” *Journal of Computer-Aided Molecular Design* **2015**, *29*, 397–411.

[42] D. R. Roe, T. E. I. Cheatham, "PTRAJ and CPPTRAJ: Software for Processing and Analysis of Molecular Dynamics Trajectory Data" *J. Chem. Theory Comput.* **2013**, *9*, 3084–3095.
[43] P. A. K. D.A. Case, H.M. Aktulga, K. Belfon, I.Y. Ben-Shalom, S.R. Brozell, D.S. Cerutti, T.E. Cheatham, III, G.A. Cisneros, V.W.D. Cruzeiro, T.A. Darden, R.E. Duke, G. Giambasu, M.K. Gilson, H. Gohlke, A.W. Goetz, R. Harris, S. Izadi, S.A. Izmailov, C. Jin, K. Ka **2022**.
[44] T. Lazaridis, "Inhomogeneous Fluid Approach to Solvation Thermodynamics. 1. Theory" *J. Phys. Chem. B* **1998**, *102*, 3531–3541.
[45] C. N. Nguyen, T. Kurtzman Young, M. K. Gilson, "Grid inhomogeneous solvation theory: Hydration structure and thermodynamics of the miniature receptor cucurbit[7]uril" *The Journal of Chemical Physics* **2012**, *137*, 044101.
[46] A. Walser, T. Flynn, C. Mason, H. Crowley, C. Maresca, B. Yaremko, M. O'Donnell, "Triazolobenzo- and triazolothienodiazepines as potent antagonists of platelet activating factor" *J. Med. Chem.* **1991**, *34*, 1209–1221.
[47] V. A. Chornous, A. N. Grozav, M. V. Vovk, "Convenient synthesis of 3-chloroimidazo[1,5-a]quinoxalines" *Russ J Org Chem* **2017**, *53*, 474–476.
[48] B. Albrecht, D. Bauer, S. Bellon, C. Bode, S. Booker, A. Boezio, D. Choquette, D. D'amico, J.-C. Harmange, S. Hirai, R. Hungate, T.-S. Kim, R. Lewis, L. Liu, J. Lohman, M. Norman, M. Potashman, A. Siegmund, S. Springer, M. Stec, N. Xi, K. Yang, *Fused Heterocyclic Derivatives Useful as Inhibitors of the Hepatocyte Growth Factor Receptor*, **2008**, WO2008008539A2.
